# Salicylideneaniline/Dithienylethene Hybrid Molecular Switches: Design, Synthesis, and Photochromism

**DOI:** 10.1021/acs.joc.3c00828

**Published:** 2023-12-07

**Authors:** Péter Pál Kalapos, Attila Kunfi, Marcell M. Bogner, Tamás Holczbauer, Michał Andrzej Kochman, Bo Durbeej, Gábor London

**Affiliations:** † MTA TTK Lendület Functional Organic Materials Research Group, Institute of Organic Chemistry, Research Centre for Natural Sciences, Magyar Tudósok Krt. 2, 1117 Budapest, Hungary; ‡ Institute of Organic Chemistry, Centre for Structural Science, Research Centre for Natural Sciences, Magyar Tudósok Krt. 2, 1117 Budapest, Hungary; § Institute of Physical Chemistry, Polish Academy of Sciences, Marcina Kasprzaka 44/52, 01-224 Warsaw, Poland; ∥ Division of Theoretical Chemistry, IFM, Linköping University, SE-58183 Linköping, Sweden

## Abstract

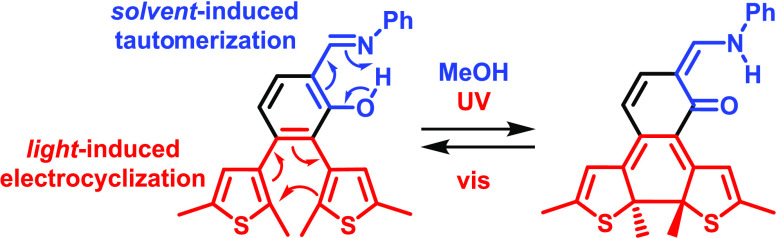

A hybrid molecular switch comprising salicylideneaniline (SA) and dithienylethene (DTE) moieties around a single benzene ring is reported. Due to an interplay between solvent-assisted enol–keto tautomerization in the former moiety and photochromic electrocyclization in the latter, this dithienylbenzene derivative was found to be photoresponsive at room temperature with a thermally stable closed form. The main photoproduct featuring ring-closed DTE and keto-enamine SA structures could be isolated and converted back to the starting material by irradiation with visible light. The optical properties of the potential structures involved in the overall process were characterized by using density functional theory (DFT) calculations in good agreement with the measured data. The reversibility of the conversion could be tuned by the presence of donor and acceptor substituents, while the introduction of the imine in the form of a benzothiazole moiety enabled photochemistry even in nonprotic solvents.

## Introduction

**Figure 1:**
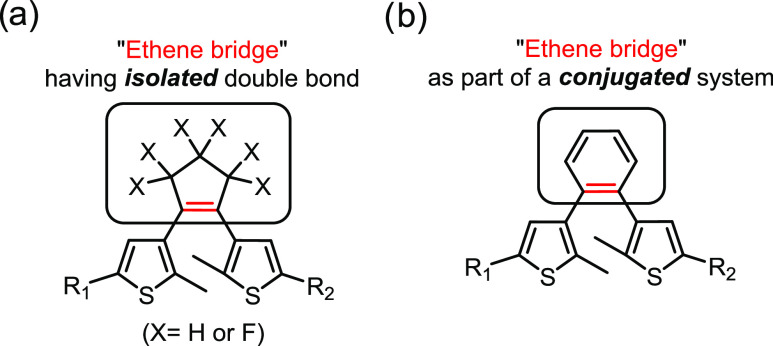
General structures of (a) dithienylethenes and (b) dithienylarenes.

Dithienylethenes (DTEs) are among the most frequently used molecular photoswitches.^1−3^ Their applications span the fields of molecular electronics,^4,5^ responsive materials,^6,7^ catalysis,^8^ and photopharmacology.^9,10^ So far, the design of DTEs has primarily involved ethene bridges containing an isolated or strongly localized double bond (Figure 1a),^1,11−16^ mainly as part of heterocyclic ring systems in the latter case. The use of aromatic linkers (Figure 1b) is scarcely explored,^17−21^ although they could be part of π-extended molecular materials with controllable conjugation patterns or could be used to tune π-interactions in supramolecular systems. Notably, the first application of a dithienylarene as part of an optical sensor under a high backlight intensity has recently been reported.^22^

The neglect of dithienylbenzene derivatives as potential photochromic compounds reflects the assumption that the loss of ground-state aromaticity in three aryl rings upon ring closing would prevent efficient photoswitching.^15^ Although this reasoning steered research interest away from dithienylbenzene derivatives, we recently demonstrated that their excited-state properties do indeed permit photoswitching.^23^ Specifically, we showed that electronically unperturbed diarylethene switches with an aromatic benzene bridge connecting two thienyl units undergo photoinduced electrocyclization driven by excited-state antiaromaticity,^24−26^ in accordance with Baird’s rule^27^ (the reverse of Hückel’s rules for ground-state aromaticity).

Although, formally, this reaction of dithienylbenzene is similar to that of photoswitches possessing nonconjugated linkers, dithienylbenzenes are truly distinct in two important aspects: (i) the underlying mechanism of operation involves changes in aromaticity between ground and excited states, and (ii) the π-conjugated nature of the benzene ring allows for transmission of (photo)chemically generated electronic changes between different parts of the molecule. Based on these guiding points, we have begun to explore the fundamental properties of dithienylbenzene derivatives along with possibilities to control electronic interactions across the π-system of the benzene ring. For example, we have recently shown that π-extension of the benzene ring into a biphenylene ring system results in a dithienylarene that, upon photoswitching, is able to reversibly alter its local (anti)aromatic features.^28^

**Scheme 1:**
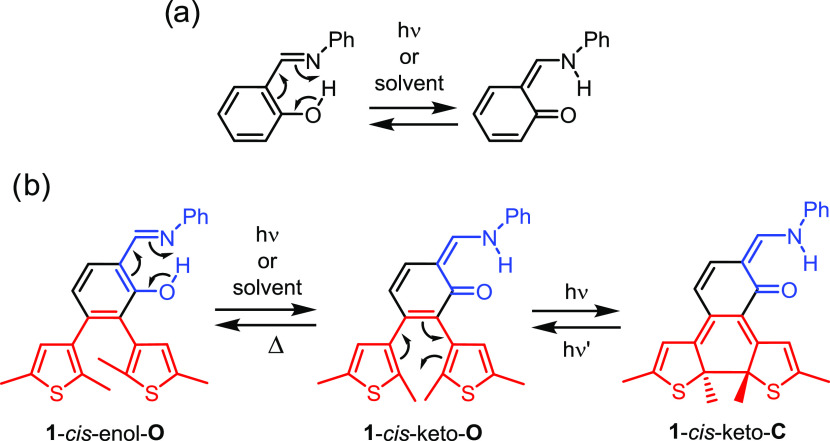
(a) Structure and Tautomerization of SA. (b) Structure and Tentative Mechanism of an SA-DTE Hybrid Photoswitch

In order to further explore the photochemistry of dithienylbenzene for the development of novel photoswitchable architectures, we envisioned fusing it with the salicylideneaniline (SA) chromophore.^29^ Salicylideneanilines undergo intramolecular proton transfer upon light irradiation, leading to a reversible enol-imine/keto-amine tautomerization (Scheme 1a).^30−36^ Thus, the functionalization of SA with thienyl units would provide an interesting multistate switch (Scheme 1b). Initially, the SA subunit would be in its enol form along with the open form of the DTE (**1**-*cis*-enol-**O**; see also Figure S9 and Table S2 in the Supporting Information for a catalog of the relevant isomers of compound **1** and their relative energies, respectively). Upon absorption of light, ultrafast excited-state proton transfer would transform the SA unit into its keto-form (**1**-*cis*-keto-**O**). Alternatively (or complementarily), solvent-assisted tautomerization could also contribute to the formation of the keto structure.^32,37−40^ Following this transformation, the initially aromatic benzene ring would adopt a cyclohexa-1,3-diene-type arrangement. After such a dearomatization, the stage would be set for a facile electrocyclization of the DTE subunit, leading to the formation of **1**-*cis*-keto-**C**. In the present work, we report on the synthesis of this potential hybrid photoswitch and assess its mechanism both spectroscopically and computationally.

## Results and Discussion

In the following sections, we first present the synthesis of **1**-*cis*-enol-**O** and then ultraviolet–visible (UV–vis) spectroscopic analysis of its photochemical properties, including its solvatochromism and solvent-dependent photoresponse upon light irradiation. These measurements are supported by calculated photoabsorption spectra of the different species potentially present in solution before and after irradiation. Structural insights into the photoproducts are provided based on ^1^H NMR spectroscopic data. To improve the reversibility of the switching, various derivatives of **1**-*cis*-enol-**O** with donor and acceptor substituents are also studied.

### Synthesis of 1-*cis*-Enol-**O**

**Scheme 2:**
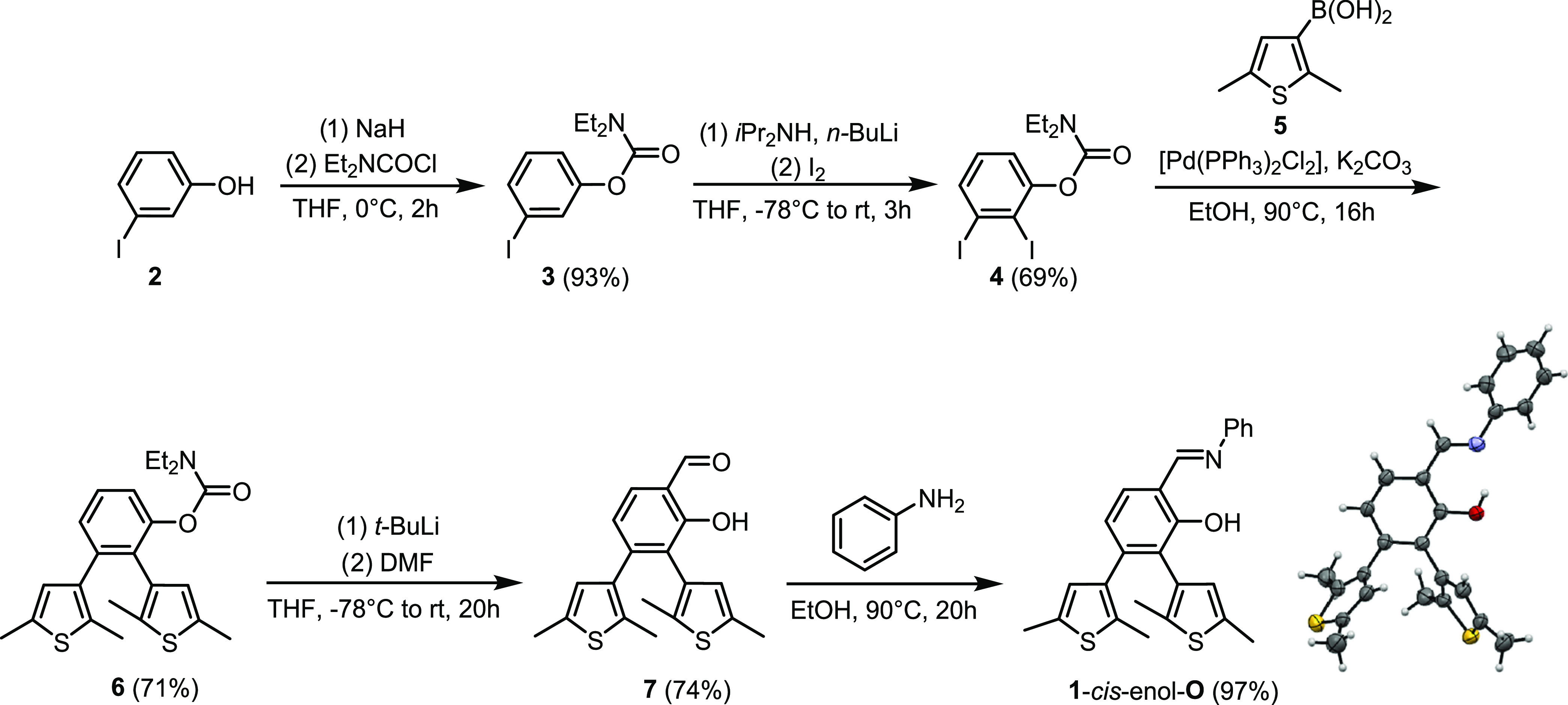
Synthesis and X-ray Crystal Structure of **1**-*cis*-Enol-**O** ORTEP style representations are drawn at the 50% probability level.

**1**-*cis*-Enol-**O** was synthesized in five steps (Scheme 2). The first step was the introduction of a diethylcarbamate protecting group into 3-iodophenol. The resulting compound (**3**) was selectively iodinated via an *ortho*-lithiation process that led to diiodo derivative **4**. Suzuki–Miyaura coupling between **4** and thienylboronic acid **5** provided dithienylarene structure **6**. This latter step of the synthesis was complicated by the fact that the monothienylated compound was formed as a side product. This compound proved difficult to separate from the desired compound **6**, necessitating a series of column chromatographic purifications. Diethylcarbamate was used as a directing group to introduce a formyl group into compound **6** by *ortho*-lithiation to access aldehyde **7**. Notably, during formylation, the removal of the protecting group was also realized. As the last step of the synthesis, the Schiff base motif of **1**-*cis*-enol-**O** was obtained from aldehyde **7** and aniline as the amine partner.

The structure of **1**-*cis*-enol-**O** was verified with the use of ^1^H and ^13^C NMR spectroscopies, as well as with single-crystal X-ray diffraction measurements (Scheme 2 and Section S2, Supporting Information). Notably, **1**-*cis*-enol-**O** crystallized with the thienyl units in a parallel conformation, which is photochemically inactive and hinders the isomerization of DTEs in the solid state. However, the conversion of this conformation into a photochromic, antiparallel conformation is expected to occur rapidly in solution at ambient conditions.

### UV–vis and ^1^H NMR Spectroscopic Characterization of the Photoresponse of 1-*cis*-Enol-**O**

**Figure 2:**
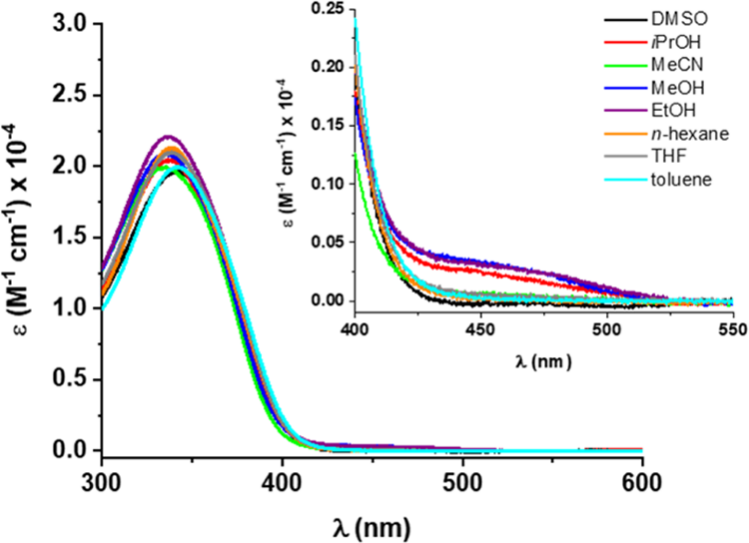
UV–vis spectra of **1**-cis-enol-**O** in different solvents at rt.

The photochemical properties of **1**-*cis*-enol-**O** were investigated by UV–vis and ^1^H NMR spectroscopies. Its UV–vis absorption spectra were recorded in different aprotic and protic solvents to investigate the potential influence of solvent interactions on the tautomeric equilibrium between the enol and keto forms present in the SA motif (Figure 2). In most solvents, the spectra showed a high-intensity band at 345 nm. In protic solvents (MeOH, EtOH, and *i*PrOH), a new band appeared at the edge of the visible region around 450 nm.

**Figure 3:**
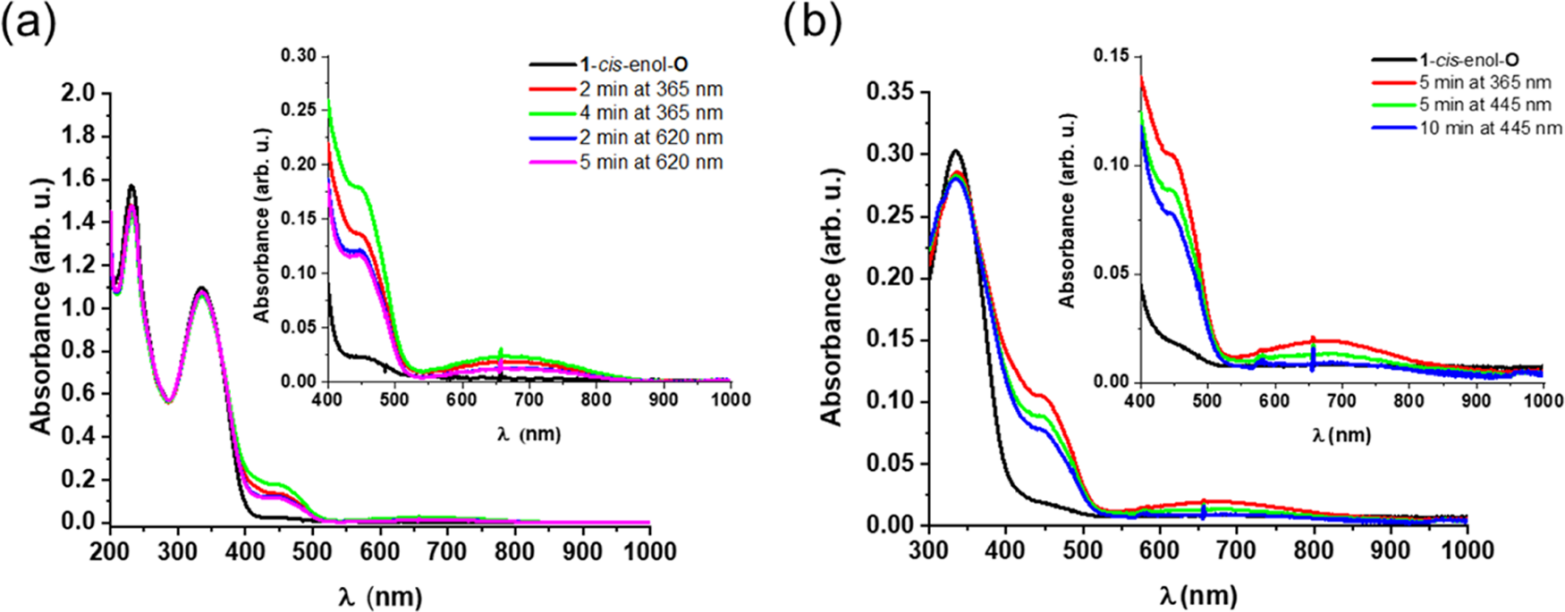
Irradiation of **1**-*cis*-enol-**O** in MeOH with 365 nm light at rt and subsequent irradiation with (a) 620 and (b) 445 nm light at rt.

Similar solvatochromism has previously been described for salicylideneanilines, for which the longer wavelength absorption bands in protic solvents are associated with the formation of small amounts of the keto-amine tautomer.^39−41^ The observed solvatochromism for **1**-*cis*-enol-**O** is in line with its photochemical response in different solvents (Figure 3). Indeed, while UV light irradiation in MeOH resulted in pronounced changes in the UV–vis spectrum, in acetonitrile, no such changes could be detected (Figure S3, Supporting Information). Specifically, irradiation of **1**-*cis*-enol-**O** in MeOH with 365 nm light increased the intensity of the band at 445 nm and produced a very broad, new band centered at 680 nm (Figure 3a). Subsequent irradiation of the solution with 620 nm light resulted in a loss in the intensity of the new band; however, the initial spectrum could not be completely regenerated. The spectral changes were accompanied by visible changes as well: the pale-yellow solution turned deep yellow upon UV light irradiation (Figure S2, Supporting Information). Notably, irradiation with both 620 and 445 nm light led to reversibility (Figure 3b). Further UV–vis studies revealed that irradiation with 445 nm light not only induces the ring opening process but also affects the ring closing reaction of **1**-*cis*-enol-**O**. Irradiation with this wavelength results in the formation of a minor amount of the closed form (Figure S4, Supporting Information).

Since the emergence of a broad, red-shifted visible absorption is characteristic of the closed forms of DTEs, these results indicate that **1**-*cis*-enol-**O** undergoes photoinduced electrocyclization in MeOH to yield **1**-*cis*-enol-**C** or **1**-*cis*-keto-**C**. The photoreaction appears to be reversible in that visible light irradiation (620 or 445 nm) can revert some of the observed changes. However, the original spectrum cannot be fully regenerated, indicating that multiple photochemically (and/or solvent) induced processes operate under the explored conditions.

**Figure 4:**
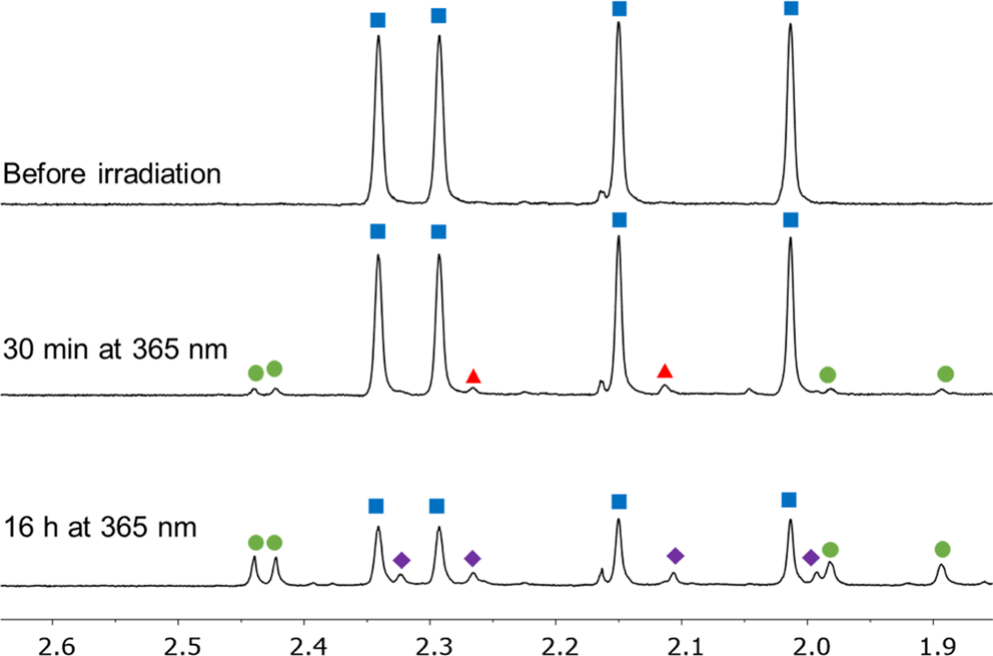
Irradiation of **1**-*cis*-enol-**O** in CD_3_OD by UV light at rt followed by ^1^H NMR spectroscopy (500 MHz) (ppm scale). Signals of the starting material are marked in blue; signals of the major new component are marked in green; and signals of the two new minor components are marked in red and purple colors.

To gain structural insight into the photoproducts, we performed ^1^H NMR analysis. Accordingly, **1**-*cis*-enol-**O** was irradiated with UV light in a quartz NMR tube in CD_3_OD (Figure 4). Notably, in the concentration range ideal for ^1^H NMR measurements (0.01–0.005 mM), substantially longer reaction times were required to observe appreciable amounts of photoproducts. During the irradiation, the pale-yellow solution turned green. After 30 min of irradiation, two new sets of signals appeared in the ^1^H NMR spectrum (Figure 4, 30 min). The intensity of the major set of new methyl signals (Figure 4, green color) increased during the reaction, while other photoproducts also appeared upon further irradiation (Figure 4, purple color), which are structurally similar to each other (for further details, see Section S5 in the Supporting Information).

After prolonged irradiation (up to 16 h), substantial conversion to the main product (Figure 4, green) was achieved. This compound had highly shifted methyl signals compared to those of **1**-*cis*-enol-**O**, suggesting that it contains the closed form of the DTE moiety. Conversely, the other minor photoproduct (Figure 4, purple color) had ^1^H resonances at similar chemical shift values compared to the starting material. It should be noted that in accordance with the UV–vis measurements, irradiation in CD_3_CN did not produce any new products even after 16 h of irradiation (Figure S11, Supporting Information).

Part of the major photoproduct (Figure 4, green color) could be isolated and its ^1^H NMR spectrum could be recorded (C_6_D_6_). Even though the sample was contaminated with a small amount of the starting material, ^1^H NMR measurements revealed that the isolated structure is either **1**-*cis*-keto-**C** or **1**-*trans*-keto-**C** (Figure S14, Supporting Information). In other words, the stereochemistry around the C1–C7 bond (Figure S9, Supporting Information) could not be unambiguously determined.

**Figure 5:**
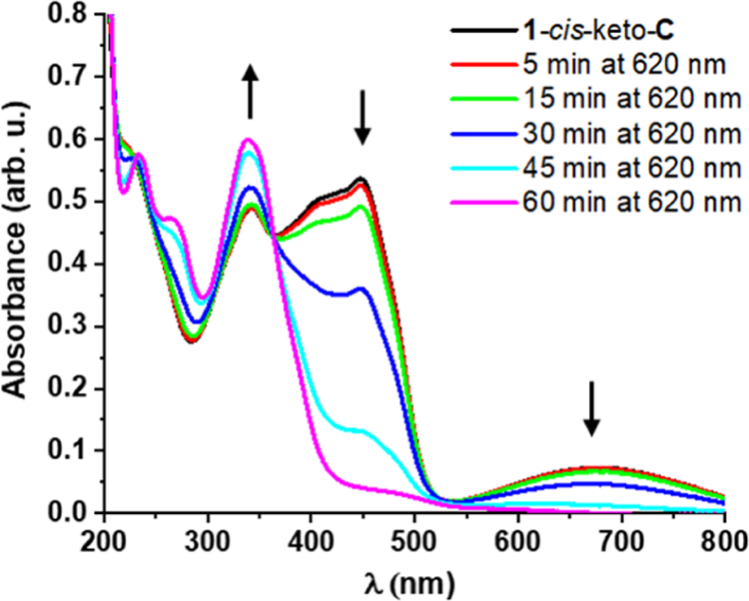
UV–vis spectra (c = approximately 10^–5^ M, rt) of the isolated photoproduct **1-**cis/trans-keto**-C** and its subsequent irradiation with 620 nm light at rt.

The isolated green, major photoproduct was also subjected to UV–vis measurements. Its absorption spectrum in MeOH (Figure 5) showed an intense band at around 450 nm and a broad band centered at 680 nm, which are features that agree well with those in the UV–vis spectrum obtained by the irradiation of **1**-*cis*-enol-**O** (Figure 4). Hence, the different color of the NMR sample (green) compared to that of the UV–vis sample (dark yellow) upon irradiation with UV light is due to the difference in concentration between the two samples. Irradiation of the isolated material with 620 nm light resulted in the disappearance of these bands and an increase in absorption intensity at 360 nm (Figure 5). Notably, full conversion required a relatively long irradiation time (1 h). Importantly, the spectrum of the irradiated photoproduct matches the spectrum of the initial, open form of the molecule. This clearly demonstrates that the combined SA-DTE system is photochromic and that reversible conversion between the two isomers can be achieved by UV and visible light irradiation.

### Solid-State Properties of **1**-*cis*-Enol-**O**

**Figure 6:**
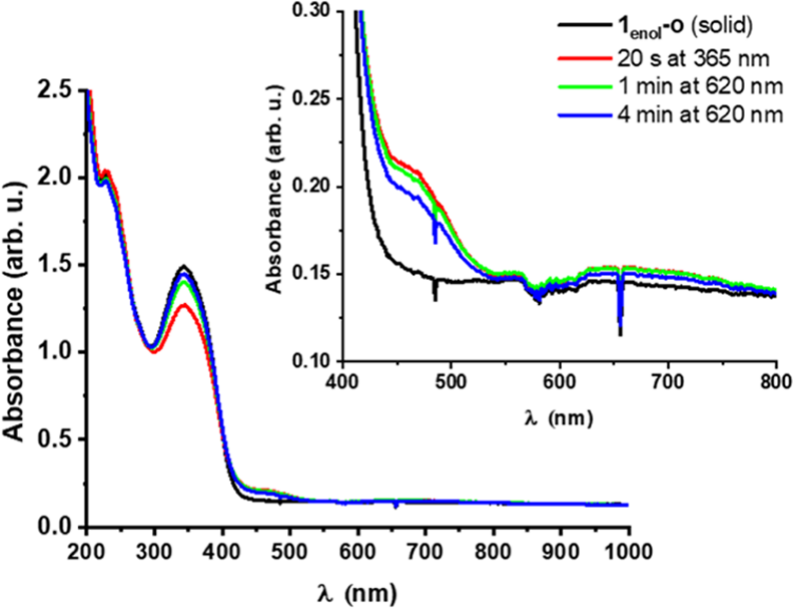
UV–vis absorption spectra of **1**-*cis*-enol-**O** in the solid state at rt. A film of the compound was prepared by allowing a concentrated solution in DCM to evaporate on the inside wall of a quartz cuvette.

Photo- and thermochromism has been reported for several SA-type molecules in the solid state.^29,42^**1**-*cis*-Enol-**O**, as a solid, did not show any color change upon cooling to −78 °C. The solid-state UV–vis absorption spectra of **1**-*cis*-enol-**O** (rt) exhibited a small “shoulder” around 450 nm, similar to the situation in protic solvents and indicating the presence of the tautomeric **1**-*cis*-keto-**O** (Figure 6). Irradiation of **1**-*cis*-enol-**O** in the solid state resulted in the appearance of new absorption bands at 650 nm and a decrease in absorption intensity at 350 nm. When the film was subsequently irradiated with visible light (620 nm), the intensity of the new bands decreased, but only to a small extent (Figure 6). Due to the similarity between these spectral changes and those that were observed in MeOH, we can conclude that **1**-*cis*-enol-**O** preserves its ability to photoisomerize also in the solid state, however, only to a small extent. The solid-state photoprocess is likely limited by the nonphotochromic parallel conformation of the switch within the film, the presence of which can be inferred from the crystal structure of **1**-*cis*-enol-**O** (Scheme 2).

### Consideration of Control Structures and Potential Side Reactions

**Figure 7:**
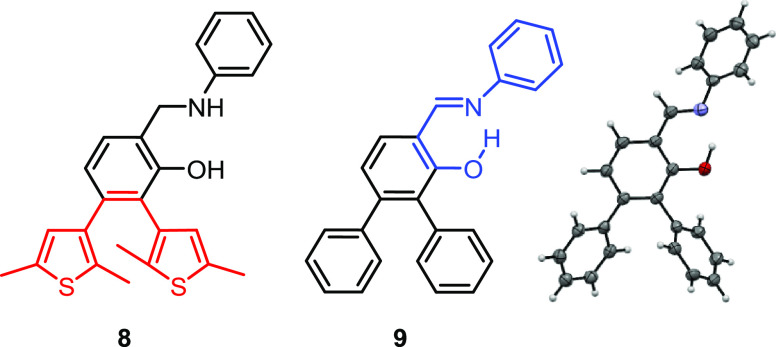
Structures of control compounds **8** and **9** and the X-ray crystal structure of **9**. (ORTEP style representations are drawn at the 50% probability level).

To confirm that both the SA and DTE motifs are needed within the same molecule for the observed photoreactivity, we also synthesized control compounds **8** and **9** (Figure 7). Among these, an X-ray crystal structure could be obtained for **9** (Figure 7 and Section S2, Supporting Information). In **8**, interruption of the electronic communication between the nitrogen atom and the central benzene ring is expected to rule out a photoresponse. Indeed, the lack of this interaction would prevent the formation of the dearomatized oxo-tautomer (**1**-*cis*-keto-**O**, for the case of compound **1**) that is considered to be key for the observed isomerization. In **9**, the photoactive DTE motif is replaced by unreactive phenyl rings. Pleasingly, UV–vis measurements revealed that none of the compounds **8** and **9** show any observable photoresponse at rt. Moreover, irradiation with UV light did not produce any well-defined photoproducts (Figure S5 and S6, Supporting Information).

**Scheme 3:**
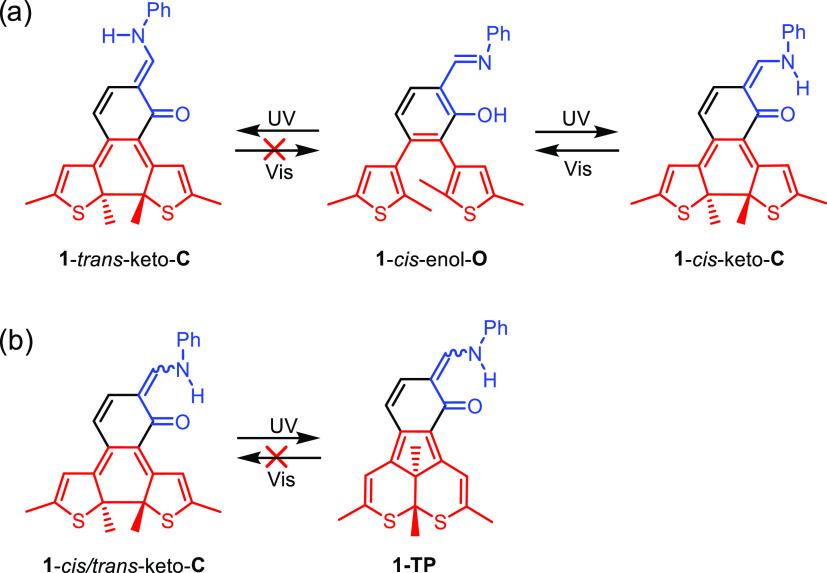
Possible Light-Induced Side Reactions During the Transformation of **1**-*cis*-enol-**O** Irreversible formation of (a) *trans*-enamine **1**-*trans*-keto-**C** and (b) a thiopyran derivative **1-TP**.

Up to this point, the photochemical experiments suggest that UV irradiation of **1**-*cis*-enol-**O** in MeOH induces both intramolecular proton transfer to produce **1**-*cis*-keto-**O** and photocyclization to produce **1**-*cis*-keto-**C**. This picture is corroborated by complementary electronic structure calculations summarized in Section S4 in the Supporting Information. Besides yielding detailed insights into the isomerism shown by compound **1**, these calculations also provide photoabsorption spectra of the relevant isomers that support the assignments made based on the experimental UV–vis measurements. Importantly, the photochemical experiments also show that the conversion of **1**-*cis*-enol-**O** into **1**-*cis*-keto-**C** is reversible in MeOH, because when **1**-*cis*-keto-**C** was isolated and irradiated with visible light, UV–vis spectroscopy confirms the formation of **1**-*cis*-enol-**O**. Overall, however, UV irradiation of **1**-*cis*-enol-**O** in MeOH led to a mixture of products, some of which did not show reversible photochemistry upon irradiation with visible light. Unfortunately, the small amount of photoproducts formed even after long irradiation times did not facilitate their isolation and structural characterization. As the UV–vis and ^1^H NMR spectra suggest the formation of photoproducts that are structurally related to **1**-*cis*-keto-**C**, it seems reasonable to consider (at least) two processes that could interfere with a clean **1**-*cis*-enol-**O** → **1**-*cis*-keto-**C** transformation (Scheme 3).

One such process is *cis*/*trans* photoisomerization within the SA moiety to produce both **1**-*cis*-keto-**C** and **1**-*trans*-keto-**C**.^31,32,35,43^ Specifically, visible light irradiation can only convert the *cis* isomer back to the **1**-*cis*-enol-**O** form, because in the *trans*-isomer, the reactive N–H bond is positioned away from the ketone group (Scheme 3a). The second side reaction that can be envisioned is the UV-induced rearrangement of the DTE moiety into a thiopyrane derivative (**1-TP**) (Scheme 3b), which is a well-documented source of fatigue in DTE-type switches.^44,45^ (Notably, the potential contribution of **1**-*trans*-keto-**O** to the measured spectra could not be excluded either.) Interestingly, the formation of **1-TP**-type products has recently been considered a potentially useful one-way switching process.^46,47^

### Addressing the Tautomerization-Gated Mechanism to Improve Fatigue Resistance

**Figure 8:**
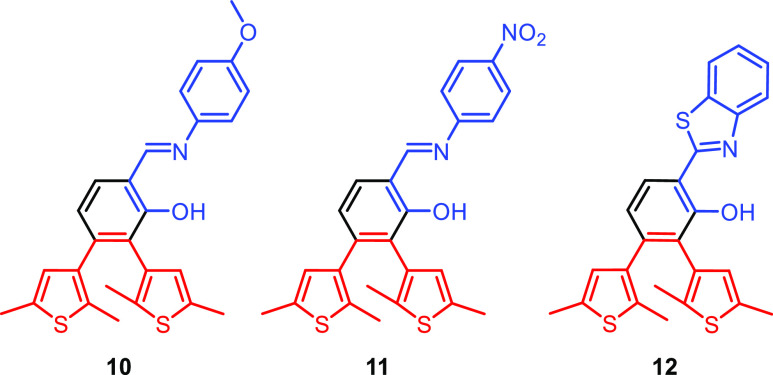
Molecules prepared to probe substituent effects on the photoreaction.

It is well known from the literature that the position of the enol-imine/keto-amine tautomeric equilibrium in SA molecules can be influenced by the choice of the solvent and, more importantly, by modifying the basicity of the reactive nitrogen atom.^38,39^ Based on the observed solvent effect, it seems reasonable to assume that the photocyclization of the DTE subunit in MeOH proceeds mainly from the small amount of **1**-*cis*-keto-**O** present in solution. Consequently, we synthesized and investigated the photochromic behavior of different derivatives of **1**-*cis*-enol-**O** where the phenyl substituent of the imine nitrogen is equipped with either an electron-donating or electron-withdrawing substituent (Figure 8). We expected that by increasing the electron density at the reactive nitrogen, the tautomeric equilibrium would be shifted toward the keto form, which, based on the aforementioned reasoning, in turn would enhance the photoreactivity. To test this idea, we synthesized compound **10** having an electron-donating methoxy substituent and compound **11** having an electron-withdrawing nitro substituent. Furthermore, compound **12** bearing a benzothiazole moiety was also prepared. Based on previously reported data, photochromism in the presence of this unit would be facilitated even in aprotic solvents.^48^

**Figure 9:**
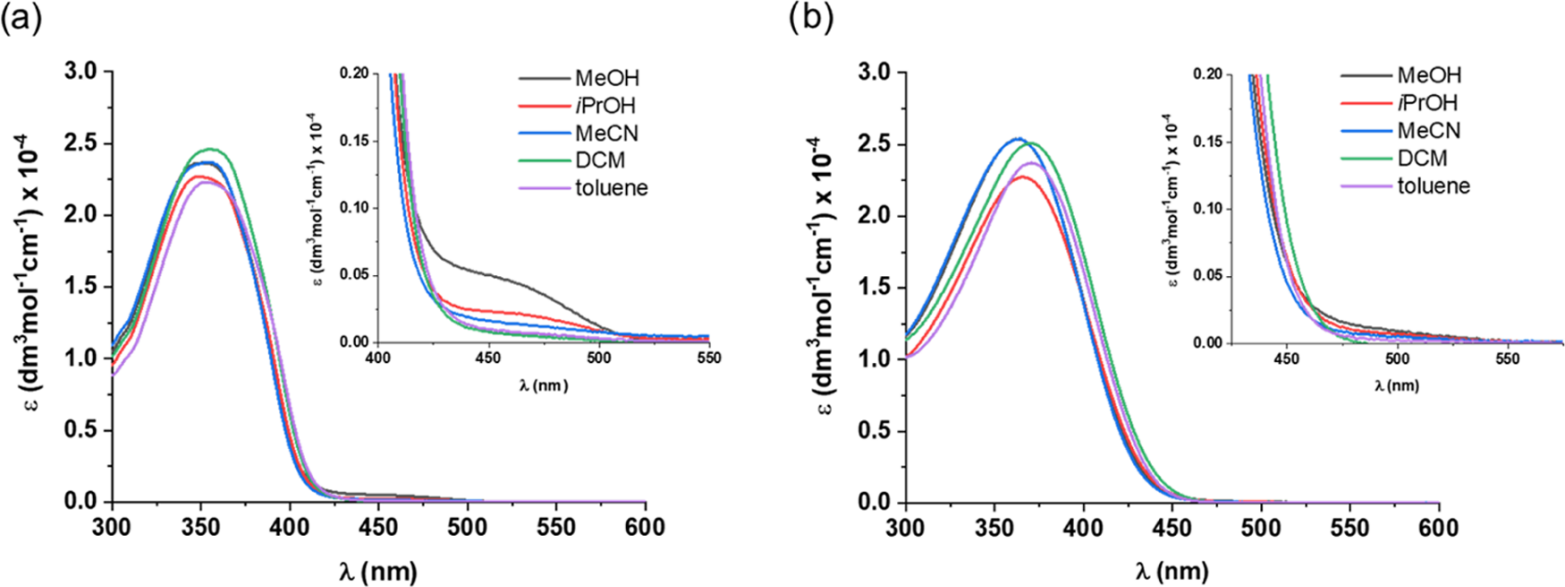
UV–vis spectra of (a) **10** and (b) **11** in different solvents at rt.

UV–vis measurements of **10** revealed solvatochromic behavior similar to that of **1**-*cis*-enol-**O** (Figure 9a). In aprotic solvents, an absorption band at 360 nm dominated the spectra, while in MeOH a new band centered at 450 nm appeared alongside the main band at 350 nm. **11**, on the other hand, displayed a different behavior in protic solvents compared to **1**-*cis*-enol-**O** and **10**. Specifically, while in aprotic solvents an intense band centered at 370 nm was observed, in MeOH no absorption appeared at 450 nm (Figure 9b).

**Figure 10:**
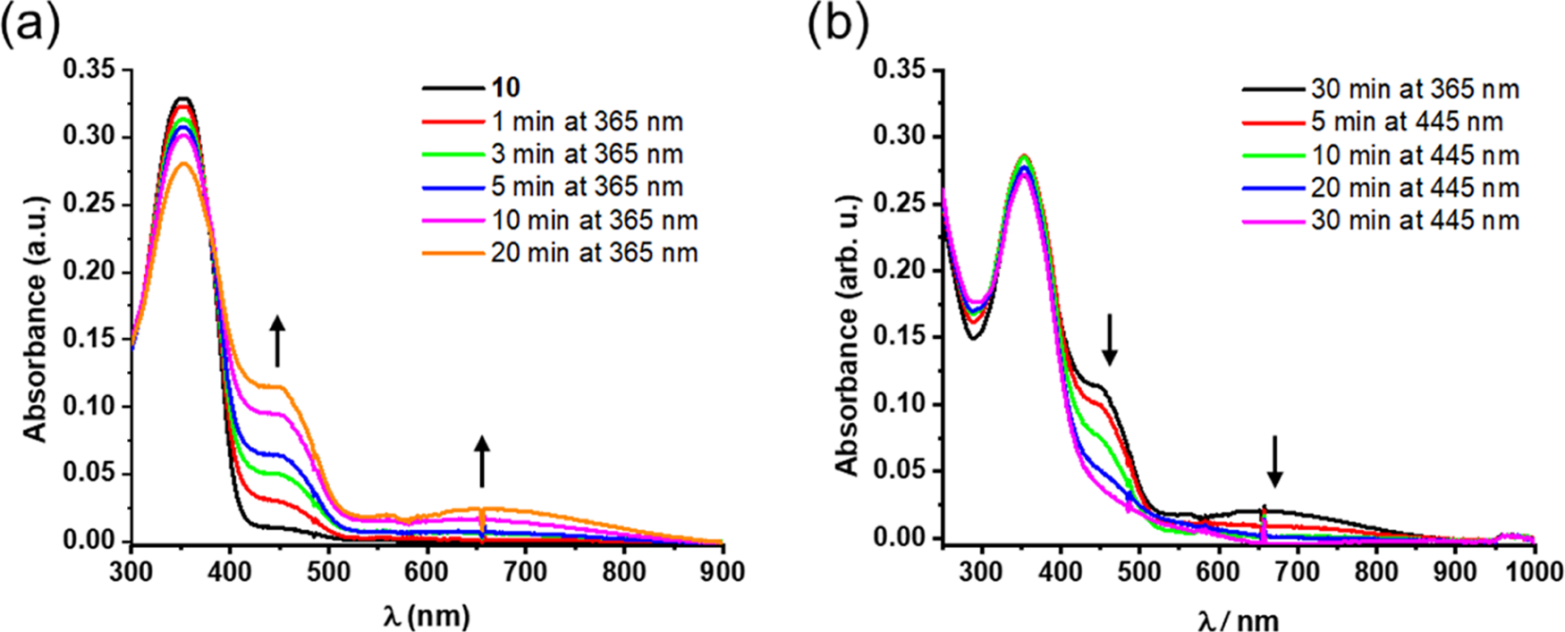
UV–vis spectra of **10** upon irradiation with (a) UV and (b) visible light at rt (MeOH).

In accordance with these spectral features, irradiation of **10** in MeOH yielded a photoresponse similar to that of **1**-*cis*-enol-**O**, whereas **11** only showed signs of degradation (Figure S7, Supporting Information). Upon irradiation of **10**, new absorption bands appeared at 450 and 650 nm. Moreover, these changes were somewhat more intense than those in the case of **1**-*cis*-enol-**O** (Figure 10a). Also, when the resulting solution was irradiated with visible light (445 nm), the initial absorption could largely be regenerated in the visible region, but some broad absorptions remained at around 450 nm (Figure 10b). It should be noted that the residual absorptions after prolonged visible light irradiation are very broad, suggesting that partial photodegradation most likely took place.

**Figure 11:**
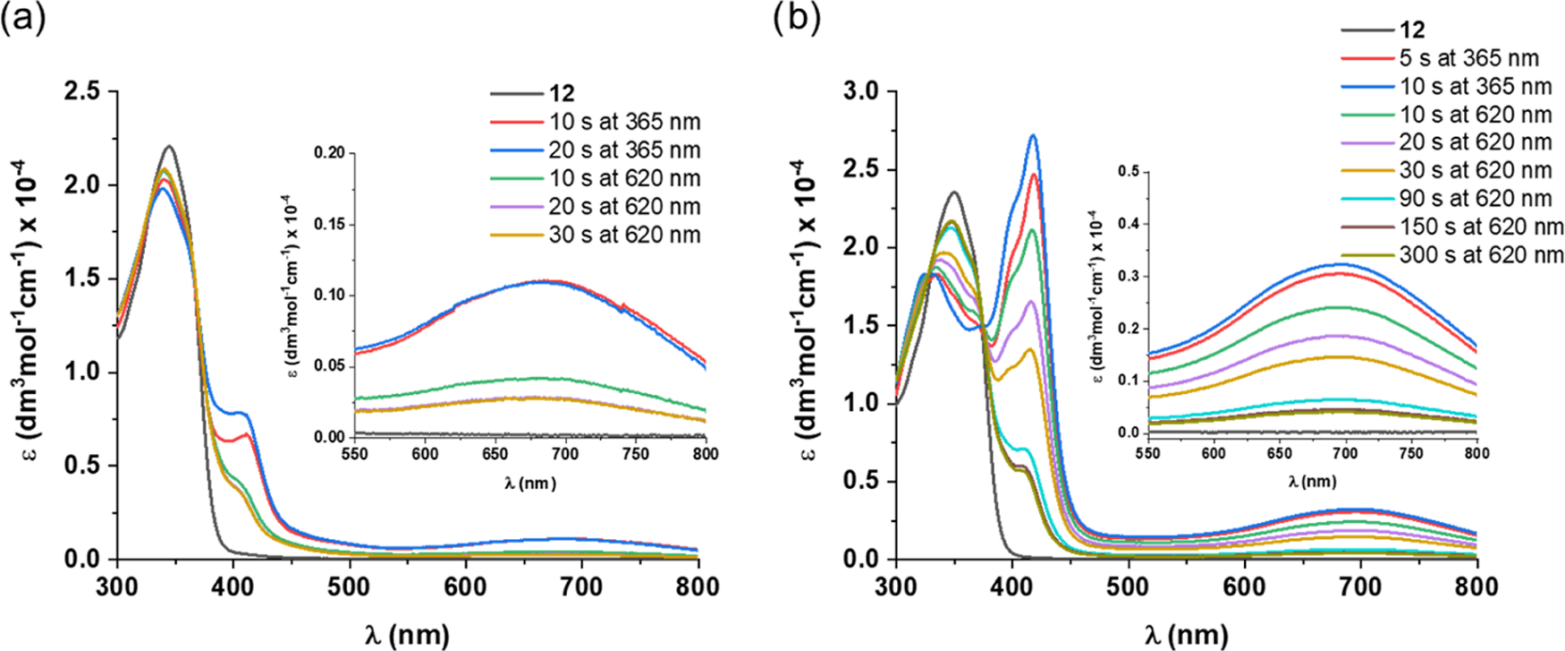
UV–vis spectra of **12** upon irradiation with UV and visible light in (a) MeOH and (b) toluene at rt.

Compound **12** featuring a benzothiazole moiety showed photoreactivity in both protic (MeOH) and aprotic (toluene) solvents (Figure 11), and its isomerization was somewhat faster compared to the simple imines described above. Furthermore, compared to **1**-*cis*-enol-**O** and **10**, its photoproduct was found somewhat more resistant to thermal degradation (Figure S8, Supporting Information). These differences are likely associated with the more rigid and electronically different heteroaromatic benzothiazole unit. However, the reversibility of the process was similarly incomplete, as in the previous examples. Nevertheless, **12** enables probing of the photoswitching mechanism through ^1^H NMR/FT-IR-based monitoring of the changes of the chemical environment around the reactive phenolic O–H group. All in all, we believe that the results of this work show that further optimization and characterization of the SA-DTE system are very worthwhile endeavors.

## Conclusions

In summary, we have shown that it is possible to fuse SA and DTE motifs in a hybrid molecular switch that is capable of reversible photochromism at rt. As evidenced by UV–vis and NMR spectroscopies, such a molecule (**1**-*cis*-enol-**O**) undergoes solvent-dependent photoswitching through an intermediary keto tautomer that is stabilized by protic solvents. The absorption properties of the species potentially present in solution were calculated in good agreement with the experimental observations. In an attempt to tune the reversibility of the process, donor and acceptor substituents were introduced at the phenyl ring of the imine nitrogen. While a methoxy group led to better reversibility based on UV–vis measurements, a nitro substituent prevented photoconversion. Interestingly, the introduction of the imine function in the form of a benzothiazole moiety enabled photochemistry even in nonprotic solvents. It is envisioned that these compounds will find use as photochromic Schiff-base ligands or Salen frameworks in future light-controlled catalytic^49^ and material^50^ applications.

## Experimental Section

### General Information

Commercial reagents, solvents, and catalysts (Aldrich, Fluorochem, VWR) were purchased as reagent-grade and used without further purification. Solvents for extraction or column chromatography were of technical quality. For spectroscopy and sample treatment, Opti-Grade quality solvents were used. Organic solutions were concentrated by rotary evaporation at 25–40 °C. Thin-layer chromatography was carried out on SiO_2_-layered aluminum plates (60778–25EA, Fluka). Column chromatography was performed using SiO_2_–60 (230–400 mesh ASTM, 0.040–0.063 mm from Merck) at 25 °C or using a Teledyne Isco CombiFlash Rf+ automated flash chromatographer with silica gel (25–40 μm, Redisep Gold). Room temperature refers to 20–25 °C depending on the time of the day.

NMR spectra were acquired on a Varian 500 NMR spectrometer running at 500 and 126 MHz for ^1^H and ^13^C, respectively. The residual solvent peaks were used as the internal reference. Chemical shifts (δ) are reported in ppm. The following abbreviations are used to indicate the multiplicity in ^1^H NMR spectra: s, singlet; d, doublet; t, triplet; q, quartet; p, pentet; and m, multiplet. ^13^C NMR spectra were acquired on broadband decoupled mode.

Mass spectrometric measurements were performed using a Sciex 5600+ TripleTOF high-resolution mass spectrometer (Sciex, Massachusetts) equipped with a DuoSpray source operated in ESI or APCI mode. The resolution of the instrument was above 35,000 over the entire mass range. Samples were measured in flow injection mode using acetonitrile as the mobile phase at a flow rate of 0.2 mL/min.

UV–vis spectrophotometry was executed on a Jasco V-750 or a PerkinElmer Lambda 465 spectrophotometer. Hellma Analytics high-precision quartz cuvettes were used with an optical path length of 1.0 cm. Irradiation of samples was carried out with 10 W COB LED light sources operated at 1 A electric current. All irradiation experiments were performed under a N_2_ atmosphere.

#### Synthesis of 3-Iodophenyl Diethylcarbamate (**3**)

NaH (0.96 g, 24 mmol, 1.2 equiv, 60 wt % dispersion in mineral oil) was suspended in dry THF (20 mL) under a N_2_ atmosphere and cooled to 0 °C (ice bath). To this suspension, a solution of 2-iodophenol (**2**) (4.4 g, 20 mmol, 1.0 equiv) in THF (6 mL) was added, and the reaction mixture was stirred for 15 min at 0 °C (until the bubbling stopped). Subsequently, N,N-diethylcarbamoyl chloride (5.1 mL, 40 mmol, 2.0 equiv) in THF (5 mL) was added and the reaction mixture was stirred for 2 h. After the reaction was completed, water was added, and the mixture was extracted with EtOAc. The organic layer was washed with water and brine, dried over MgSO_4_, and concentrated under vacuum. The crude product was further purified by column chromatography (SiO_2_, hexane/EtOAc 5:1) to obtain compound **3** as a colorless liquid (5.9 g, 93%). ^1^H NMR (300 MHz, CDCl_3_) δ = 7.52–7.50 (m, 2H), 7.13–7.04 (m, 2H), 3.39 (m, 4H), 1.25–1.17 (m, 6H) ppm. ^13^C{^1^H} NMR (75 MHz, CDCl_3_) δ: 153.7, 151.8, 134.2, 131.0, 130.5, 121.4, 93.4, 42.4, 42.0, 14.3, 13.4 ppm. HRMS (ESI) *m*/*z*: [M + H]^+^ calcd for C_11_H_15_NO_2_I^+^: 320.0142; found 320.0149.

#### Synthesis of 2,3-Diiodophenyl Diethylcarbamate (**4**)

Diisopropyl-amine (3.26 mL, 23.2 mmol, 1.1 equiv) was dissolved in THF (130 mL) under a N_2_ atmosphere at 0 °C (ice bath). *n*-BuLi (9.3 mL, 23.2 mmol, 1.1 equiv, 2.5 M solution in hexanes) was added slowly, and the reaction mixture was stirred for 10 min at 0 °C. The resulting LDA solution was cooled to −78 °C (dry ice/acetone), and compound **3** (6.74 g, 21.1 mmol, 1.0 equiv) in abs. THF (10 mL) was added dropwise over 15 min. The reaction mixture was stirred for another 60 min; then, I_2_ (5.9 g, 23.2 mmol, 1.1 equiv) in THF (10 mL) was added. The reaction was allowed to warm to room temperature in 2 h; then, it was quenched with water. THF was removed under reduced pressure, EtOAc and water were added to the residue, and the organic phase was separated. The aqueous layer was washed with EtOAc, and the combined organic layer was washed with 10% HCl, water, and brine. The organic layer was dried over MgSO_4_ and concentrated in vacuo. The crude product was further purified by flash chromatography (SiO_2_, hexane/EtOAc 5:1) to obtain the product as a yellow oil that solidified overnight as a pale-yellow solid (6.5 g, 69%). ^1^H NMR (300 MHz, CDCl_3_) δ = 7.70 (dd, *J* = 7.0, 2.0 Hz, 2H), 7.12–7.04 (m, 2H), 3.50 (q, *J* = 7.0 Hz, 2H), 3.38 (q, *J* = 7.0 Hz, 2H), 1.30 (t, *J* = 7.1 Hz, 3H), 1.21 (t, *J* = 7.0 Hz, 3H) ppm. ^13^C{^1^H} NMR (75 MHz, CDCl_3_) δ = 152.6, 152.5, 136.2, 130.4, 122.4, 108.7, 106.0, 42.4, 42.1, 14.5, 13.4 ppm. HRMS (ESI) *m*/*z*: [M + H]^+^ calcd for C_11_H_14_NO_2_I_2_^+^: 445.9108; found 445.9124.

#### Synthesis of 2,3-Bis(2,5-dimethylthiophen-3-yl)phenyl Diethylcarbamate (**6**)

A scintillation vial was charged with compound **4** (500 mg, 1.12 mmol, 1.0 equiv), K_2_CO_3_ (776 mg, 5.62 mmol, 5.0 equiv), boronic acid **5**(23) (876 mg, 5.62 mmol, 5.0 equiv), and Pd(PPh_3_)_2_Cl_2_ (39 mg, 0.05 mmol, 0.05 equiv). The vial was purged thoroughly with N_2_; then, EtOH (10 mL, degassed) was added, and the reaction mixture was stirred at 90 °C (in an aluminum heating block) for 16 h. After the reaction was completed, the mixture was cooled to room temperature, diluted with EtOAc, and filtered through a pad of Celite. The solvent was removed under vacuum, and the dark-colored residue was dissolved in EtOAc, washed with water and brine, and dried over MgSO_4_. The crude product was further purified by column chromatography (SiO_2_, hexane/EtOAc (4%)) on a long column to obtain the pure product as a pale-yellow oil that solidified in the freezer (334 mg, 71%). ^1^H NMR (500 MHz, CD_2_Cl_2_) δ = 7.36 (t, *J* = 7.9 Hz, 1H), 7.15 (ddd, *J* = 8.0, 5.7, 1.3 Hz, 2H), 6.39 (s, 1H), 6.19 (s, 1H), 3.24–3.13 (m, 4H), 2.33 (s, 3H), 2.28 (s, 3H), 2.18 (s, 3H), 1.88 (s, 3H), 1.06–0.96 (m, 6H) ppm. ^13^C{^1^H} NMR (126 MHz, CD_2_Cl_2_) δ = 154.5, 150.9, 139.3, 137.9, 134.9, 134.8, 134.5, 133.1, 132.9, 130.9, 128.5, 128.3, 128.1, 128.0, 122.3, 42.5, 42.2, 15.32, 15.27, 14.2, 14.0, 13.8, 13.6 ppm. HRMS (ESI) *m*/*z*: [M + H]^+^ calcd for C_23_H_28_NO_2_S_2_^+^: 414.1555; found 414.1576.

#### Synthesis of 3,4-Bis(2,5-dimethylthiophen-3-yl)-2-hydroxybenzaldehyde (**7**)

Compound **6** (827 mg, 2.00 mmol, 1.0 equiv) was dissolved in abs. THF (18 mL) under a N_2_ atmosphere and cooled to −78 °C (dry ice/acetone). *tert*-BuLi (2.63 mL, 5.00 mmol, 2.5 equiv, 1.9 M solution in pentane) was added dropwise to the solution. The resulting brown solution was stirred for 30 min; then, DMF (387 μL, 5.00 mmol, 2.5 equiv) was added, and the reaction mixture was allowed to warm to rt overnight. Subsequently, water was added, and the mixture was extracted with EtOAc. The organic layer was washed with water and brine, dried over MgSO_4_, and concentrated under vacuum. The crude product was further purified by column chromatography to obtain the pure product as a pale-yellow oil, which solidified in the freezer (506 mg, 74%). ^1^H NMR (500 MHz, CDCl_3_) δ = 11.48 (s, 1H), 9.93 (s, 1H), 7.54 (d, *J* = 8.1 Hz, 1H), 7.00 (d, *J* = 8.0 Hz, 1H), 6.35 (s, 1H), 6.18 (s, 1H), 2.37 (s, 3H), 2.32 (s, 3H), 2.16 (s, 3H), 2.00 (s, 3H) ppm. ^13^C{^1^H} NMR (126 MHz, CDCl_3_) δ = 196.2, 160.1, 146.3, 136.9, 135.2, 135.03, 134.96, 133.9, 132.2, 130.8, 127.7, 127.2, 125.5, 122.3, 119.4, 15.3, 15.1, 14.0 (2) ppm. HRMS (ESI) *m*/*z*: [M + H]^+^ calcd for C_19_H_19_O_2_S_2_^+^: 343.0821; found 343.0834.

#### Synthesis of Compound **1**-*cis*-Enol-**O**

Compound **7** (100 mg, 0.3 mmol, 1.0 equiv) dissolved in EtOH (2.0 mL) and aniline (132 μL, 1.5 mmol, 5.0 equiv) was added, and the reaction mixture was stirred under reflux conditions (in an aluminum heating block) for 20 h. After the reaction was completed, the solvent was evaporated, and the yellow residue was purified by flash chromatography (SiO_2_, hexane/EtOAc (5%)) to obtain the pure product as a yellow oil that solidified in the freezer (119 mg, 97%). ^1^H NMR (500 MHz, CD_2_Cl_2_) δ = 13.75 (broad s, 1H), 8.74 (s, 1H), 7.46–7.43 (m, 3H), 7.34–7.29 (m, 3H), 6.92 (d, *J* = 7.9 Hz, 1H), 6.41 (s, 1H), 6.23 (s, 1H), 2.38 (s, 3H), 2.31 (s, 3H), 2.18 (s, 3H), 2.01 (s, 3H) ppm. ^13^C{^1^H} NMR (126 MHz, CDCl_3_) δ = 162.1, 159.6, 148.3, 142.2, 137.5, 134.54, 134.53, 134.4, 133.1, 131.9, 130.8, 129.4, 127.9, 127.5, 126.9, 125.0, 121.2, 121.1, 117.8, 15.3, 15.0, 13.92, 13.89 ppm. HRMS (ESI) *m*/*z*: [M + H]^+^ calcd for C_25_H_24_NOS_2_^+^: 418.1293; found 418.1312.

#### Synthesis of Compound **8**

To a solution of **1**-*cis*-enol-**O** (30 mg, 0.072 mmol, 1.0 equiv) in 1,2-dichloroethane (3 mL), NaBH(OAc)_3_ (30.5 mg, 0.144 mmol, 2.0 equiv) was added. The mixture was stirred for 4 h and then quenched with an excess of MeOH. After evaporation of the solvents under reduced pressure, the crude mixture was purified by column chromatography (SiO_2_, hexane/EtOAc (5%)). The product was isolated as a pale-yellow material (6 mg, 20%). ^1^H NMR (500 MHz, CD_2_Cl_2_) δ = 7.25 (d, *J* = 7.8 Hz, 1H), 7.20 (dd, *J* = 8.7, 7.6 Hz, 2H), 6.82 (d, *J* = 7.8 Hz, 1H), 6.76–6.79 (m, 3H), 6.35 (s, 1H), 6.17 (s, 1H), 4.40–4.46 (m, 2H), 2.36 (s, 3H), 2.28 (s, 3H), 2.14 (s, 3H), 2.01 (s, 3H). ^13^C{^1^H} NMR (126 MHz, CD_2_Cl_2_) δ = 153.3, 148.7, 138.2, 138.0, 136.9, 135.9, 134.9, 132.9, 132.1, 129.8 (2), 128.3, 128.2, 128.1, 123.72, 123.67, 122.6, 119.2, 114.7 (2), 46.0, 15.5, 15.3, 14.1, 14.0 ppm. HRMS (ESI) *m*/*z*: [M + H]^+^ calcd for C_25_H_26_NOS_2_^+^: 420.1456; found 420.1455.

#### Synthesis of Compound **9**

3′-Hydroxy-[1,1′:2′,1″-terphenyl]-4′-carbaldehyde (**S2**) (40 mg, 0.15 mmol, 1.0 equiv) and freshly distilled aniline (16 mg, 0.17 mmol, 1.1 equiv) in EtOH (1 mL) were stirred under reflux (in an aluminum heating block) for 22 h upon which a yellow precipitate was formed. The solid material was filtered, and excess of water was added to the filtrate that resulted in further precipitation. The combined solid was washed with a small amount of EtOH to give compound **9** as a yellow solid (35 mg, 69%). ^1^H NMR (500 MHz, CD_2_Cl_2_) δ = 13.80 (s, 1H), 8.78 (s, 1H), 7.51 (d, *J* = 8.0 Hz, 1H), 7.46 (t, *J* = 7.7 Hz, 2H), 7.19–7.35 (m, 11H), 7.15–7.17 (m, 2H), 7.06 ppm (d, *J* = 7.9 Hz, 1H); ^13^C{^1^H} NMR (75 MHz, CD_2_Cl_2_) δ = 163.0, 159.6, 148.7, 146.6, 141.7, 137.0, 132.0, 131.9 (2), 130.3 (2), 130.0 (2), 129.5, 128.2 (2), 128.1 (2), 127.6, 127.4, 127.2, 121.8 (2), 121.6, 118.8 ppm.

#### Synthesis of Compound **10**

To a solution of compound **7** (50 mg, 0.2 mmol, 1.0 equiv) in EtOH (1.0 mL), 4-methoxyaniline (76 mg, 0.6 mmol, 4.0 equiv) was added, and the reaction mixture was stirred under reflux conditions (in an aluminum heating block) for 3 h. After the reaction was completed, the solvent was evaporated and the yellow residue was purified by flash chromatography (hexane/EtOAc 9:1) to obtain the pure product as a yellow solid (65 mg, quant.). ^1^H NMR (500 MHz, CD_2_Cl_2_) δ = 13.90 (s, 1H), 8.72 (s, 1H), 7.41 (d, *J* = 7.9 Hz, 1H), 7.32 (d, *J* = 8.9 Hz, 2H), 6.97 (d, *J* = 8.9 Hz, 2H), 6.90 (d, *J* = 7.9 Hz, 1H), 6.40 (s, 1H), 6.23 (s, 1H), 3.84 (s, 3H), 2.38 (s, 3H), 2.31 (s, 3H), 2.18 (s, 3H), 2.01 (s, 3H) ppm. ^13^C{^1^H} NMR (126 MHz, CD_2_Cl_2_) δ: 160.8, 160.0, 159.6, 142.3, 141.7, 138.2, 135.1, 134.8, 134.7, 133.6, 133.0, 131.3, 128.8, 128.1, 125.3, 122.9, 121.7, 118.7, 115.2, 56.1, 15.5, 15.3, 14.3, 14.2 ppm. HRMS (APCI) *m*/*z*: [M + H]^+^ calcd for C_26_H_26_NO_2_S_2_^+^: 448.1399; found 448.1388.

#### Synthesis of Compound **12**

To a solution of **7** (100 mg, 0.29 mmol) in EtOH (6 mL), 2-aminobenzenethiol (110 mg, 0.88 mmol) and acetic acid (96%, 1 μL) were added, and the resulting solution was stirred at 95 °C (in an aluminum heating block) for 20 h. The reaction mixture was allowed to cool to rt, the solvents were removed under reduced pressure, and the crude product was purified by column chromatography (SiO_2_, hexane/EtOAc (5%)). The product was isolated as a pale-yellow oil that solidified in the freezer (82 mg, 0.18 mmol, 63%). ^1^H NMR (500 MHz, CDCl_3_) δ = 13.03 (s, 1H), 7.90–7.92 (m, 2H), 7.70 (d, *J* = 8.1 Hz, 1H), 7.50 (ddd, *J* = 8.4, 7.3, 1.2 Hz, 1H), 7.41 (ddd, *J* = 8.2, 7.2, 1.2 Hz, 1H), 6.94 (d, *J* = 8.1 Hz, 1H), 6.46 (d, *J* = 1.2 Hz, 1H), 6.20 (d, *J* = 1.2 Hz, 1H), 2.40 (s, 3H), 2.32 (s, 3H), 2.20 (s, 3H), 2.01 (s, 3H) ppm. ^13^C{^1^H} NMR (126 MHz, CDCl_3_) δ = 169.6, 156.4, 151.9, 142.0, 137.4, 134.7, 134.6, 134.5, 133.2, 132.8, 132.2, 128.1, 127.5, 127.0, 126.8, 125.62, 125.60, 122.1, 121.8, 121.6, 115.5, 15.4, 15.1, 14.03, 14.01 ppm. HRMS (APCI) *m*/*z*: [M + H]^+^ calcd for C_25_H_22_NOS_3_^+^: 448.0863; found 448.0857.

#### Synthesis of Compound **13**

To a solution of **7** (50 mg, 0.2 mmol, 1.0 equiv) in EtOH (1.0 mL), 4-nitroaniline (61 mg, 0.4 mmol, 3.0 equiv) was added, and the reaction mixture was stirred under reflux conditions (in an aluminum heating block) for 20 h. The reaction mixture was cooled to rt, and the orange-colored precipitate was filtered to obtain the pure product as an orange-yellow solid (48 mg, 71%). ^1^H NMR (500 MHz, CD_2_Cl_2_) δ = 13.13 (s, 1H), 8.75 (s, 1H), 8.30 (d, *J* = 8.9 Hz, 2H), 7.47 (d, *J* = 8.0 Hz, 1H), 7.41 (d, *J* = 8.9 Hz, 2H), 6.97 (d, *J* = 7.9 Hz, 1H), 6.40 (d, *J* = 1.2 Hz, 1H), 6.23 (d, *J* = 1.3 Hz, 1H), 2.37 (s, 3H), 2.31 (s, 3H), 2.18 (s, 3H), 2.01 (s, 3H) ppm. ^13^C{^1^H} NMR (126 MHz, CD_2_Cl_2_) δ = 165.8, 160.4, 154.6, 146.7, 144.2, 137.9, 135.4, 135.1, 135.0, 134.0, 132.5, 132.3, 128.6, 127.9, 125.7, 125.7, 122.5, 122.3, 118.1, 15.4, 15.3, 14.3, 14.2 ppm. HRMS (ESI) *m*/*z*: [M – H]^−^ calcd for C_25_H_21_N_2_O_3_S_2_^–^: 461.0999; found 461.0971.

## Data Availability

The data underlying this study are available in the published article and its Supporting Information.

## Supporting Information Available

Synthesis of intermediates **S1** and **S2**; X-ray crystallographic analysis of **1***-cis-*enol*-***O** and **9**; further UV–vis spectroscopic characterizations; simulation of photoabsorption spectra; ^1^H NMR spectroscopic analysis of **1**-*cis*-keto-**C**; NMR spectra of reported compounds; and Cartesian coordinates of optimized geometries of **1** (PDF)
