# Supplementary material for: Salicylideneaniline/Dithienylethene Hybrid Molecular Switches: Design, Synthesis, and Photochromism
Source: J Org Chem. 2023 Dec 7;89(1):16–26. doi: 10.1021/acs.joc.3c00828 (PMC10777402; doi:10.1021/acs.joc.3c00828)
Supplement: Supplementary file 1 — jo3c00828_si_001.pdf [file jo3c00828_si_001.pdf]

Supporting Information

Salicylideneaniline/dithienylethene hybrid molecular switches: design, synthesis, and photochromism

Péter Pál Kalapos,[a] Attila Kunfi,[a] Marcell M. Bogner,[a] Tamás Holczbauer,[b] Michał Andrzej Kochman,*[c] Bo Durbeej,*[d] Gábor London*[a]

[a] MTA TTK Lendület Functional Organic Materials Research Group, Institute of Organic Chemistry, Research Centre for Natural Sciences, 1117 Budapest, Magyar tudósok krt. 2, Hungary

[b] Institute of Organic Chemistry, Centre for Structural Science, Research Centre for Natural Sciences, 1117 Budapest, Magyar tudósok krt. 2, Hungary

[c] Institute of Physical Chemistry, Polish Academy of Sciences, Marcina Kasprzaka 44/52, 01-224 Warsaw, Poland

[d] Division of Theoretical Chemistry, IFM, Linköping University, SE-58183 Linköping, Sweden

Emails: mkochman@ichf.edu.pl (M.A.K); bodur@ifm.liu.se (B.D.); london.gabor@ttk.hu (G.L.)

Table of Contents

S1 Synthesis of intermediates **S1** and **S2** S3

S2 X-Ray crystallography S4

S3 Additional UV-vis spectroscopic characterizations S8

S4 Simulation of photoabsorption spectra S12

S4.1 Isomerism in compound **1** S12

S4.2 Simulation of photoabsorption spectra S16

S5 Additional ^{1}H NMR spectroscopic characterizations S21

S6 NMR spectra S26

S7 References S38

S8 Cartesian coordinates (in Å) of optimized geometries of compound **1** S41

S1 Synthesis of intermediates S1 and S2

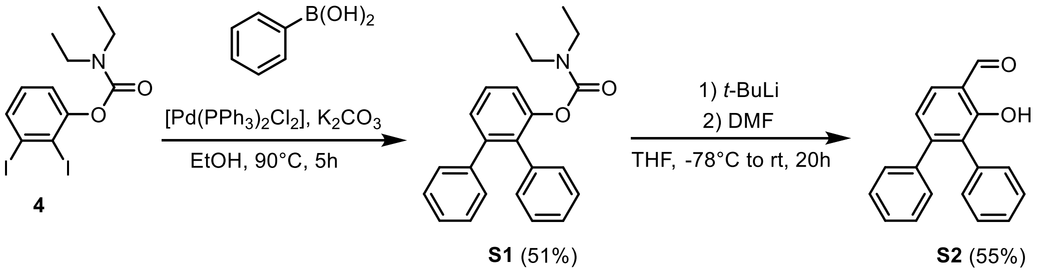

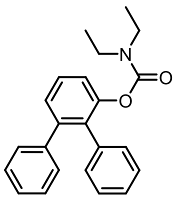

[1,1':2',1''-terphenyl]-3'-yl diethylcarbamate (**S1**) A mixture of compound **4** (500 mg, 1.12 mmol, 1.0 eq.), phenylboronic acid (548 mg, 4.49 mmol, 4.0 eq.), $Pd(PPh_3)_2Cl_2$ (39.4 mg, 0.056 mmol, 5 mol%) and K_2CO_3 (466 mg, 3.37 mmol) in EtOH (10 mL) was stirred at 90 °C for 5 h. The reaction was allowed to cool to rt, diluted with EtOAc and filtered over a pad of celite. The solvent was evaporated under reduced pressure and the crude product was purified by column chromatography (SiO_2, hexane/EtOAc 5: 1) to obtain **S1** (199 mg, 51%). ^{1}H NMR (300 MHz, $CDCl_3$) δ = 7.47 (t, *J* = 7.8 Hz, 1H), 7.34 (d, *J* = 7.6 Hz, 1H), 7.29 (d, *J* = 8.0 Hz, 1H), 7.12 – 2.22 (m, 11H), 3.27 (q, *J* = 7.2 Hz, 2H), 3.09 (q, *J* = 7.3 Hz, 2H), 1.07 (t, *J* = 7.0 Hz, 3H), 0.88 ppm (t, *J* = 7.2 Hz, 3H). ^{13}C NMR (75 MHz, $CDCl_3$) δ = 154.13, 149.27, 142.76, 140.80, 136.46, 134.10, 130.56 (2), 129.78 (2), 128.04, 127.58 (2), 127.50 (2), 127.26, 126.61, 126.41, 122.16, 41.93, 41.51, 13.58, 13.13 ppm.

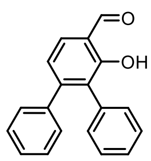

3'-hydroxy-[1,1':2',1''-terphenyl]-4'-carbaldehyde (S2) Under a N_2 atmosphere compound **S2** (200 mg, 0.58 mmol) in THF (5 mL) was cooled to -78 °C. To this yellow solution, *tert*-BuLi (0.61 mL, 1.16 mmol, 1.9 M solution in pentane) was added dropwise. The resulting deep red solution was stirred at -78°C for 30 min followed by the addition of DMF. The mixture was stirred overnight without further cooling, followed by the addition of water. The aqueous mixture was extracted with EtOAc (3x), and the combined organic layer was washed with brine and dried ($MgSO_4$). The solvent was removed under reduced pressure and the crude product was purified by column chromatography (SiO_2, hexane/EtOAc 12 : 1) to obtain **S2** (87 mg, 55%). ^{1}H NMR (300 MHz, CD_2Cl_2) δ = 11.47 (s, 1H), 9.98 (s, 1H), 7.66 (d, *J* = 8.0 Hz, 1H), 7.11 – 7.29 ppm (m, 11H); ^{13}C NMR (75 MHz, CD_2Cl_2) δ = 197.14, 160.03, 150.40, 140.96, 135.69, 133.31, 131.70 (2), 130.11 (2), 129.87, 128.35 (2), 128.26 (2), 127.85, 127.53, 122.57, 120.17 ppm. HRMS (ESI) m/z: $[M – H]^-$ calcd for $C_{19}H_{13}O_2^-$: 273.0916; found 273.0896.

S2 X-Ray crystallography

*Crystal data of **1**-cis-enol-**O**:* Obtained from *n*-hexane/MeOH solvent system (liquid-liquid diffusion). $C_{25}H_{23}NOS_2$, *Fwt.*: 417.56, yellow, needle, size: 0.50 x 0.18 x 0.10 mm, monoclinic, space group $P\ 2_1/n$, a = 9.0387(10)Å, b = 18.892(2)Å, c = 13.3969(13)Å, α = 90°, β = 106.433(7)°, γ = 90°, V = 2194.3(4)Å^3, T = 161(2)K, Z= 4, Z'=1, F(000) = 880, D_x = 1.264 Mg/m^3, μ 0.258mm^{-1}.

A crystal of **1**-*cis*-enol-**O** was mounted on a glass fiber. Cell parameters were determined by least-squares using 36453 (3.17 ≤ θ ≤ 27.455°) reflections.

Intensity data were collected on a Rigaku RAXIS-RAPID II diffractometer (monochromator; Mo-$K\alpha$ radiation, λ = 0.71075Å) at 161(2) K in the range 3.171 ≤ θ ≤ 23.534. A total of 57794 reflections were collected of which 3253 were unique [R(int) = 0.2097, $R(\sigma)$ = 0.0731]; intensities of 2394 reflections were greater than 2σ(I). Completeness to θ = 0.998.

A numerical absorption correction was applied to the data (the minimum and maximum transmission factors were 0.992150 and 0.997759).

The structure was solved by direct methods (and subsequent difference syntheses).

Anisotropic full-matrix least-squares refinement on F^2 for all non-hydrogen atoms yielded R_1 = 0.0794 and wR^2 = 0.1417 for 1332 [I>2σ(I)] and R_1 = 0.1153 and wR^2 = 0.1547 for all (3253) intensity data, (number of parameters = 267, goodness-of-fit = 1.153, the maximum and mean shift/esd is 0.000 and 0.000).

The maximum and minimum residual electron density in the final difference map was 0.26 and -0.23e.Å^{-3}.

The weighting scheme applied was $w = 1/[\sigma^2(F_o^2)+(0.04902.0559P)^2+2.0559P]$ where $P = (F_o^2+2F_c^2)/3$.

*Crystal data of **9**:* Obtained from *n*-hexane/MeOH solvent system (liquid-liquid diffusion). $C_{25}H_{19}NO$, *Fwt.*: 349.41, yellow, bladed, size: 0.45 x 0.25 x 0.05 mm, monoclinic, space group $P\ 2_1/c$, a = 13.8168(12)Å, b = 9.2586(8)Å, c = 15.0816(13)Å, α = 90°, β = 108.458(8)°, γ = 90°, V = 1830.1(3)Å^3, T = 143(2)K, Z= 4, Z'=1, F(000) = 736, D_x = 1.268 Mg/m^3, μ 0.598mm^{-1}.

A crystal of **9** was mounted on a glass fiber. Cell parameters were determined by least-squares using 10181 (3.09 ≤ θ ≤ 68.265°) reflections.

Intensity data were collected on a(n) Rigaku RAXIS-RAPID II diffractometer

(monochromator; Cu-$K\alpha$ radiation, $\lambda = 1.54187$Å) at 143(2) K in the range $3.372 \leq \theta \leq 68.227$. A total of 16316 reflections were collected of which 3278 were unique [R(int) = 0.0501, $R(\sigma)$ = 0.0450]; intensities of 2848 reflections were greater than $2\sigma(I)$. Completeness to θ = 0.979.
A numerical absorption correction was applied to the data (the minimum and maximum transmission factors were 0.948877 and 0.987468).
The structure was solved by direct methods (and subsequent difference syntheses).
Anisotropic full-matrix least-squares refinement on F^2 for all non-hydrogen atoms yielded R_1 = 0.0556 and wR^2 = 0.1071 for 1332 [I>$2\sigma(I)$] and R_1 = 0.0685 and wR^2 = 0.1127 for all (3278) intensity data, (number of parameters = 249, goodness-of-fit = 1.116, the maximum and mean shift/esd is 0.000 and 0.000).
The maximum and minimum residual electron density in the final difference map was 0.18 and -0.17e.Å^{-3}.
The weighting scheme applied was $w = 1/[\sigma^2(F_o^2)+(0.02590.9592P)^2+0.9592P]$ where $P = (F_o^2+2F_c^2)/3$.

Crystal Clear[1] (developed by Rigaku Company) software were used for data collection and refinement. Numerical absorption corrections[2] were applied to the data. The structures were solved by direct methods. Anisotropic full-matrix least-squares refinements were performed on F^2 for all non-hydrogen atoms. Hydrogen atoms bonded to C atoms were placed in calculated positions and refined in a riding-model approximation. The computer programs used for the structure solution, refinement and analysis of the structures were Shelx[2,3,4], Wingx[5], Platon[6] and Olex[2,7]. Program Mercury[8] was used for the graphical representation. Details of crystallographic data, data collection and refinement for crystal crystals **1**-*cis*-enol-**O** and **9** are collected in Table S1.

CCDC 2235162-2235163 contains the supplementary crystallographic data for this paper. These data can be obtained free of charge from The Cambridge Crystallographic Data Centre via www.ccdc.cam.ac.uk/structures.

Table S1. Summary of crystallographic data, data collections, structure determination and refinement for **1**-*cis*-enol-**O** and **9**.

Structure	**1**-*cis*-enol-**O**	**9**
CCDC number	2235162	2235163
Empirical formula	$C_{25}H_{23}NOS_2$	$C_{25}H_{19}NO$
Formula weight	417.56	349.41
Temperature (K)	161(2)	143(2)
Radiation and wavelength	Mo-Kα, λ =0.71075Å	Cu-Kα, λ =1.54187Å
Crystal system	monoclinic	monoclinic
Space group	$P\,2_1/n$	$P\,2_1/c$
Unit cell dimensions		
a (Å)	9.0387(10)	13.8168(12)
b (Å)	18.892(2)	9.2586(8)
c (Å)	13.3969(13)	15.0816(13)
β (°)	106.433(7)	108.458(8)
Volume (Å^3)	2194(1)	1830(1)
Z, Z'	4, 1	4, 1
Density (calculated) (Mg/m^3)	1.264	1.268
Absorption coefficient, μ (mm^{-1})	0.258	0.598
F(000)	880	736
Crystal colour, description	yellow, needle	yellow, bladed
Crystal size (mm)	0.50 x 0.18 x 0.10	0.45 x 0.25 x 0.05
Absorption correction	numerical	numerical
Max. and min. transmission	0.992150 and 0.997759	0.948877 and 0.987468
θ–range for data collection	$3.171 \leq \theta \leq 23.534°$	$3.372 \leq \theta \leq 68.227°$
Index ranges	$-10 \leq h \leq 10$; $-21 \leq k \leq 21$; $-15 \leq l \leq 15$	$-16 \leq h \leq 16$; $-10 \leq k \leq 11$; $-17 \leq l \leq 17$
Reflections collected	57794	16316
Completeness to 2θ	0.998	0.981
Independent reflections	3253 [*R*(int) =0.2097]	3278 [*R*(int) =0.0501]
Reflections $I>2\sigma(I)$	2394	2848
Data / restraints / parameters	3253 /0 /267	3278 /0 /249
Goodness-of-fit on $F2$	1.153	1.116
Final *R* indices [$I>2\sigma(I)$]	R_1 =0.0794, wR^2 =0.1417	R_1 =0.0556, wR^2 =0.1071
R indices (all data)	R_1 =0.1153, wR^2 =0.1547	R_1 =0.0685, wR^2 =0.1127
Max. and mean shift/esd	0.000;0.000	0.000;0.000
Largest diff. peak and hole (e.Å^{-3})	0.26 and -0.23 e.Å^{-3}	0.18 and-0.17

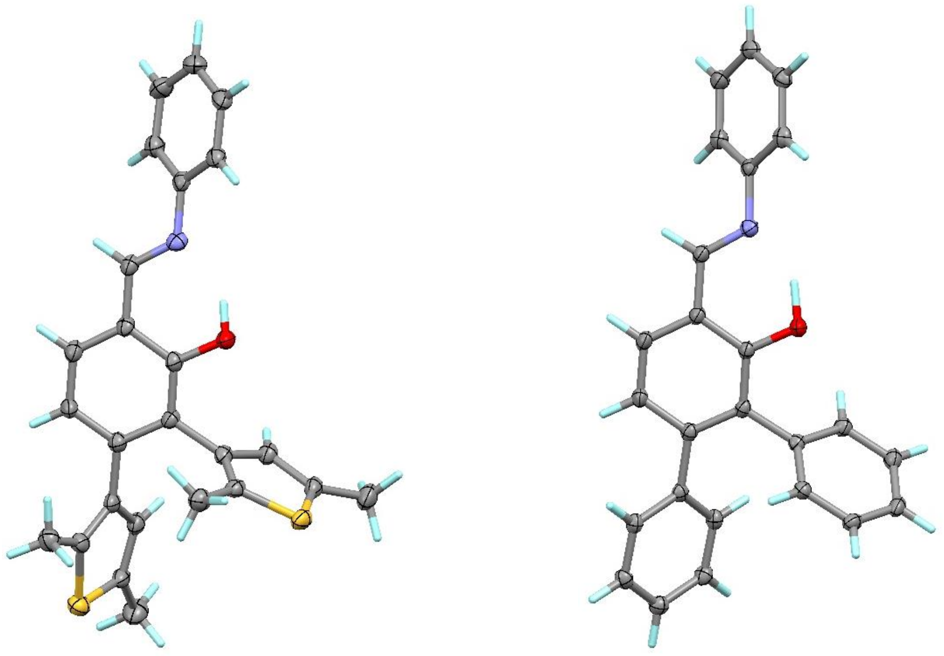

Figure S1. ORTEP style diagrams of the asymmetric units in the crystals of **1-***cis*-enol-**O** (left) and **9** (right). Representations are drawn at the 50% probability level.

S3 Additional UV-vis spectroscopic characterizations

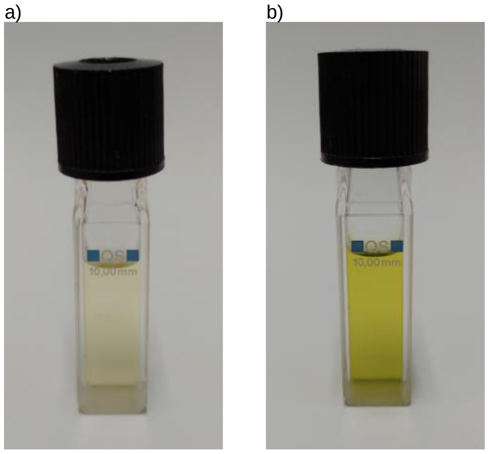

Figure S2. Colour change of the solution of **1**-*cis*-enol-**O** in MeOH (a) upon irradiation with 365 nm light (b).

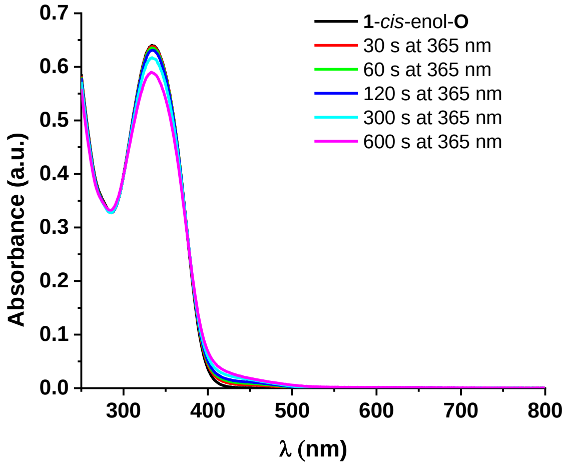

Figure S3. Irradiation of **1**-*cis*-enol-**O** in MeCN at rt.

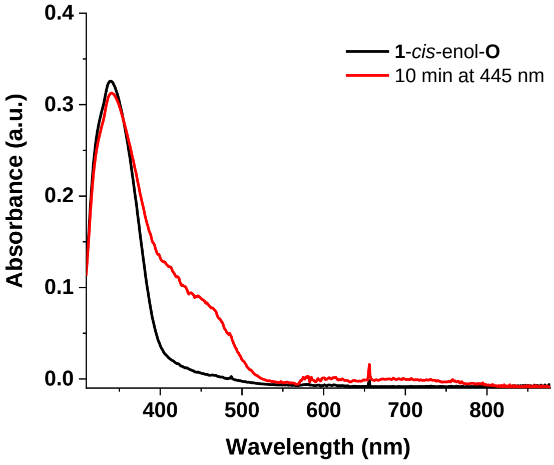

Figure S4. Irradiation of **1**-*cis*-enol-**O** in MeOH with 445 nm light at rt.

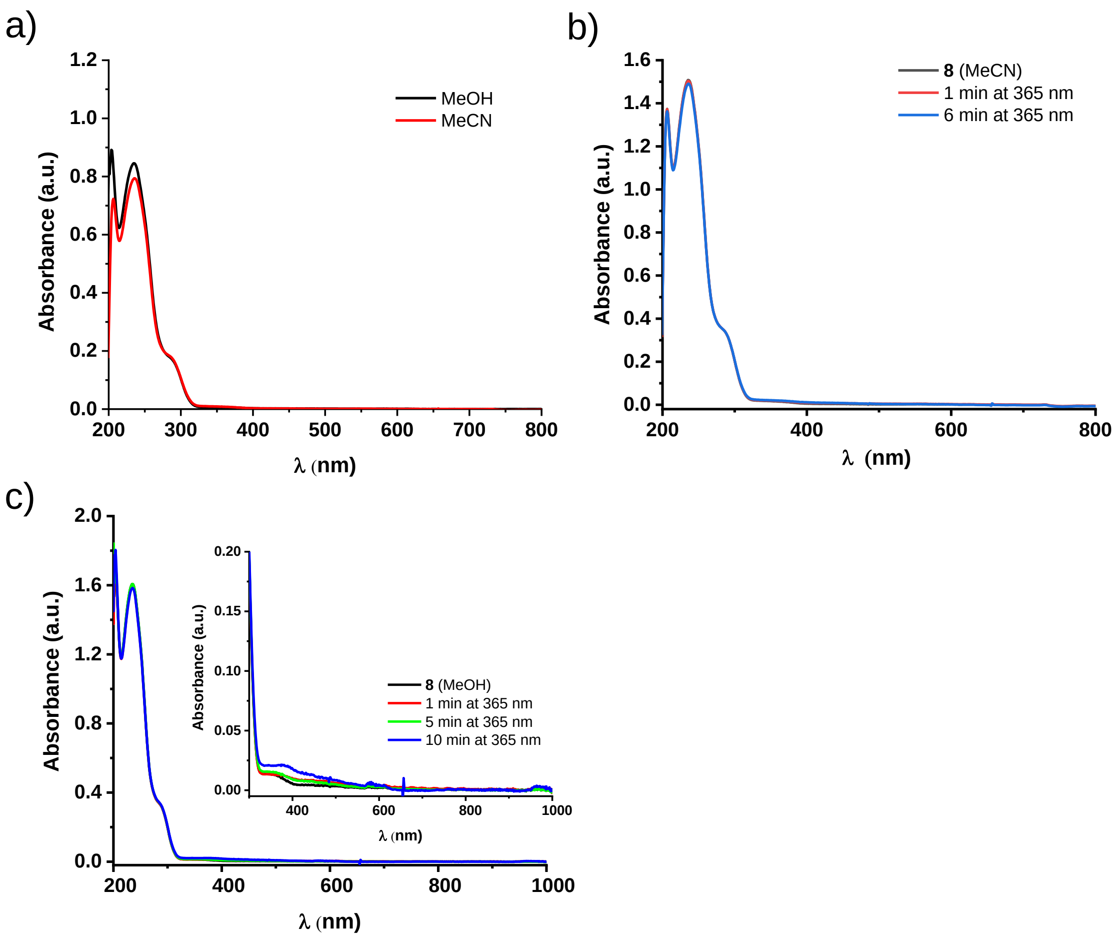

Figure S5. UV-vis spectra of compound **8** in MeOH and MeCN (a) and irradiation with 365 nm light in MeCN (b) and MeOH (c) at rt.

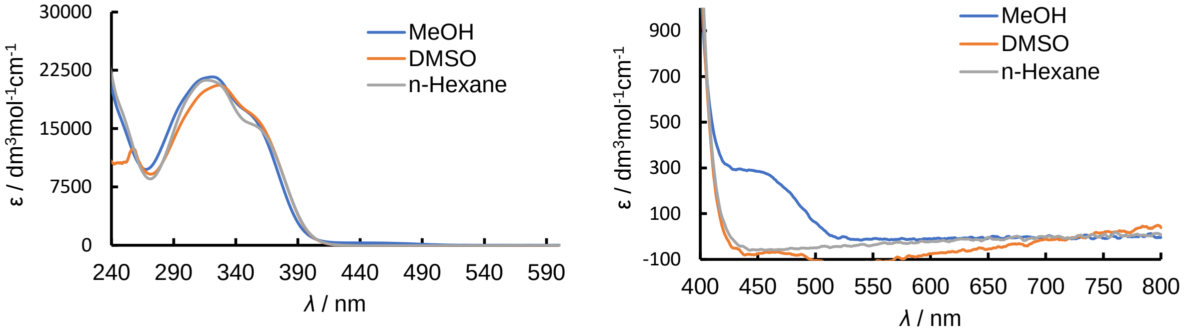

Figure S6. UV-vis spectra of compound **9** in different solvents at rt. No change was observed upon irradiation with 365 nm light at rt.

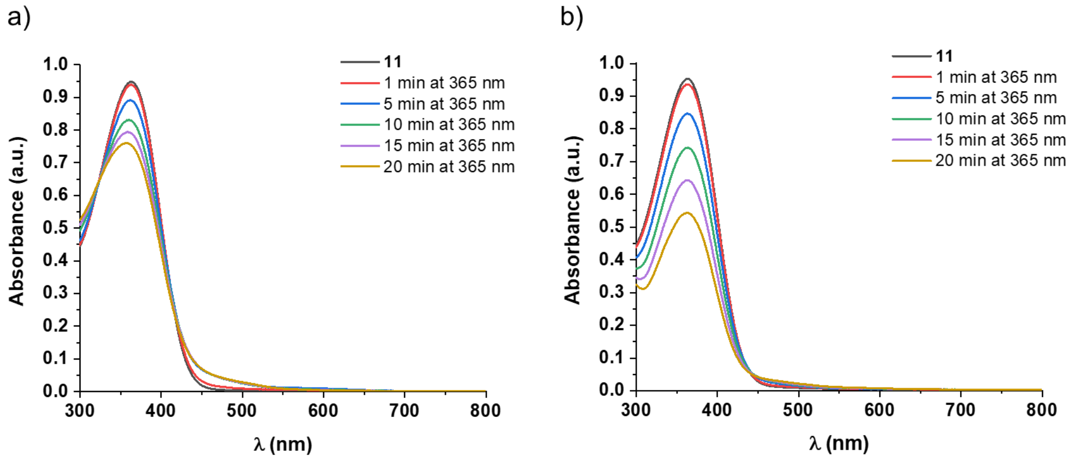

Figure S7. UV-vis spectra of compound **11** in (a) MeCN and (b) in MeOH and the effect of subsequent irradiation with 365 nm light (rt) on the spectra.

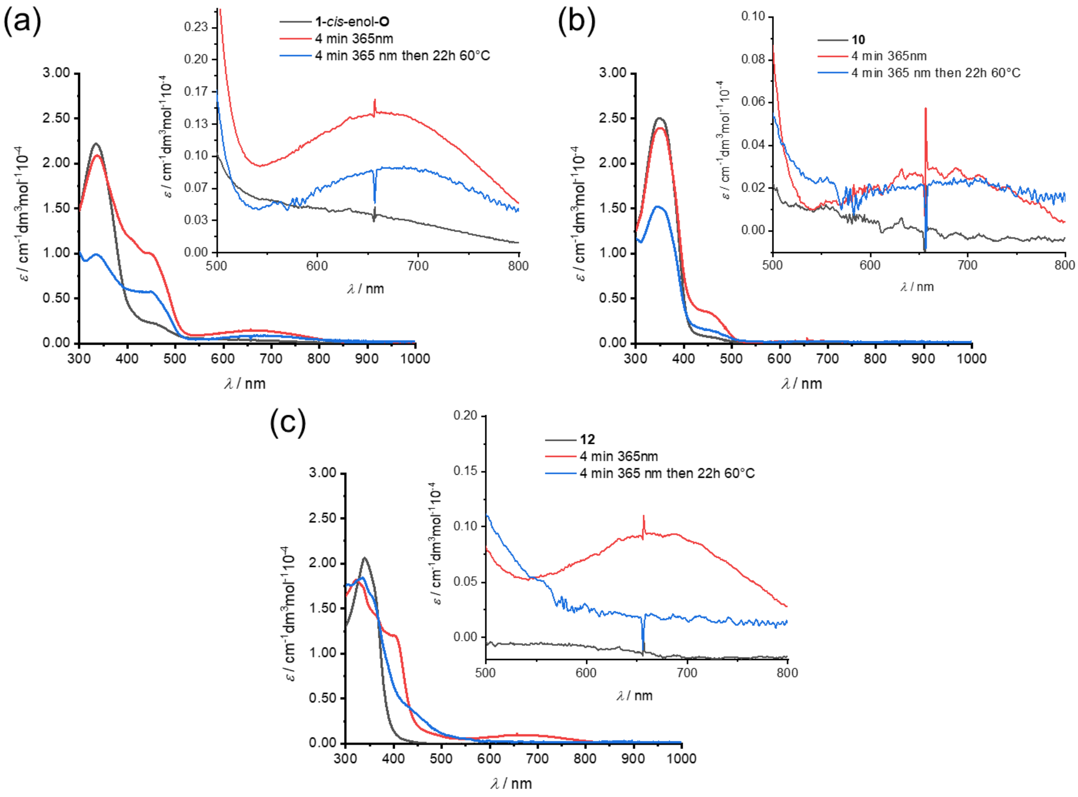

Figure S8. Thermal reversibility of the photoproducts of compounds **1**-*cis*-enol-**O** (a), **10** (b) and **12** (c) studied by UV-vis spectroscopy.

S4 Simulation of photoabsorption spectra

S4.1 Isomerism in compound 1

In order to facilitate the interpretation of the experimentally-observed photoabsorption spectra of compound **1** and its photoproducts, we simulated these spectra with the use of electronic structure calculations. When interpreting the spectra, it is necessary to take into account the fact that compound **1** possesses a large number of isomers, some of which are in chemical equilibria with each other. Our first order of business will therefore be to identify the relevant isomers and the equilibria between them.

As shown schematically in Figure S9, the salicylideneaniline (SA) moiety within compound **1** can exist in four isomeric forms, which differ in the tautomeric form (enol or keto) and in the configuration around the C1–C7 bond (*cis* or *trans*). The *cis*-enol and the *trans*-enol isomers can interconvert thermally via a rotation around the C1–C7 bond, which is formally a single bond in these isomers. Furthermore, the *cis*-enol and *cis*-keto isomers can interconvert thermally via intramolecular proton transfer.

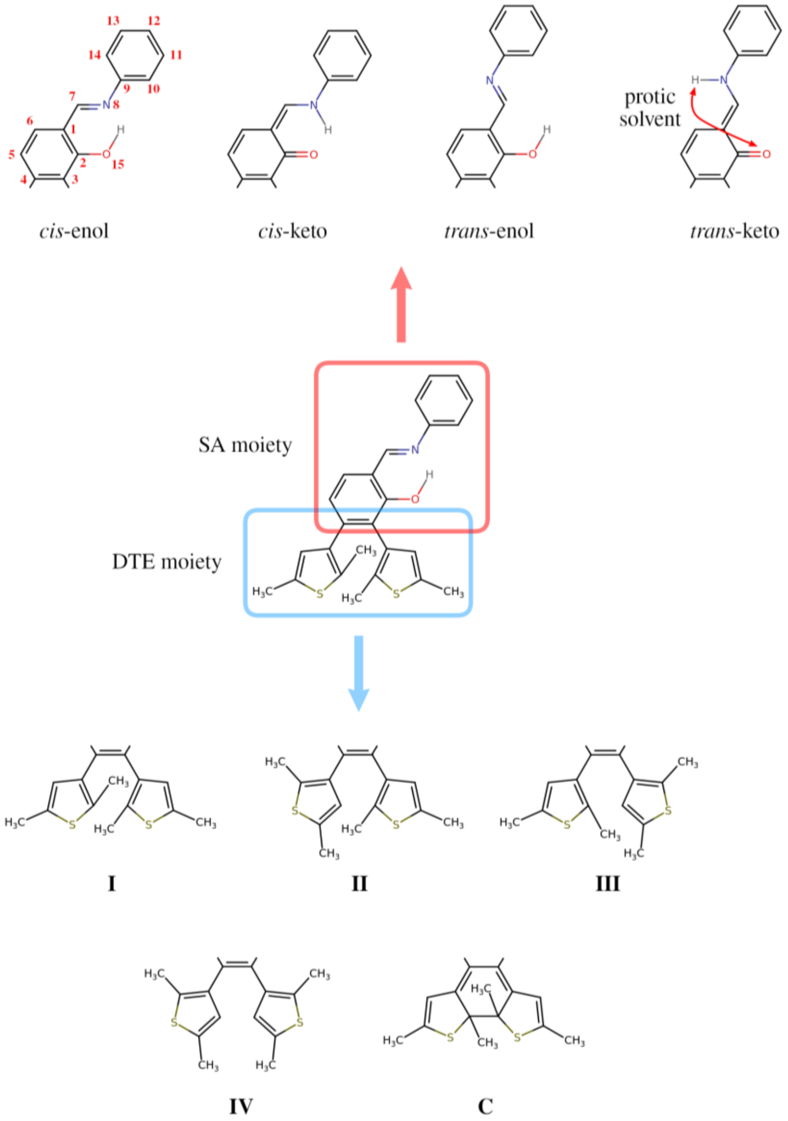

Figure S9. A catalogue of the relevant isomers of compound **1**. The numbering of atoms comprising the SA moiety is given in red.

The *trans*-keto isomer is special in that its tautomerization reaction may depend on the nature of the solvent. In an aprotic solvent, *trans*-keto to *trans*-enol tautomerization cannot occur because atoms N8 and O15 are distant from one another. On the other hand, in a protic solvent such as an alcohol, it becomes possible for the *trans*-keto and the *trans*-enol isomers to interconvert thermally by a proton transfer reaction that is mediated by the solvent. (In Figure S9, this process is indicated with a red double-headed arrow.) Hence, in a protic solvent, all four isomeric forms of the SA moiety are in chemical equilibrium with one another. In what follows, when calculating the populations of the various isomers of compound **1**, we assume that the sample has been dissolved in a protic solvent, and that the four isomeric forms are in equilibrium.

The dithienylethene (DTE) moiety, in turn, can exist in five isomeric forms: four open-ring conformations, which we label **I** to **IV**, and a closed-ring configuration, which we denote **C**. Because the four open-ring conformations only differ in the orientation of the thienyl groups, they are expected to be in chemical equilibrium with one another. The closed-ring structure cannot be formed thermally; it can only be formed as a photoproduct following the irradiation of compound **1**.

In what follows, the overall isomeric form of the molecule is specified by a three-part label such as ‘‘*cis*-enol-**II**,’’ which lists the configuration around the C1–C7 bond in the SA moiety, the tautomeric form of the SA moiety, and the isomeric form of the DTE moiety, respectively.

In summary, a total of 4 × 5 = 20 isomers may potentially be relevant for the photophysics of compound **1**. Our next goal will be to estimate the relative abundances of the various isomers before and after the irradiation of the sample. To this end, we performed geometry optimizations with the use of density functional theory (DFT). These calculations were performed with the computational chemistry software Gaussian 16, Revision A.03.[9] We employed the well-known B3LYP[10-12] functional in combination with the def2-SVP basis set.[13] The calculated energies and gradients were corrected for dispersion effects via the ‘D3’ semiempirical correction scheme of Grimme and coworkers.[14] The default ‘ultrafine’ integration grid was used. All calculations were performed in the gas phase (that is to say, solvent effects were neglected). All optimized geometries were confirmed to correspond to energy minima by an analytic calculation of vibrational modes.

The relative energies and Gibbs free energies of the various isomers are listed in Table S2 below. It can be seen that, among the sixteen open-ring isomers, the four *cis*-enol isomers (*cis*-enol-**I** to *cis*-enol-**IV**) are close in energy to one another, and substantially lower in energy than the other open-ring isomers. As a consequence, at equilibrium, only the four *cis*-enol isomers are populated to a significant extent. The mole fractions of the four isomers prior to irradiation (column x_0 in Table S2) are estimated as *cis*-enol-**I**: 0.06, *cis*-enol-**II**: 0.26, *cis*-enol-**III**: 0.41, and *cis*-enol-**IV**: 0.26. The populations of the remaining open-ring isomers are negligible.

The irradiation of compound **1** triggers a photocyclization reaction of the DTE moiety and possibly also other photoinduced reactions that are characteristic of salicylideneaniline derivatives: excited-state proton transfer (ESIPT) and *cis*-*trans* photoisomerization.[15-17] While the photocyclization of the DTE moiety is thermally irreversible, ESIPT and *cis*-*trans* photoisomerization can be reversed by thermal reactions. It follows that, after irradiation, the four possible closed-ring photoproducts (*cis*-enol-**C**, *cis*-keto-**C**, *trans*-enol-**C**, and *trans*-keto-**C**) will establish a chemical equilibrium with each other. The *cis*-keto-**C** isomer is by far the lowest in energy among the four. Accordingly, the photoproduct mixture will be dominated by that isomer, while the populations of the other three isomers will be negligible (see column x_1 in Table S2).

In summary, based on thermochemical considerations, we conclude that an equilibrium mixture of open-ring isomers is dominated by the four *cis*-enol isomers (*cis*-enol-**I** to *cis*-enol-**IV**). Following irradiation and equilibration between the photoproduct closed-ring isomers, the main final product will be the *cis*-keto-**C** isomer.

Table S2. Energies (E) and Gibbs free energies (G) of isomers of compound **1** in the gas phase, as calculated at the B3LYP-D3/def2-SVP level of theory. x_0 is the estimated mole fraction of the given open-ring isomer at equilibrium (prior to irradiation). x_1 is the estimated mole fraction of the given closed-ring isomer among the closed-ring photoproducts.

Isomer	E (kJ/mol)	G (kJ/mol)	x_0	x_1
cis-enol-**I**	2.3	4.8	0.06	–
cis-enol-**II**	2.1	1.2	0.26	–
cis-enol-**III**	0.0	0.0	0.41	–
cis-enol-**IV**	3.6	1.2	0.26	–
cis-keto-**I**	15.2	15.2	9×10^{-4}	–
cis-keto-**II**	16.4	12.9	2×10^{-3}	–
cis-keto-**III**	13.3	11.2	4×10^{-3}	–
cis-keto-**IV**	18.1	13.0	2×10^{-3}	–
trans-enol-**I**	52.6	52.3	3×10^{-10}	–
trans-enol-**II**	51.8	48.3	1×10^{-9}	–
trans-enol-**III**	50.5	48.0	2×10^{-9}	–
trans-enol-**IV**	53.8	48.7	1×10^{-9}	–
trans-keto-**I**	58.2	56.4	5×10^{-11}	–
trans-keto-**II**	60.2	55.6	7×10^{-11}	–
trans-keto-**III**	57.0	52.9	2×10^{-10}	–
trans-keto-**IV**	62.6	56.1	6×10^{-11}	–
cis-enol-**C**	85.4	94.5	–	2×10^{-5}
cis-keto-**C**	61.0	67.8	–	1.00
trans-enol-**C**	144.4	151.1	–	3×10^{-15}
trans-keto-**C**	88.0	93.0	–	4×10^{-5}

S4.2 Simulation of photoabsorption spectra

Having estimated the composition of a sample of compound **1** in solution prior to irradiation, and the isomeric form of the closed-ring photoproduct, we are now prepared to simulate their photoabsorption spectra. The spectra were simulated with the use of the semiclassical nuclear ensemble method.[18,19] In this method, the spectrum is built as sum of contributions from molecular geometries $\{\mathbf{R}_l\}$ sampled from the Wigner distribution of the ground-state equilibrium geometry. The photoabsorption cross-section σ at photon energy E is given by:

$$\sigma(E) = \frac{\pi e^2 \hbar}{2mc\varepsilon_0 n_r E N_p} \sum_n^{N_{fs}} \sum_l^{N_p} \Delta E_{0,n}(\mathbf{R}_l) f_{0n}(\mathbf{R}_l) g\big(E - \Delta E_{0,n}(\mathbf{R}_l), \delta\big) \quad (1)$$

Here, m and e are the electron charge and mass, c is the speed of light in vacuum, ε_0 is the vacuum permittivity, and n_r is the refractive index of the medium. Furthermore, $\Delta E_{0,n}(\mathbf{R}_l)$ is the vertical excitation energy from the ground state into the n-th excited state calculated at

nuclear geometry $\mathbf{R}_l$, and $f_{0n}(\mathbf{R}_l)$ is the associated oscillator strength. N_{fs} is the number of states included in the simulation, and N_p is the number of molecular geometries included in the calculation. Here, for each isomer, $N_p = 250$ was used. $g\left(E - \Delta E_{0,n}(\mathbf{R}_l), \delta\right)$, finally, is a line-shape function. Specifically, we employed a Gaussian line-shape function:

$$g\left(E - \Delta E_{0,n}(\mathbf{R}_l), \delta\right) = \frac{1}{\sqrt{2\pi(\delta/2)^2}} exp\left(\frac{-\left(E-\Delta E_{0,n}(\mathbf{R}_l)\right)^2}{2(\delta/2)^2}\right) \quad (2)$$

where the parameter δ was set to 0.2 eV.

In the case of the open-ring isomers, the spectrum was calculated as a weighted average of contributions from the individual isomers:

$$\sigma(E) = \sum_K x_{0,K}\sigma_K(E) \quad (3)$$

where $x_{0,K}$ is the mole fraction of the K-th isomer prior to irradiation, and $\sigma_K(E)$ is its photoabsorption cross-section.

The final result was expressed in terms of the extinction coefficient ε, which is related to the photoabsorption cross-section by:

$$\varepsilon = \frac{0.001 N_A \sigma}{ln(10)} \quad (4)$$

Here, it should be noted that equation 4 assumes that σ is expressed in units of cm^2 , and ε in units of L mol^{-1} cm^{-1}.

A total of three photoabsorption spectra were computed: one for an equilibrium mixture of the *cis*-enol isomers (*cis*-enol-**I** to *cis*-enol-**IV**), another for an equilibrium mixture of the *cis*-keto isomers (*cis*-keto-**I** to *cis*-keto-**IV**), and a third for the pure *cis*-keto-**C** isomer. The vertical excitation energies which appear in equation 1 were evaluated with the simplified Tamm-Dancoff approximation (sTDA) as implemented in the program Orca, version 5.0.3.[20] The CAM-B3LYP functional[21] was employed. This choice of functional was motivated by the fact that CAM-B3LYP achieves fairly good accuracy in the calculation of the excitation energies

of organic molecules,[22] and has been specifically recommended for use with the sTDA method.[23] As in the ground-state DFT calculations, the def2-SVP basis set was used. The energy threshold for configuration state functions (CSFs) was set to 15 eV. When calculating the spectrum, transitions with energies higher than 8 eV were discarded. For the sake of simplicity, the refractive index of the medium was set to unity.

The resulting spectra are plotted in Figure S10. Let us examine first the case of an equilibrium mixture of the open-ring *cis*-enol isomers (Figure S10a). According to our DFT calculations, it is these isomers that dominate in a solution of the open-ring form of compound **1**, so that the simulated spectrum can be compared directly to the experimentally-observed spectrum. It can be seen that the simulation does a reasonably good job of reproducing the spectrum of the mixture of open-ring isomers. Experimentally, the first photoabsorption band of the open-ring isomers features a maximum at around 345 nm. In the simulation, this band is slightly blue-shifted, with a maximum at roughly 330 nm, and has a somewhat higher intensity than in the experimentally-measured spectrum. The fact that the simulation overestimates the photoabsorption intensity is partially due to the fact that the refraction index was set to unity, when in fact methanol at room temperature has a refraction index of roughly 1.33.

Regarding, in turn, the open-ring *cis*-keto isomers (Figure S10b), the calculations predict that an equilibrium mixture of these isomers shows a broad and intense photoabsorption band peaking at roughly 390 nm, with a gently sloping shoulder at roughly 450 nm. This result supports our assignment of the experimentally-observed absorption band near 445 nm to the open-ring *cis*-keto isomers.

Lastly, we consider the *cis*-keto-**C** isomer (Figure S10c), which occurs as a photoproduct following the irradiation of compound **1**. According to the calculations, this isomer has a weak absorption band peaking at around 610 nm, and a much stronger band peaking at roughly 420 nm. The positions and the relative intensities of these bands in the simulated spectrum coincide closely with the bands which appear following the irradiation of the open-ring isomers. This close agreement between simulation and experiment confirms that the emergent bands originate from the *cis*-keto-**C** isomer, and, consequently, that compound **1** undergoes photocyclization.

(a) Equilibrium mixture of the open-ring *cis*-enol isomers.

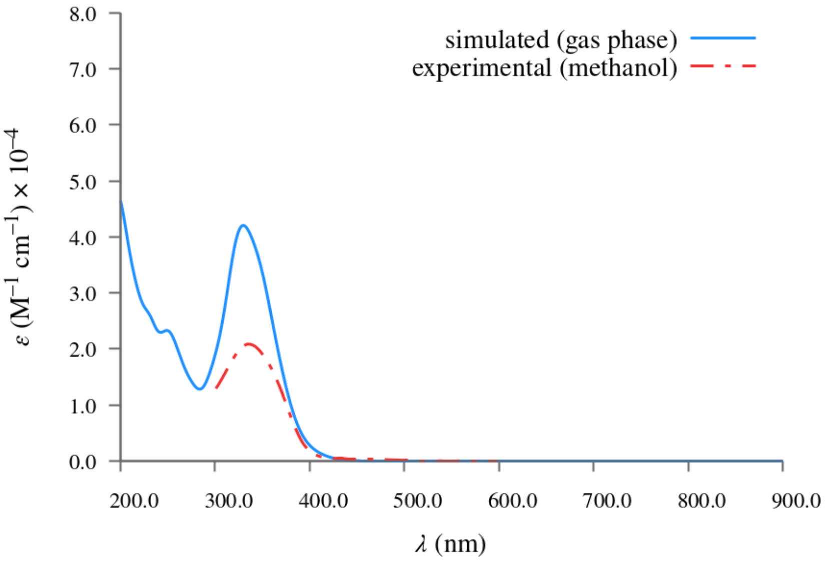

(b) Equilibrium mixture of the open-ring *cis*-keto isomers. The photoabsorption spectrum of these isomers is not known experimentally, as they cannot be isolated from the *cis*-enol isomers, but they are believed to give rise to an absorption band near 445 nm. (See Figure 3 and related discussion in the main body of our paper.)

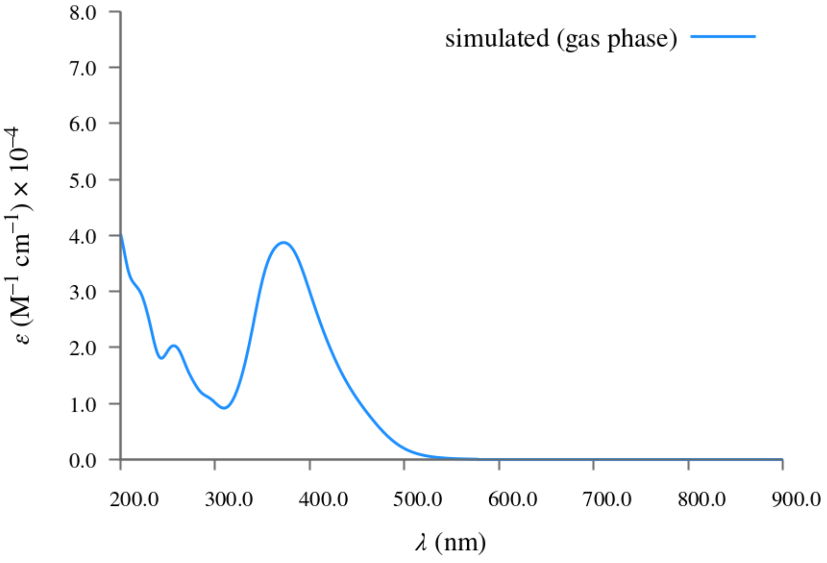

(c) The *cis*-keto-**C** isomer. In this case, the absorption intensity is not known experimentally due to the approximative value of the concentration. For this reason, the experimentally-

measured spectrum was rescaled in such a way that the maximum of the first photoabsorption band has the same intensity as in the simulated spectrum.

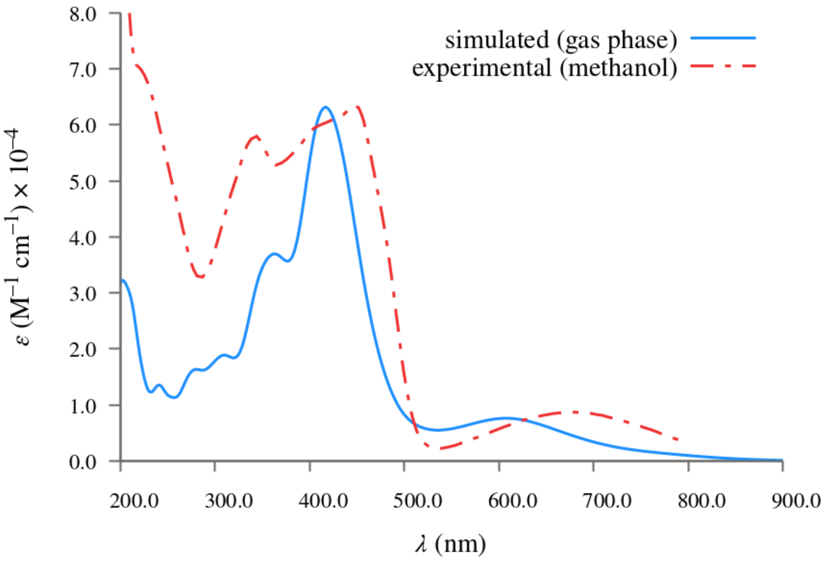

Figure S10. Comparison of the simulated (continuous blue line) and, where available, the experimentally-measured (dashed red line) photoabsorption spectra of isomers of compound **1**.

S5 Additional ^{1}H NMR spectroscopic characterizations

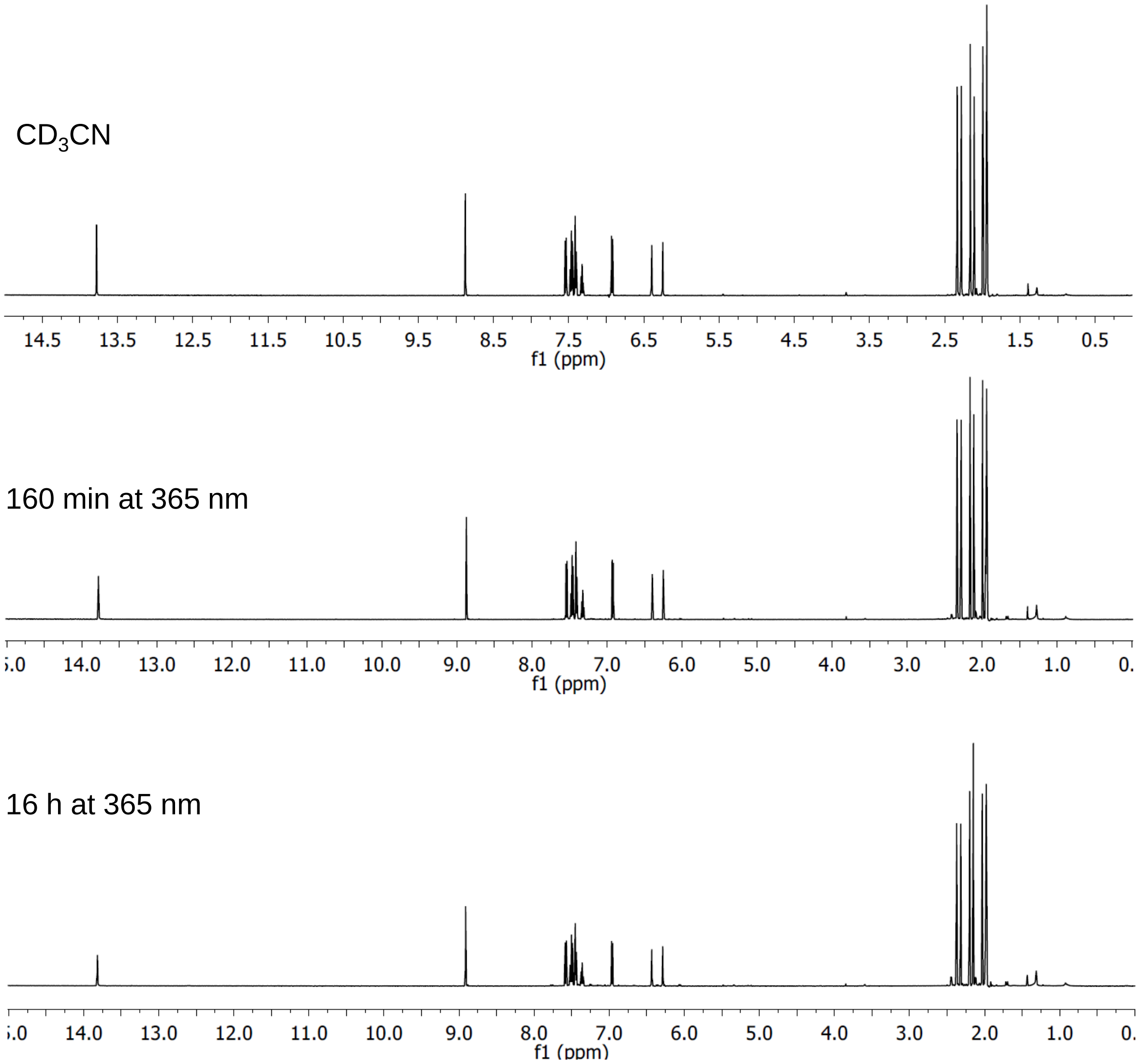

Figure S11. Irradiation of **1**-*cis*-enol-**O** with 365 nm light in CD_3CN at rt followed by ^{1}H NMR spectroscopy (500 MHz).

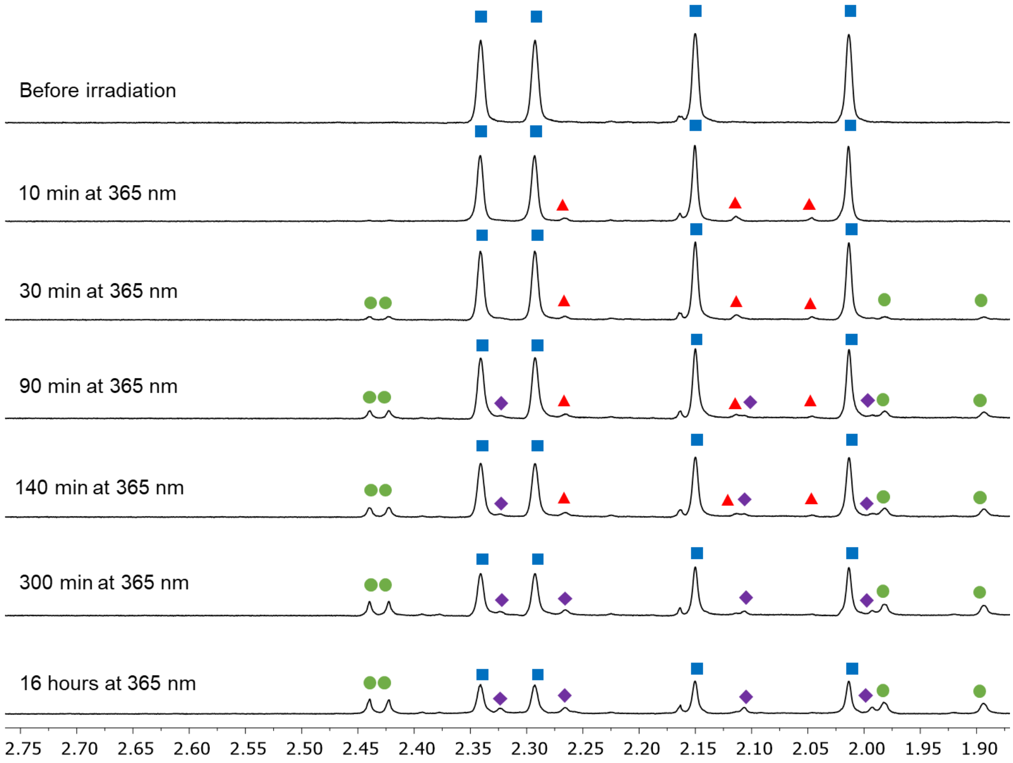

Figure S12. Irradiation of **1**-*cis*-enol-**O** in CD_3OD by UV light at rt followed by ^1H NMR spectroscopy (500 MHz) (ppm scale). Signals of the starting material are marked in blue color; signals of the major new component are marked in green color, and signals of the two new minor components are marked in red and purple colors.

During the irradiation of **1**-*cis*-enol-**O** with 365 nm light in CD_3OD (Figure S12 and S13), the pale-yellow solution turned green. After 30 min irradiation two new set of signals appeared in the ^1H NMR spectrum (Figure S12, 30 min). Interestingly, the intensity of the major set of new methyl signals (Figure 5, green color) increased during the reaction, while the signal intensity of the other new species (Figure S12, red color) stayed constant during the initial part of the irradiation. Additionally, after 90 minutes of irradiation the signals of the minor new species shifted (disappeared and appeared at new chemical shift values). This suggests the appearance of a new, third species after 90 minutes of irradiation (Figure S12, purple color), which is structurally similar to the species marked in red color.

After prolonged irradiation (up to 16 h), substantial conversion to the main product (Figure S12, green color) was achieved. This compound had highly shifted methyl signals compared to **1**-*cis*-enol-**O**, suggesting that it contains the closed form of the DTE moiety. Conversely, the other, minor photoproduct (Figure S12, purple color) had ^1H resonances at similar chemical shift values compared to the starting material.

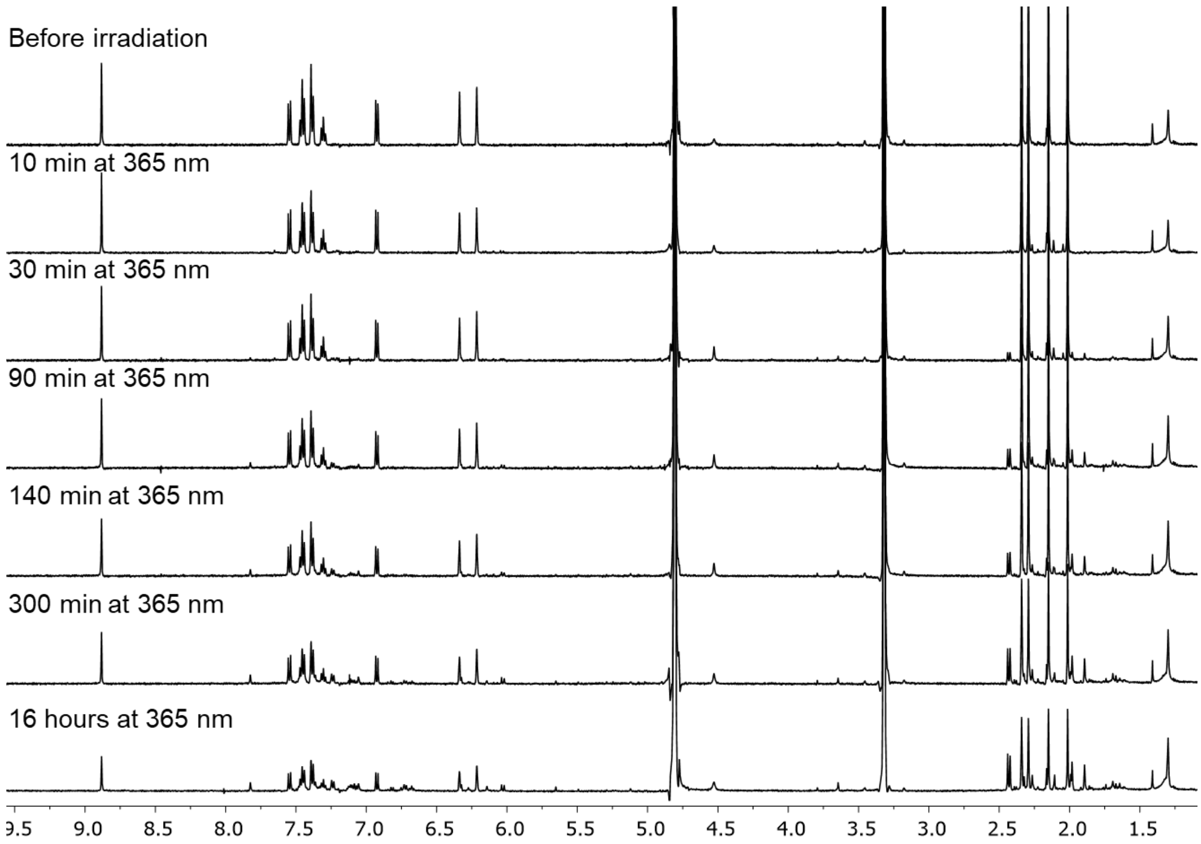

Figure S13. Irradiation of **1**-*cis*-enol-**O** in CD_3OD by UV light at rt followed by ^{1}H NMR spectroscopy (500 MHz) (ppm scale).

^{1}H NMR analysis of the isolated closed form 1-*cis*/*trans*-keto-C

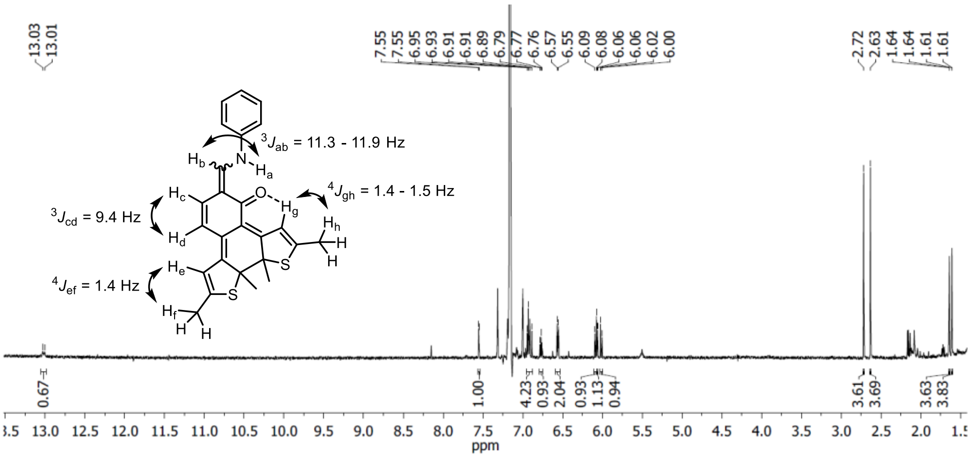

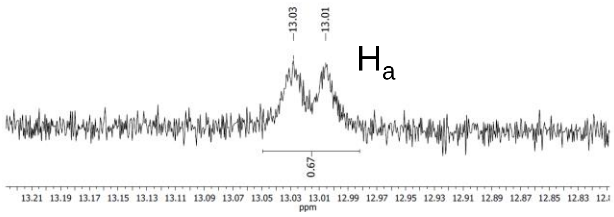

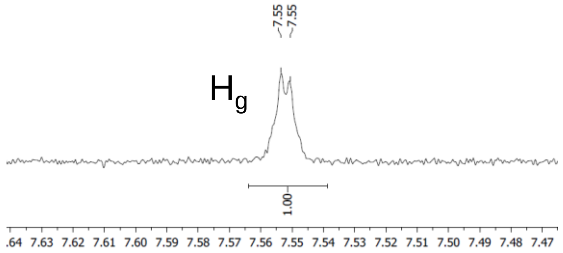

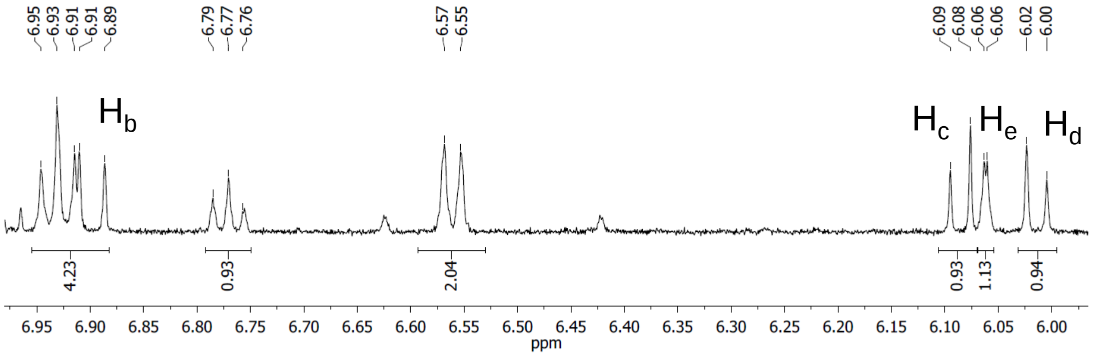

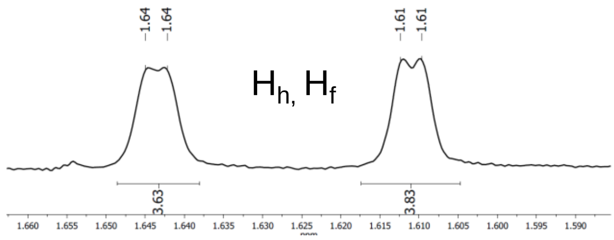

Name	Shift (ppm)	H	Multipl.	*J* (Hz)
Ha	13.02	1	d	11.27
Hb	6.90	1	d	11.89
Hc	6.09	1	d	9.39
Hd	6.01	1	d	9.40
He	6.06	1	d	1.38
Hf	1.61	3	d	1.36
Hg	7.55	1	d	1.46
Hh	1.64	3	d	1.37

Figure S14. ^{1}H NMR spectrum of the isolated closed form **1**-*cis*/*trans*-keto-**C** in C_6D_6 (500 MHz) and connectivity analysis.

The mobile proton of the SA moiety (H_a, Figure S14) appeared as a broad doublet in the ^{1}H NMR spectra at 13.02 ppm. This proton can only be a doublet if it is bound to the nitrogen of the SA moiety, therefore the isolated photoproduct contains the keto-form of the molecule. Through the analysis of ^3J coupling constants we can identify H_b, which is directly connected to the carbon atom bearing the imine/amine group. It was found that while H_b was at δ=8.12 ppm (in C_6D_6) in the parent molecule (**1**-*cis*-enol-**O**), it shifted significantly upfield (δ=6.90 ppm in C_6D_6), which also implies the presence of an enamine moiety in the photoproduct. Unfortunately, based on the analysis of the ^{1}H NMR spectra we could not unambiguously determine the stereochemistry around the double bond in the enamine moiety (*cis*/*trans* isomerism).

Further analysis of ^3J and ^4J connectivity in the ^{1}H NMR spectra revealed a substantial chemical shift difference between the two protons directly attached to the 2,5-dimethyltiophene rings (H_g δ=7.55 ppm and H_e δ=6.06 ppm). This is most likely due to the formation of an H-bond between H_g and the oxo-group of the SA moiety, resulting in an elongated a H_g-C bond and reduced electron density and electronic shielding around H_g compared to H_e.

S6 NMR spectra

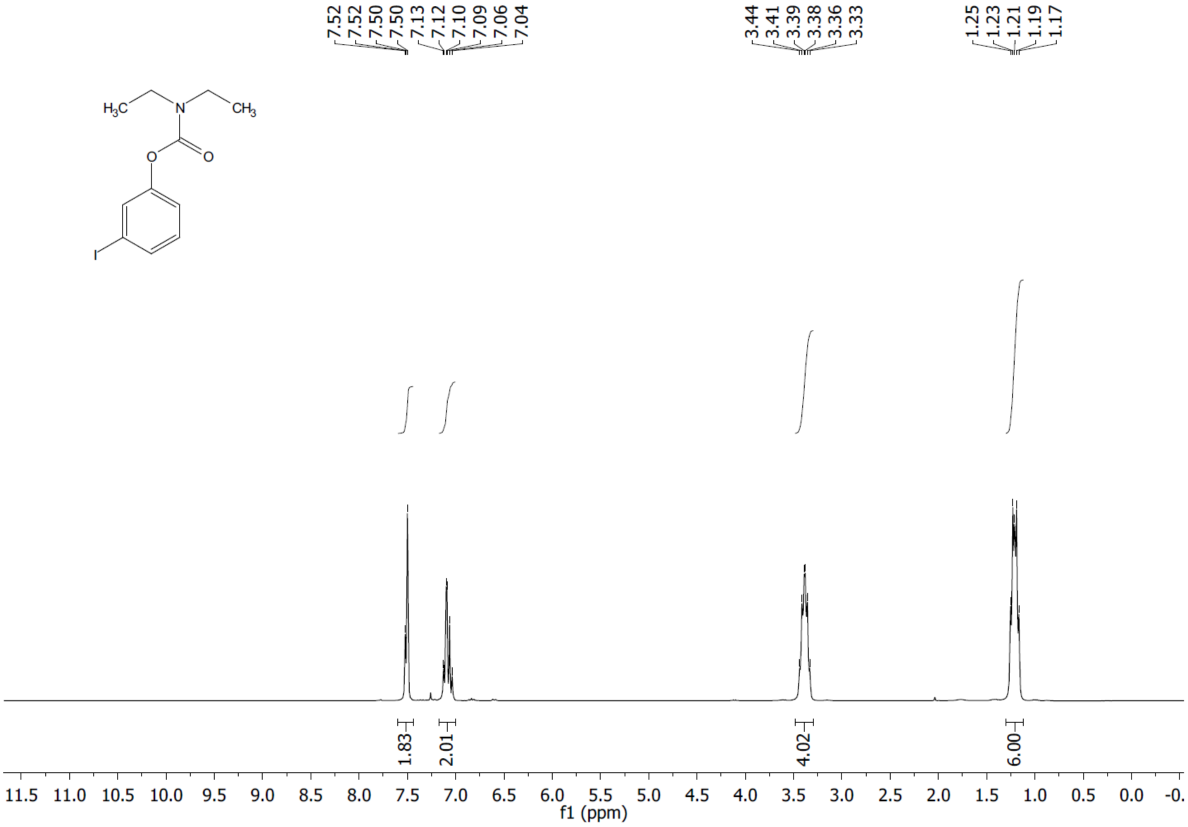

Figure S15. ^{1}H NMR spectrum of **3** in $CDCl_3$ (300 MHz).

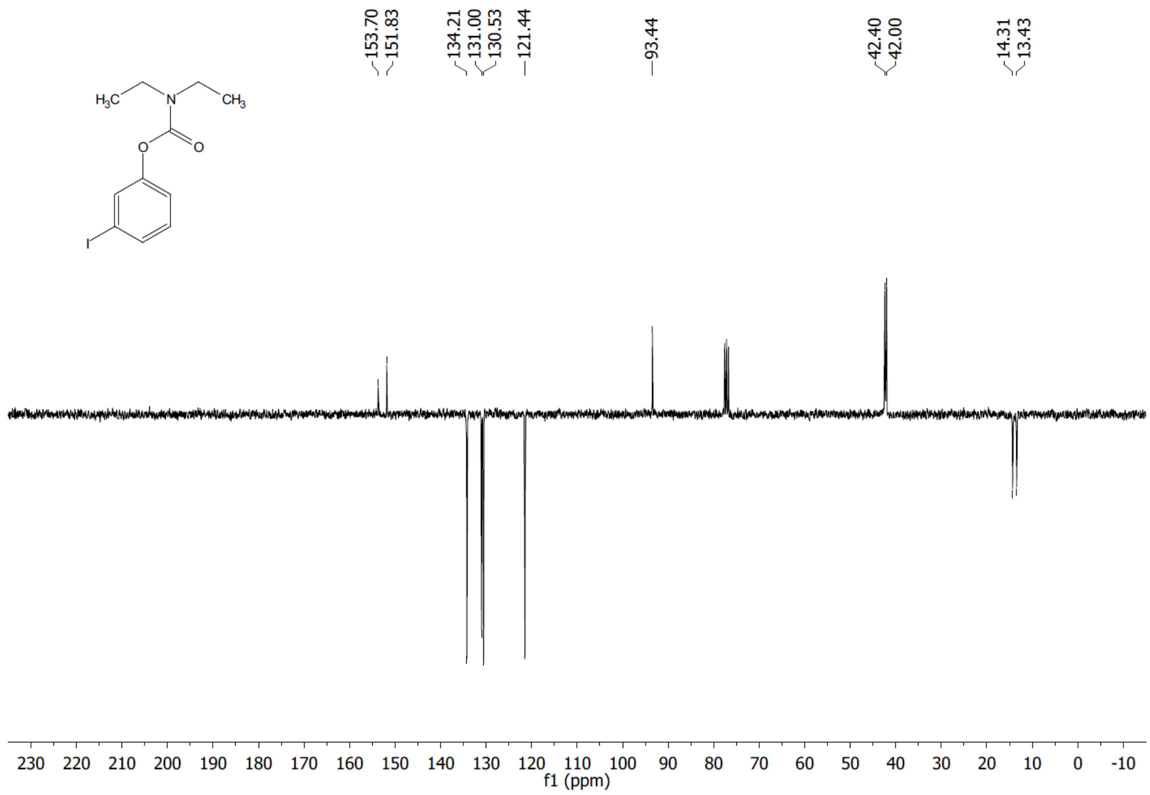

Figure S16. $^{13}C\{^{1}H\}$ NMR spectrum of **3** in $CDCl_3$ (75 MHz).

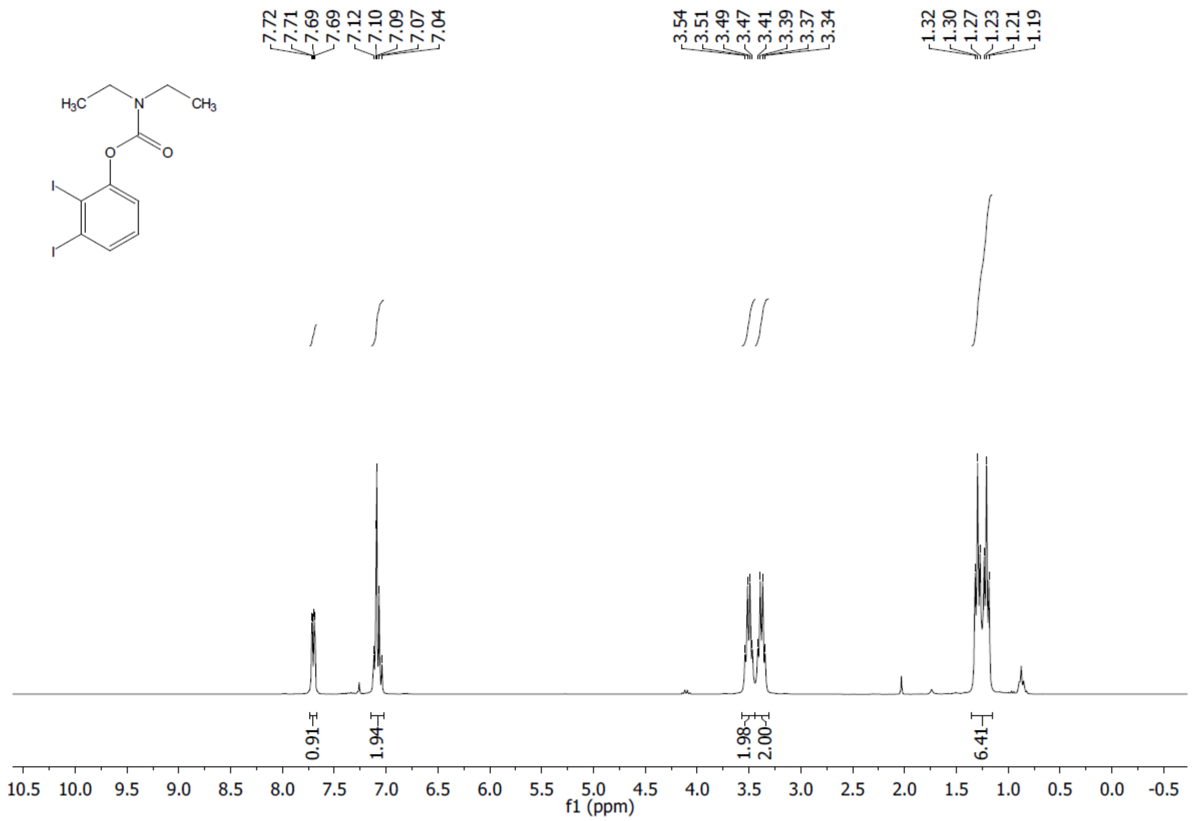

Figure S17. ^{1}H NMR spectrum of **4** in $CDCl_3$ (300 MHz).

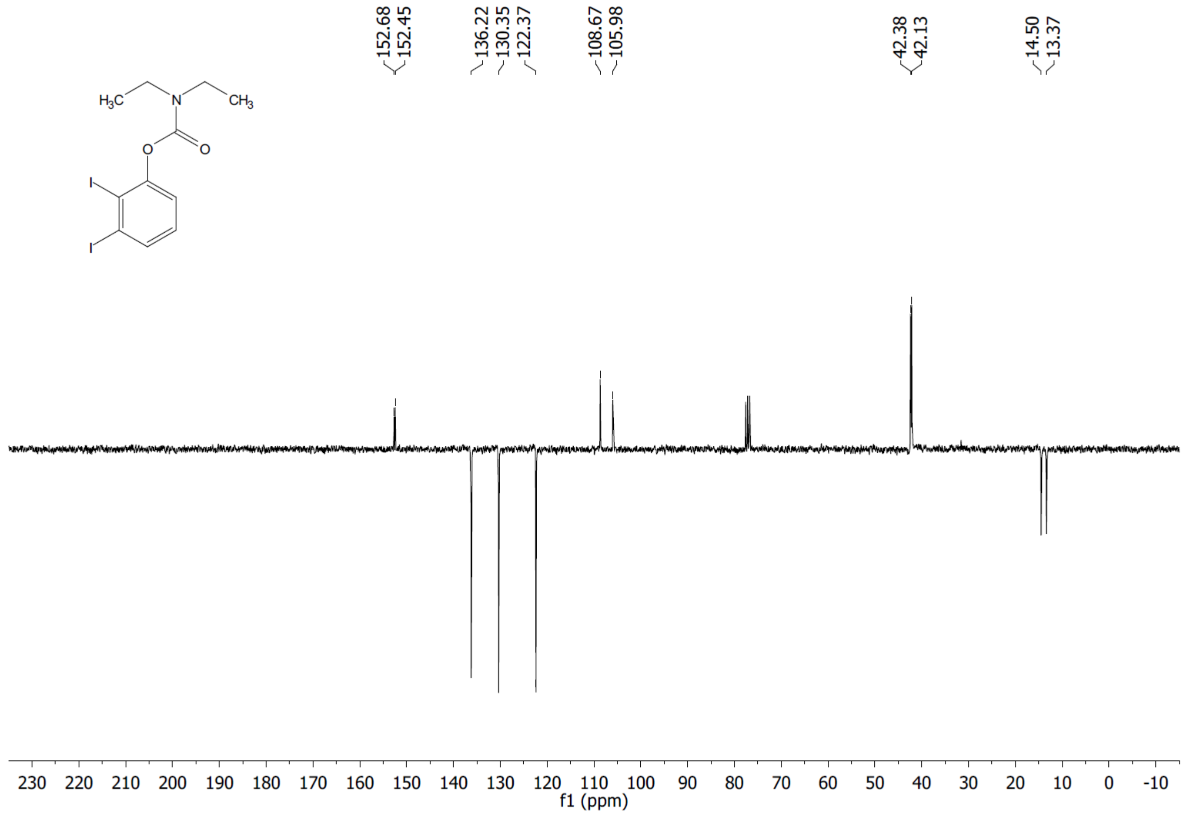

Figure S18. $^{13}C\{^{1}H\}$ NMR spectrum of **4** in $CDCl_3$ (75 MHz).

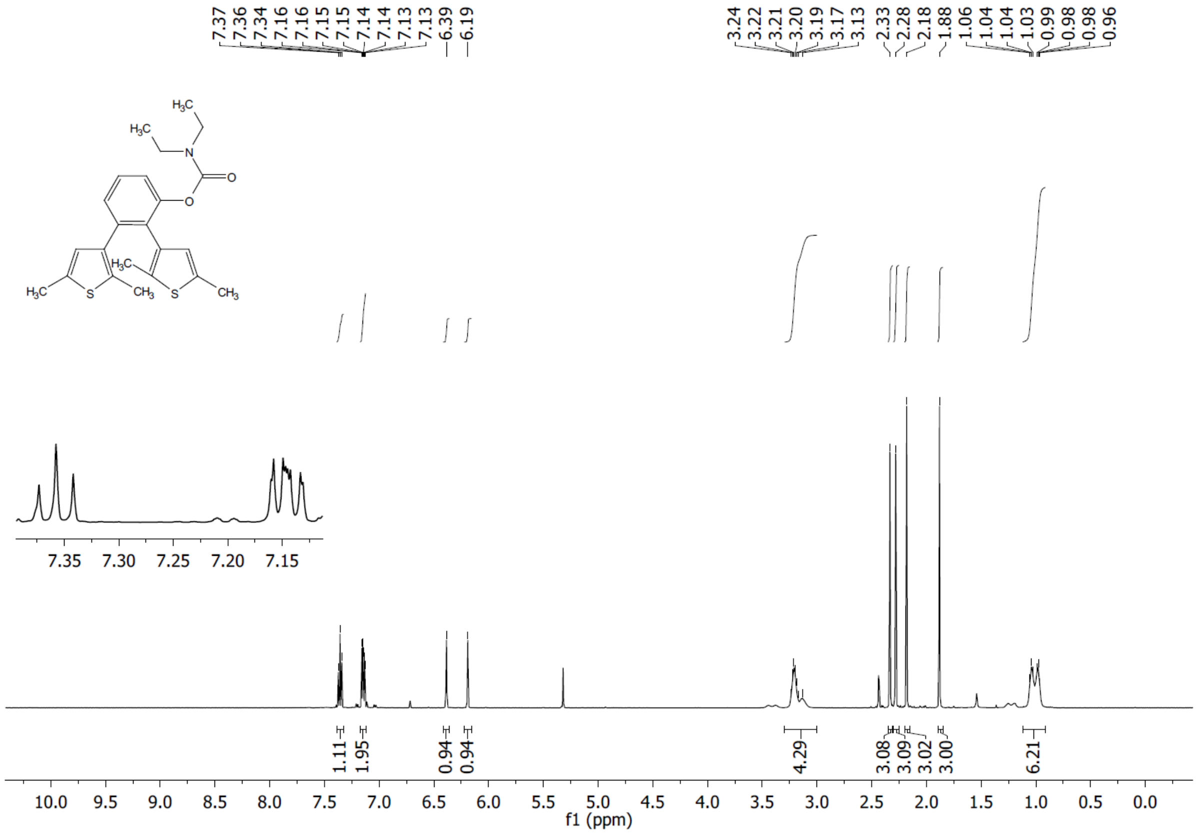

Figure S19. ^{1}H NMR spectrum of **6** in CD_2Cl_2 (500 MHz).

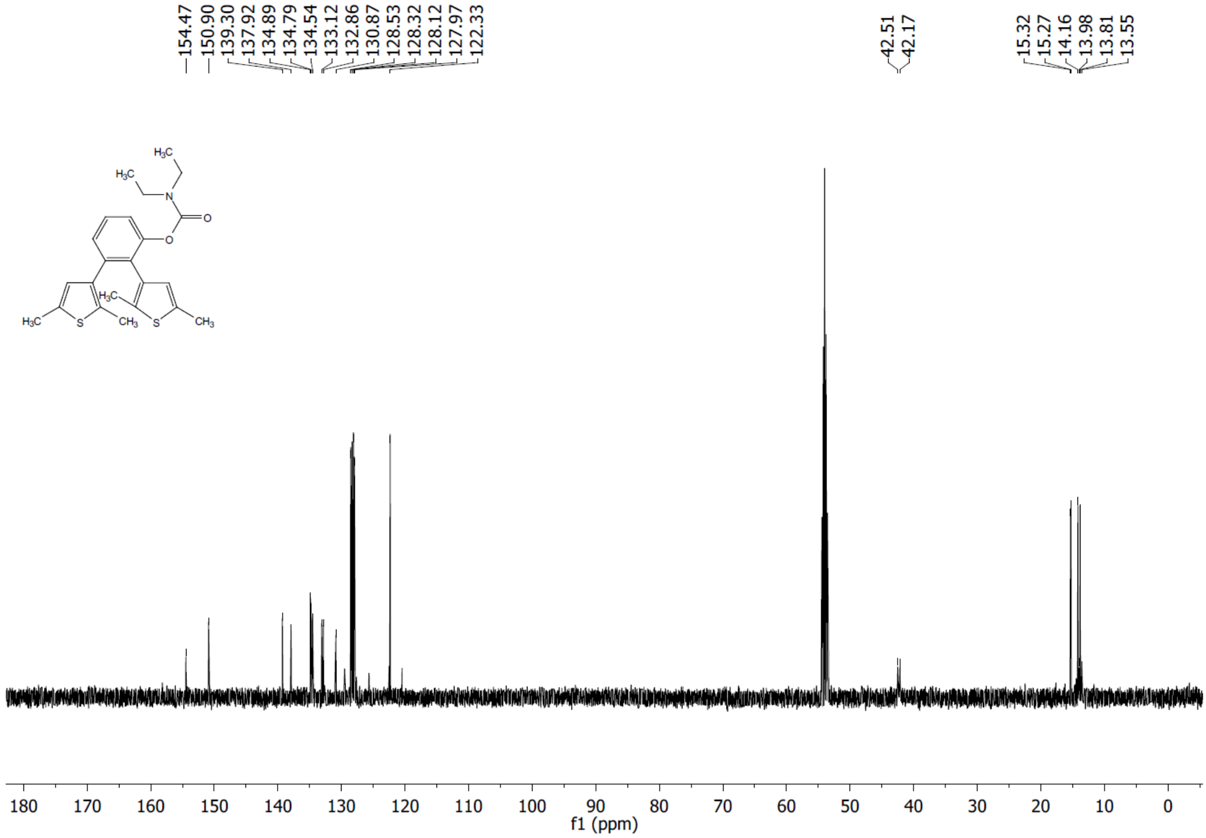

Figure S20. $^{13}C\{^{1}H\}$ NMR spectrum of **6** in CD_2Cl_2 (126 MHz).

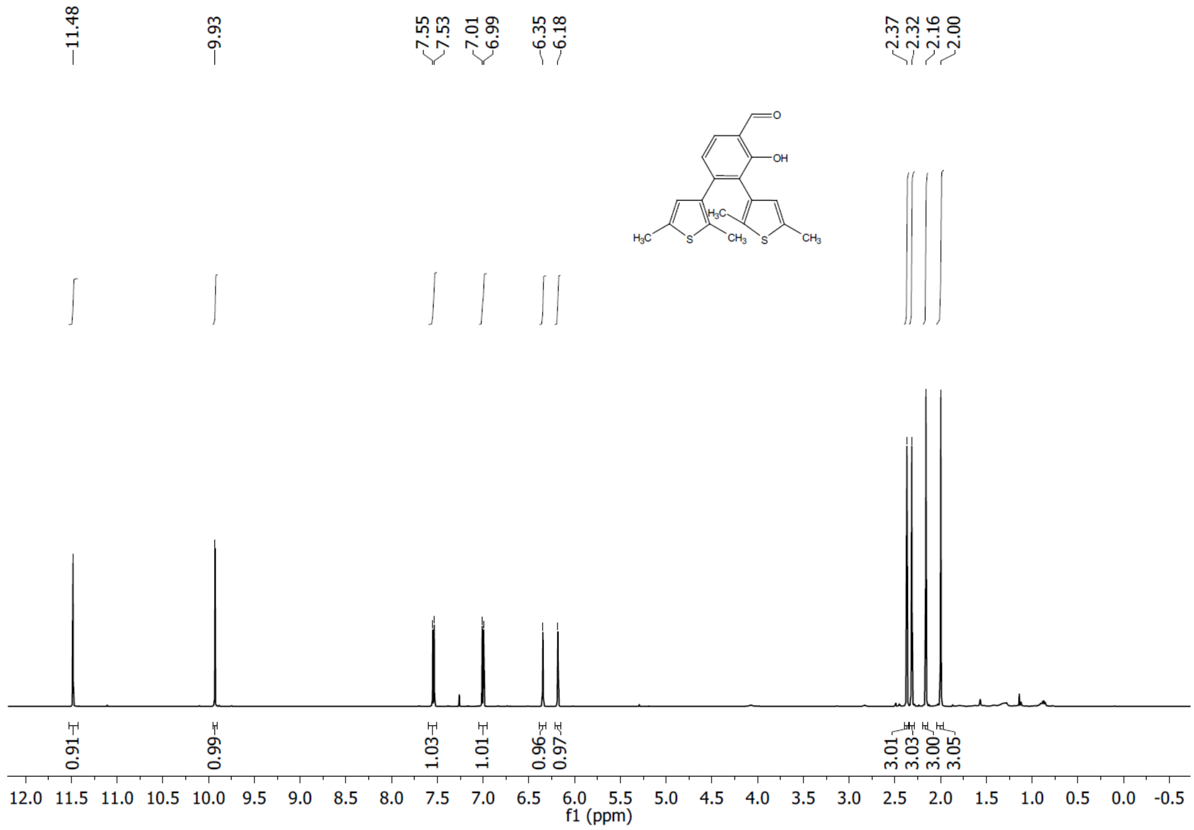

Figure S21. ^{1}H NMR spectrum of **7** in $CDCl_3$ (500 MHz).

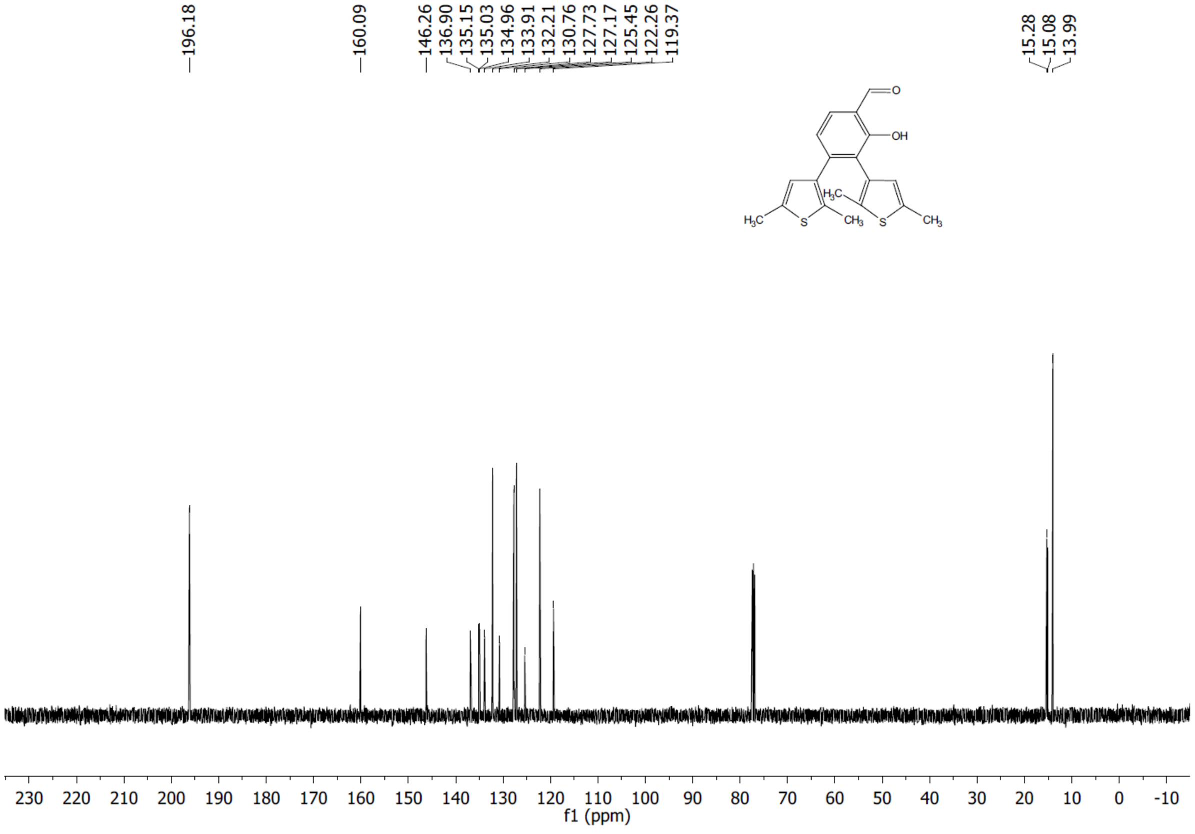

Figure S22. $^{13}C\{^{1}H\}$ NMR spectrum of **7** in $CDCl_3$ (126 MHz).

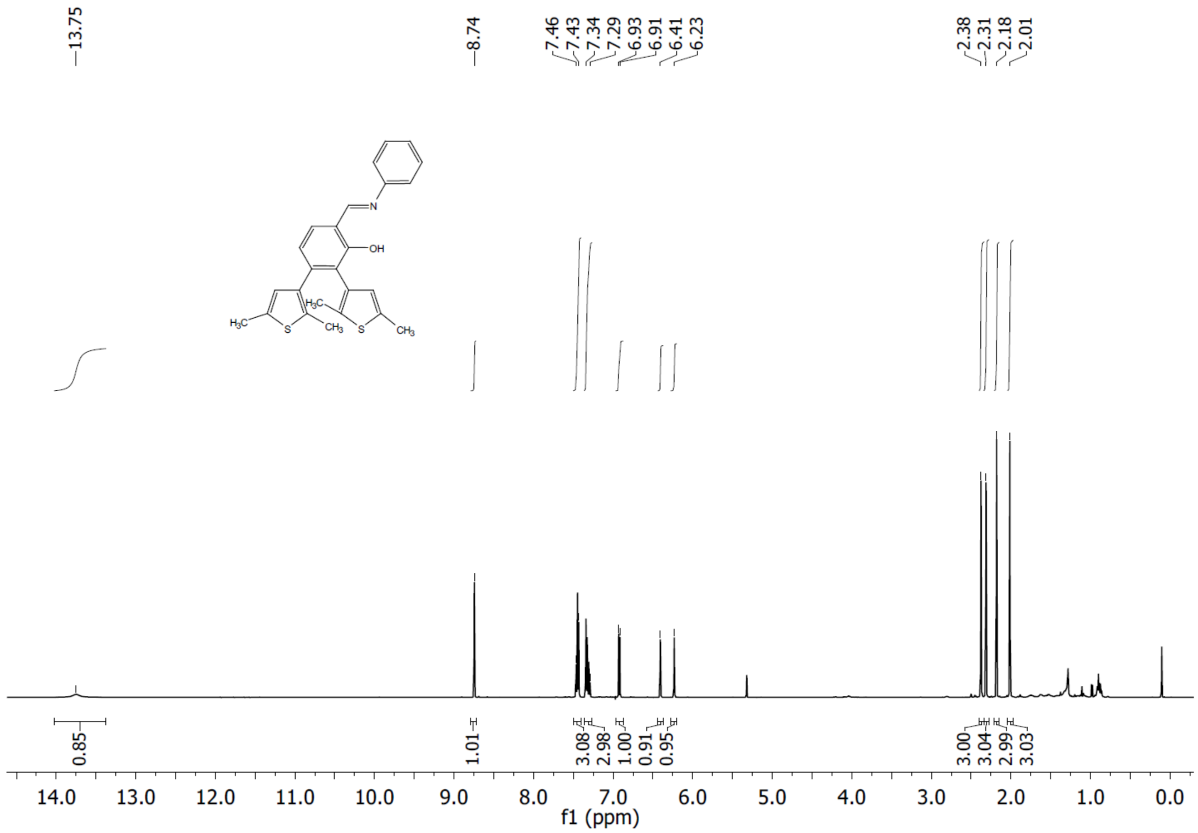

Figure S23. ^{1}H NMR spectrum of **1**-*cis*-enol-**O** in CD_2Cl_2 (500 MHz).

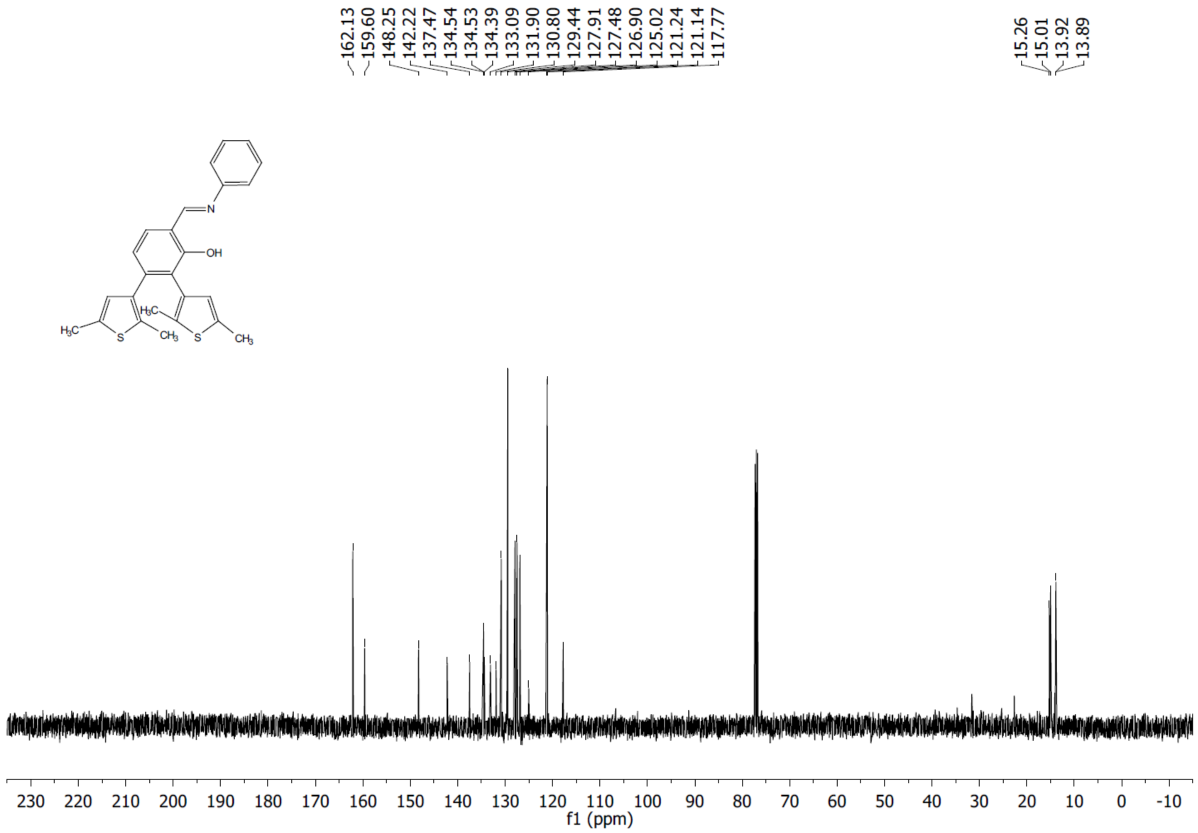

Figure S24. ^{13}C{^{1}H} NMR spectrum of **1**-*cis*-enol-**O** in $CDCl_3$ (126 MHz).

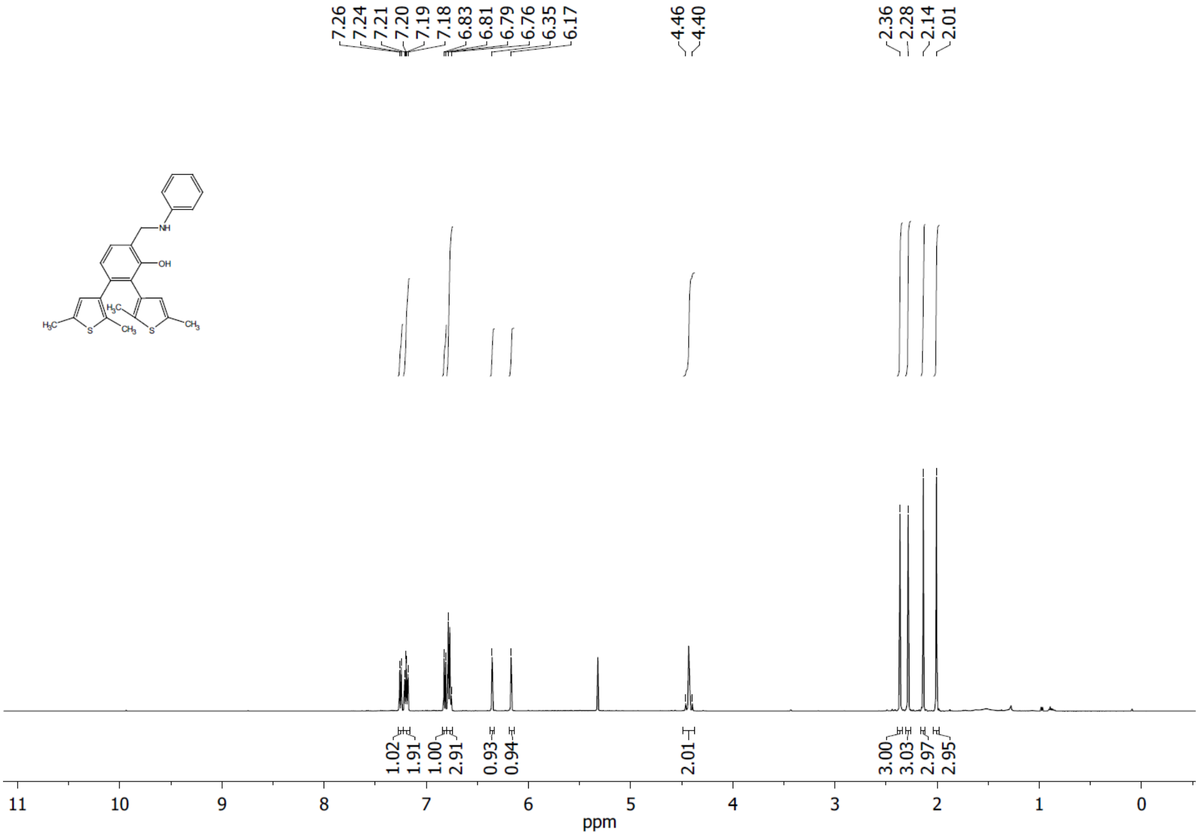

Figure S25. ^{1}H NMR spectrum of **8** in CD_2Cl_2 (500 MHz).

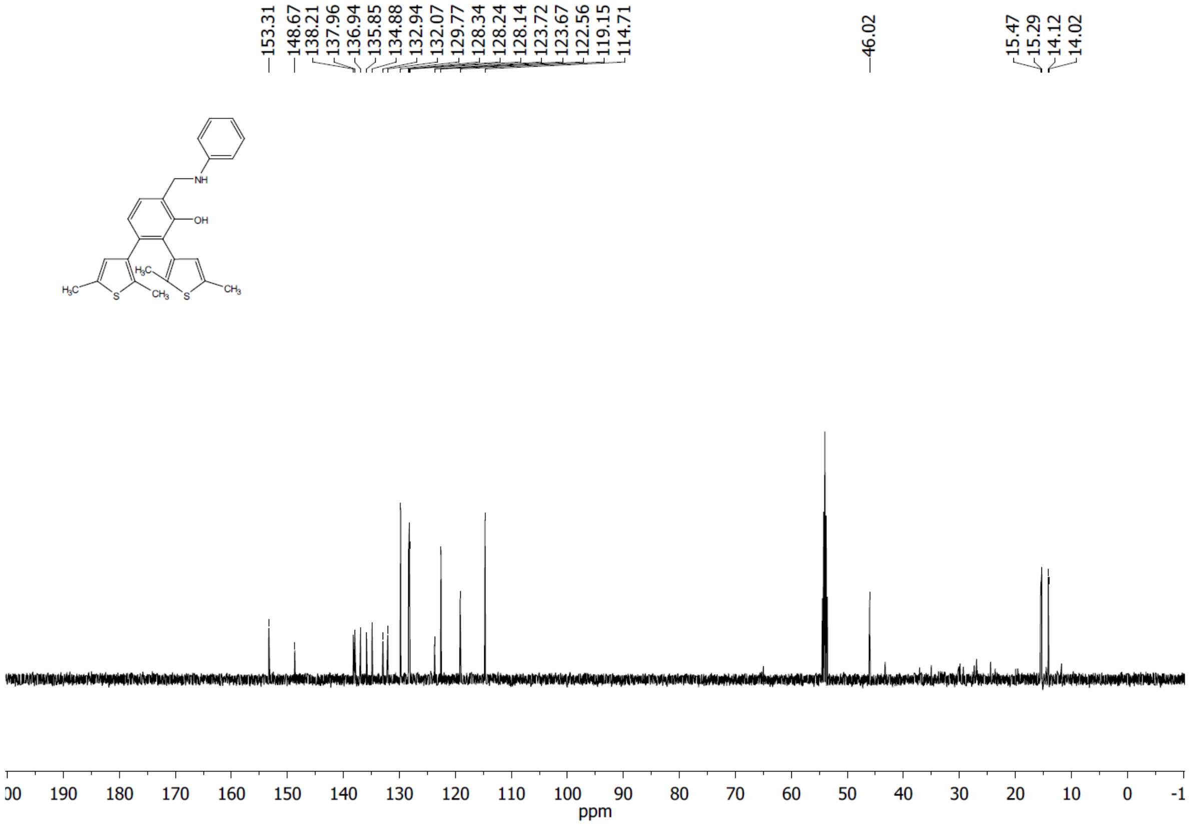

Figure S26. $^{13}C\{^{1}H\}$ NMR spectrum of **8** in CD_2Cl_2 (126 MHz).

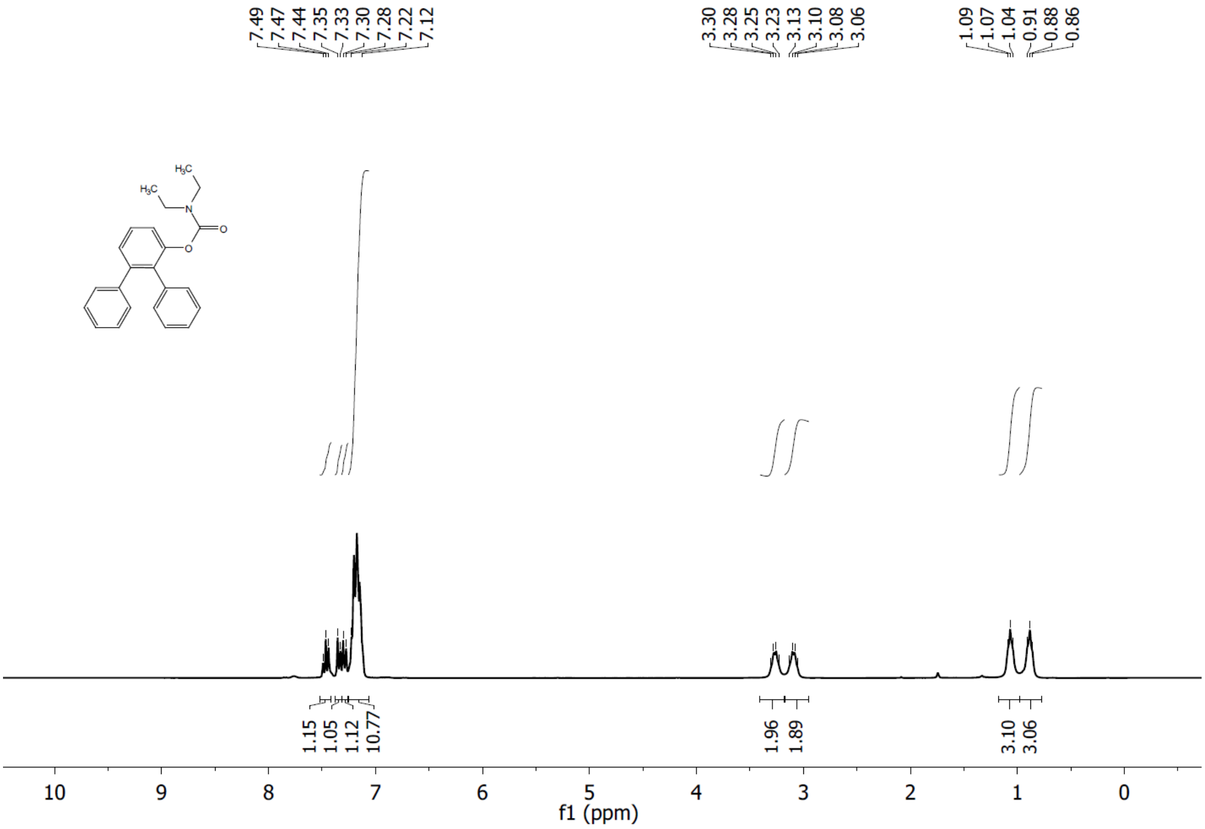

Figure S27. ^{1}H NMR spectrum of **S1** in $CDCl_3$ (300 MHz).

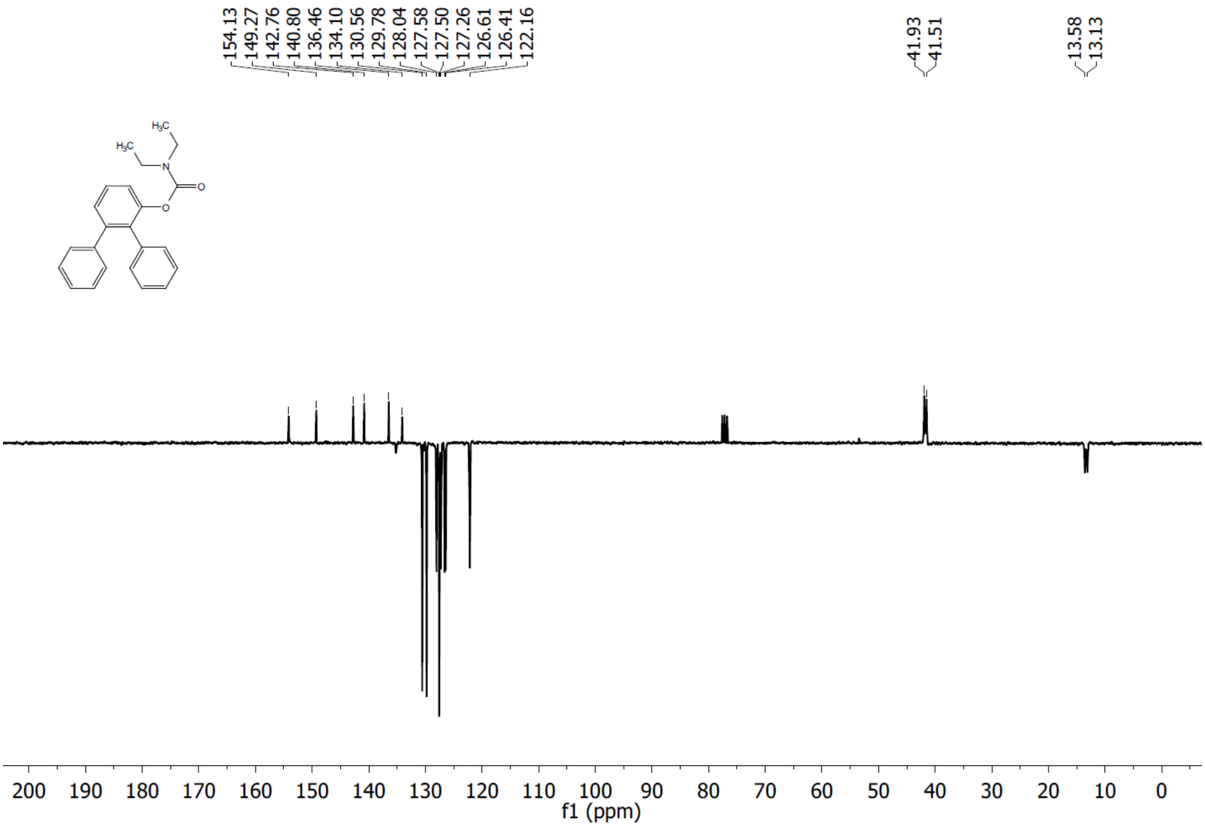

Figure S28. ^{13}C{^{1}H} NMR spectrum of **S1** in $CDCl_3$ (75 MHz).

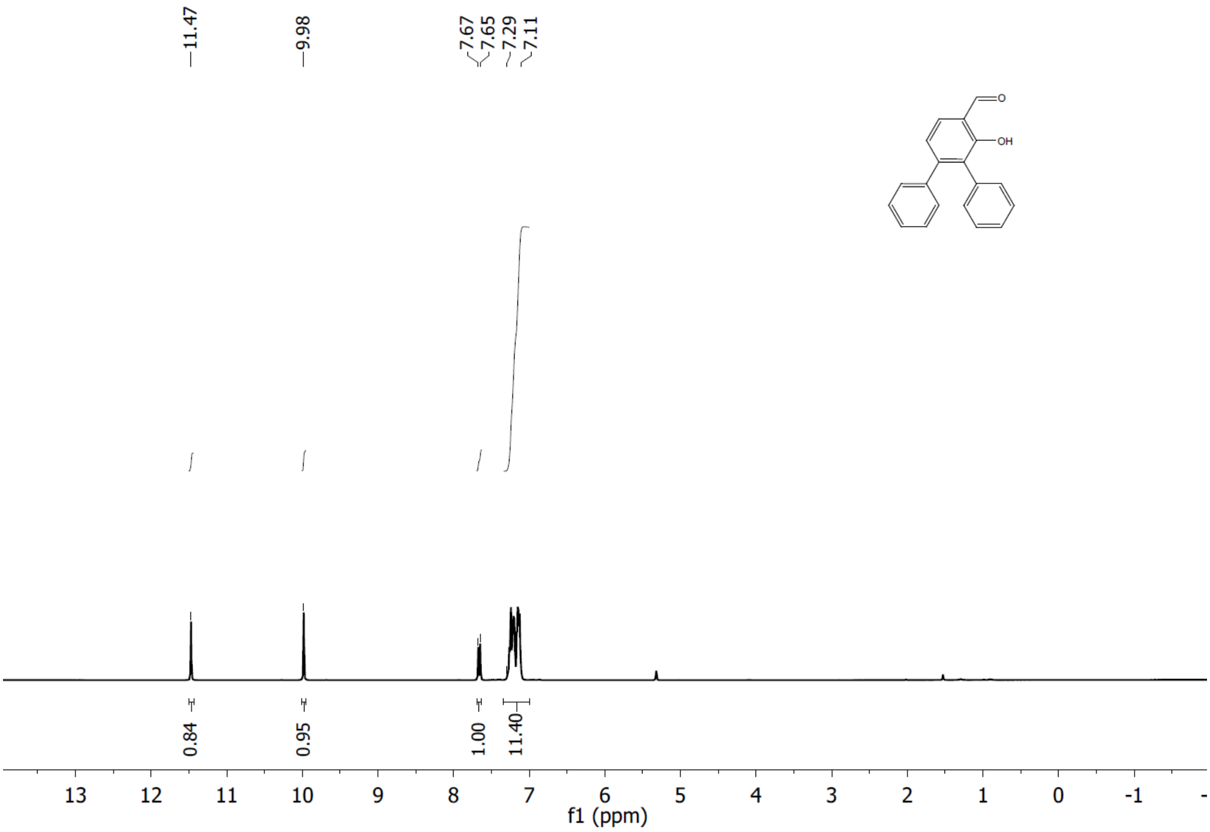

Figure S29. ^{1}H NMR spectrum of **S2** in CD_2Cl_2 (300 MHz).

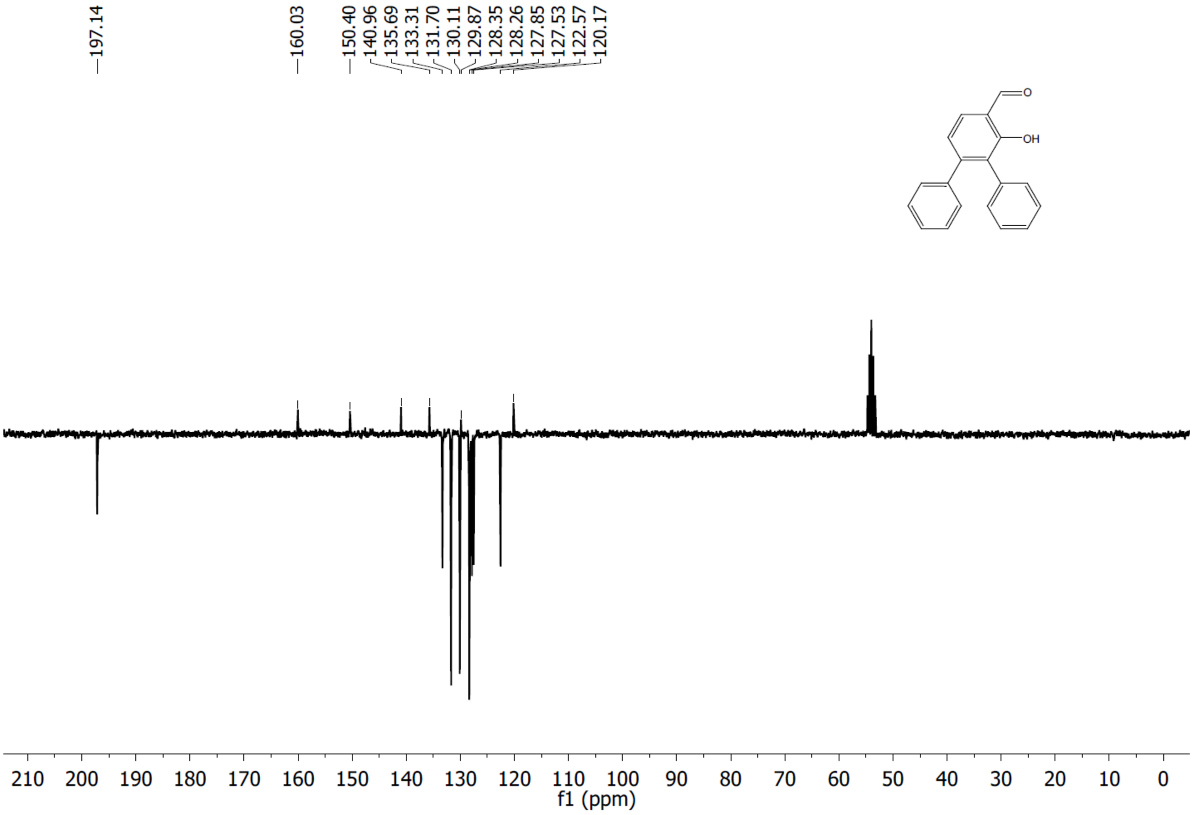

Figure S30. $^{13}C\{^{1}H\}$ NMR spectrum of **S2** in CD_2Cl_2 (75 MHz).

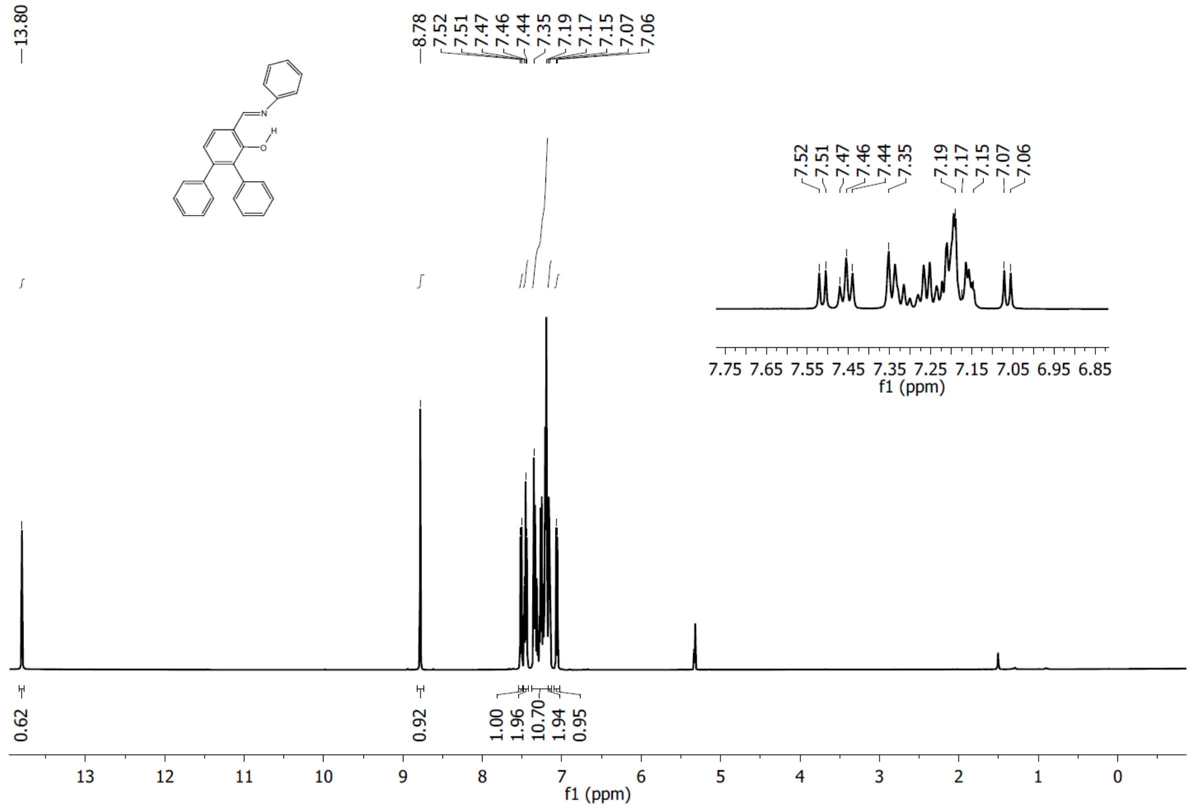

Figure S31. ^{1}H NMR spectrum of **9** in CD_2Cl_2 (500 MHz).

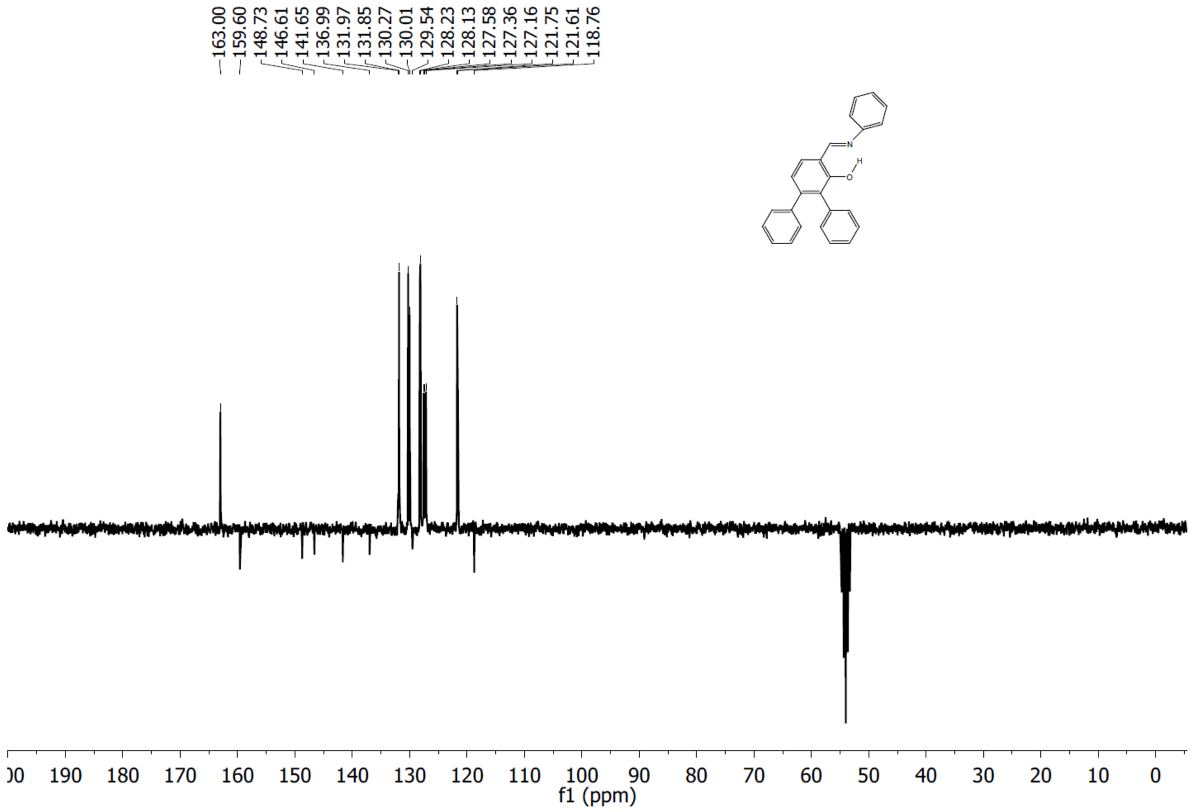

Figure S32. $^{13}C\{^{1}H\}$ NMR spectrum of **9** in CD_2Cl_2 (75 MHz).

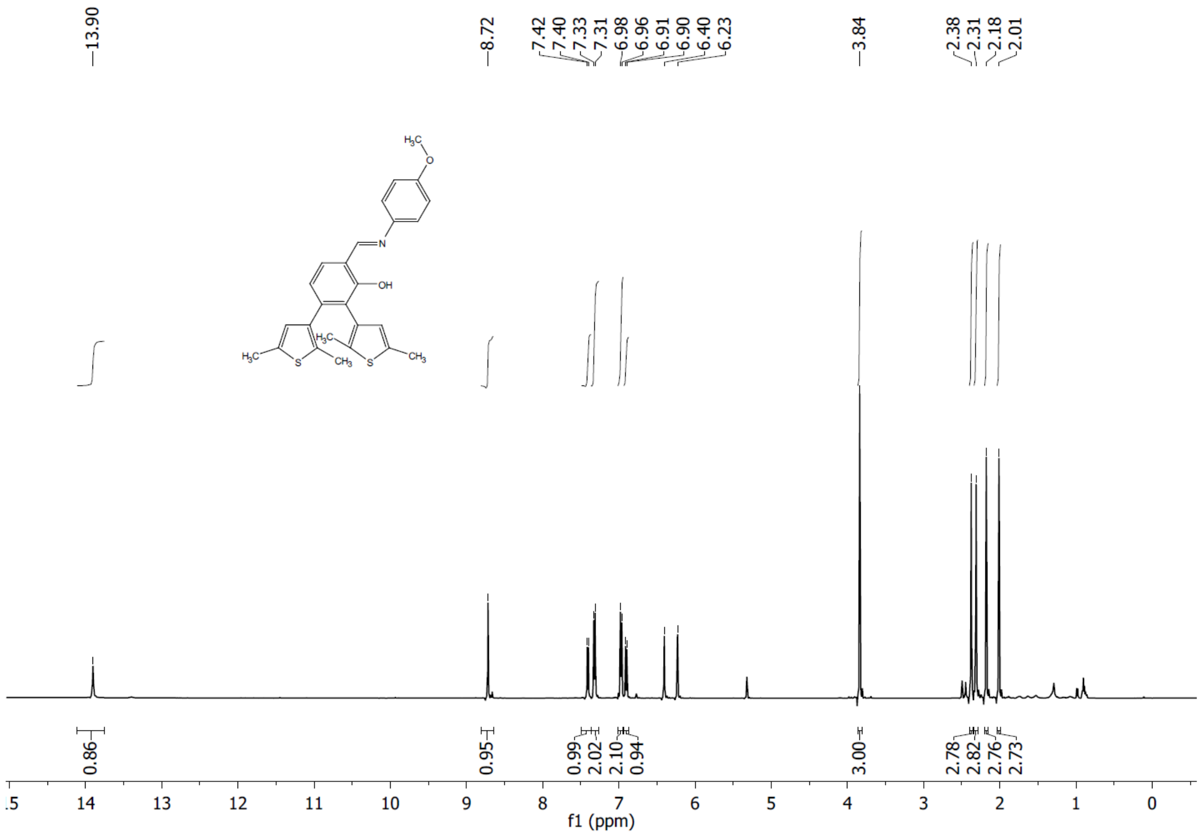

Figure S33. ^{1}H NMR spectrum of **12** in CD_2Cl_2 (500 MHz).

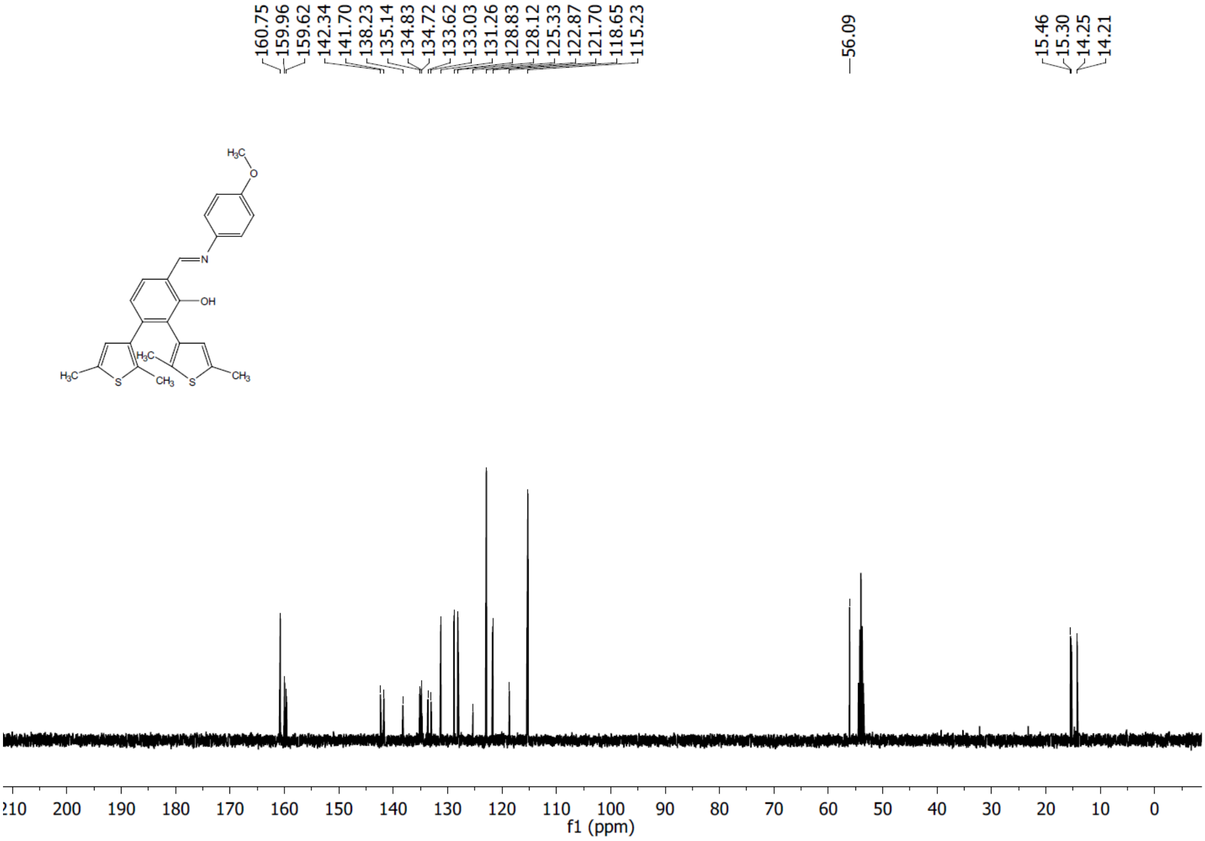

Figure S34. $^{13}C\{^{1}H\}$ NMR spectrum of **12** in CD_2Cl_2 (126 MHz).

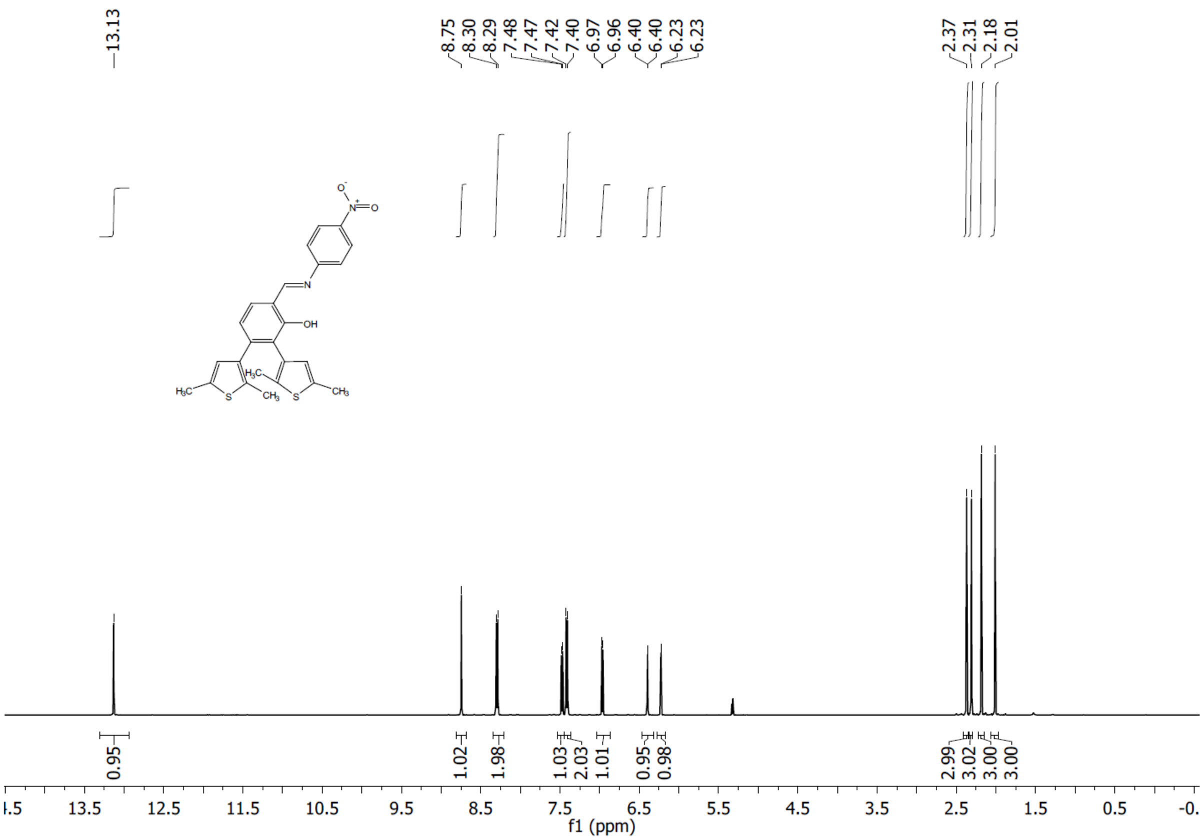

Figure S35. ^{1}H NMR spectrum of **13** in CD_2Cl_2 (500 MHz).

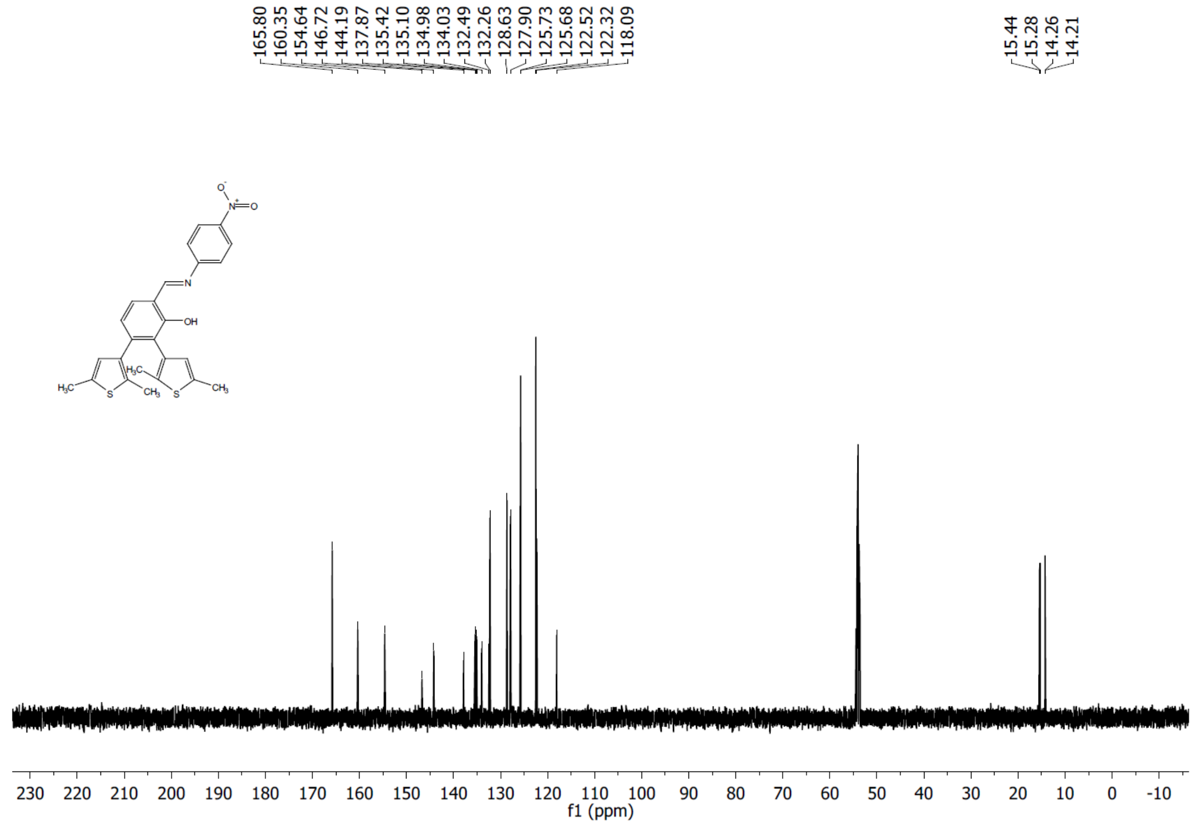

Figure S36. $^{13}C\{^{1}H\}$ NMR spectrum of **13** in CD_2Cl_2 (126 MHz).

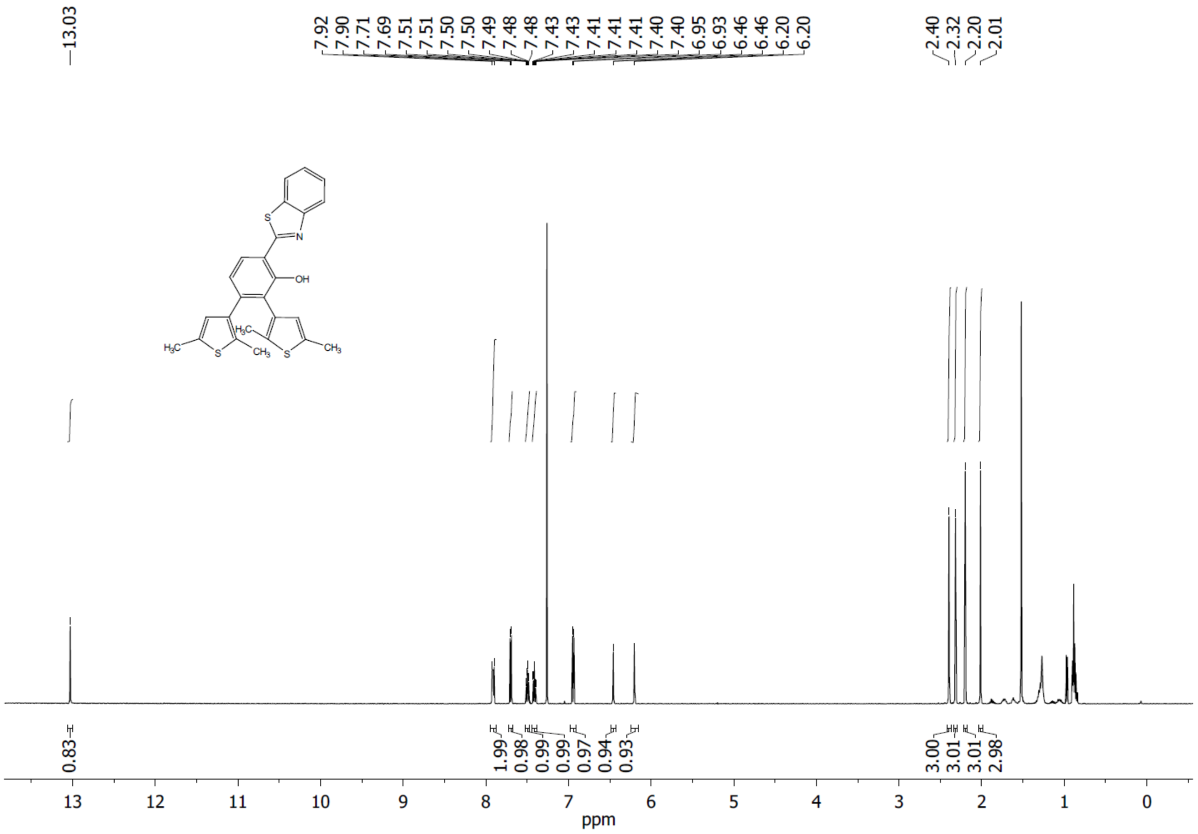

Figure S37. ^{1}H NMR spectrum of **14** in $CDCl_3$ (500 MHz).

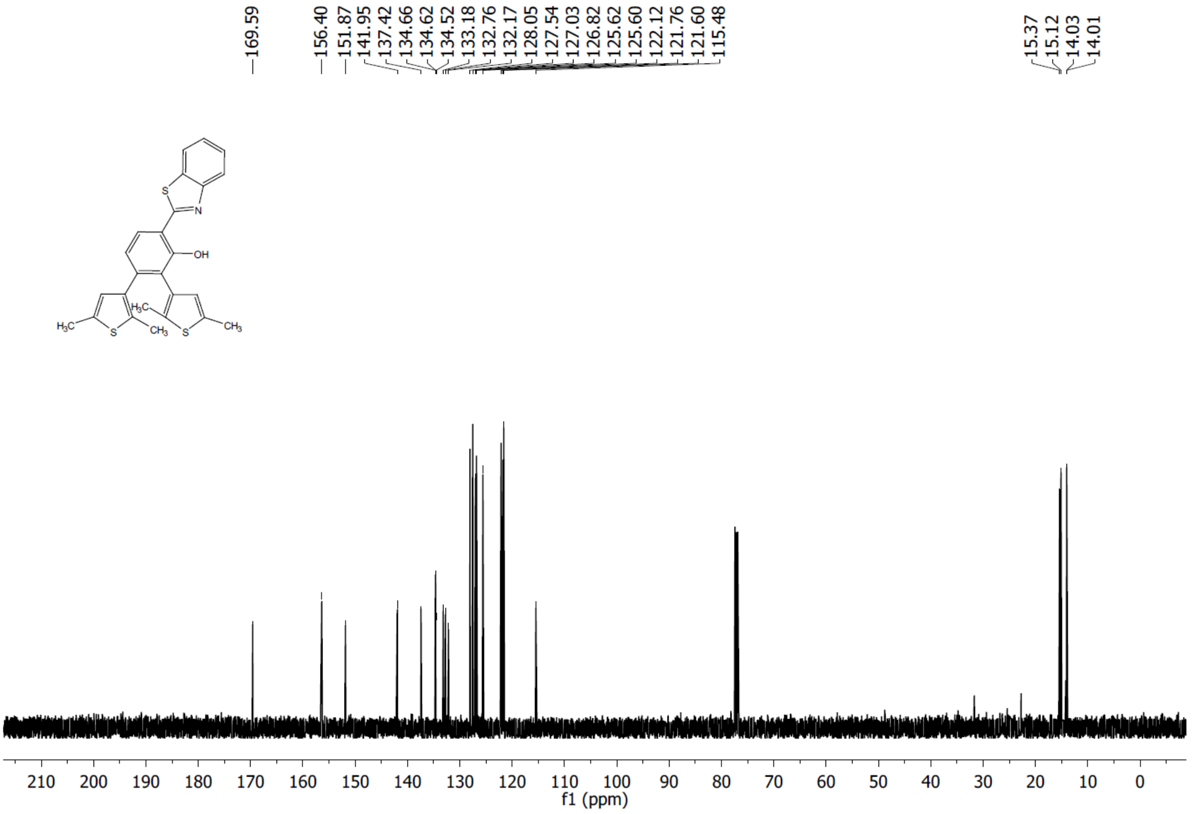

Figure S38. $^{13}C\{^{1}H\}$ NMR spectrum of **14** in $CDCl_3$ (126 MHz).

S7 References

1 CrystalClear SM 1.4.0 Rigaku/MSC Inc., 2008.

2 NUMABS: Higashi, T. (1998), rev. 2002. (Rigaku/MSC Inc.)

3 Sheldrick, G. M. A Short History of SHELX. *Acta Cryst.* **2008**, *A64*, 112–122. DOI: 10.1107/S0108767307043930

4 Sheldrick, G. M. *Acta Cryst.* Crystal Structure Refinement with SHELXL. **2015**, *C71*, 3–8. DOI: 10.1107/S2053229614024218

5 Farrugia, L. J. WinGX and ORTEP for Windows: An Update. *J. Appl. Crystallogr.* **2012**, *45*, 849–854. DOI: 10.1107/S0021889812029111

6 Spek, A. L. Structure Validation in Chemical Crystallography. *Acta Cryst.* **2009**, *D65*, 148–155. DOI: 10.1107/S090744490804362X

7 Dolomanov, O. V.; Bourhis, L. J.; Gildea, R. J.; Howard, J. A. K.; Puschmann, H. OLEX2: A Complete Structure Solution, Refinement and Analysis Program. *J. Appl. Cryst.* **2009**, *42*, 339-341. DOI: 10.1107/S0021889808042726

8 Macrae, C. F.; Edgington, P. R.; McCabe, P.; Pidcock, E.; Shields, G. P.; Taylor, R.; Towler, M.; van de Streek, J. Mercury: Visualization and Analysis of Crystal Structures. *J. Appl. Cryst*. **2006**, *39*, 453-457. DOI: 10.1107/S002188980600731X

9 Gaussian 16, Revision A.03, Frisch, M. J.; Trucks, G. W.; Schlegel, H. B.; Scuseria, G. E.; Robb, M. A.; Cheeseman, J. R.; Scalmani, G.; Barone, V.; Petersson, G. A.; Nakatsuji, H.; Li, X.; Caricato, M.; Marenich, A. V.; Bloino, J.; Janesko, B. G.; Gomperts, R.; Mennucci, B.; Hratchian, H. P.; Ortiz, J. V.; Izmaylov, A. F.; Sonnenberg, J. L.; Williams-Young, D.; Ding, F.; Lipparini, F.; Egidi, F.; Goings, J.; Peng, B.; Petrone, A.; Henderson, T.; Ranasinghe, D.; Zakrzewski, V. G.; Gao, J.; Rega, N.; Zheng, G.; Liang, W.; Hada, M.; Ehara, M.; Toyota, K.; Fukuda, R.; Hasegawa, J.; Ishida, M.; Nakajima, T.; Honda, Y.; Kitao, O.; Nakai, H.; Vreven, T.; Throssell, K.; Montgomery, J. A., Jr.; Peralta, J. E.; Ogliaro, F.; Bearpark, M. J.; Heyd, J. J.; Brothers, E. N.; Kudin, K. N.; Staroverov, V. N.; Keith, T. A.; Kobayashi, R.; Normand, J.; Raghavachari, K.; Rendell, A. P.; Burant, J. C.; Iyengar, S. S.; Tomasi, J.; Cossi, M.; Millam, J. M.; Klene, M.; Adamo, C.; Cammi, R.; Ochterski, J. W.; Martin, R. L.; Morokuma, K.; Farkas, O.; Foresman, J. B.; Fox, D. J. Gaussian, Inc., Wallingford CT, 2016.

10 Lee, C.; Yang, W.; Parr, R. G. Development of the Colle-Salvetti Correlation-Energy Formula into a Functional of the Electron Density. *Phys. Rev. B* **1988**, *37*, 785–789. DOI: 10.1103/PhysRevB.37.785

11 Becke, A. D. Density-Functional Thermochemistry. III. The Role of Exact Exchange. *J. Chem. Phys.* **1993**, *98*, 5648–5652. DOI: 10.1063/1.464913

12 Stephens, P. J.; Devlin, F. J.; Chabalowski, C. F.; Frisch, M. J. *Ab Initio* Calculation of Vibrational Absorption and Circular Dichroism Spectra Using Density Functional Force Fields. *J. Phys. Chem.* **1994**, *98*, 11623–11627. DOI: 10.1021/j100096a001

13 Weigend, F.; Ahlrichs, R. Balanced Basis Sets of Split Valence, Triple Zeta Valence and Quadruple Zeta Valence Quality for H to Rn: Design and Assessment of Accuracy. *Phys. Chem. Chem. Phys.* **2005**, *7*, 3297–3305. DOI: 10.1039/b508541a

14 Grimme, S.; Antony, J.; Ehrlich, S.; Krieg, H. A Consistent and Accurate *Ab Initio* Parameterization of Density Functional Dispersion Correction (DFT-D) for the 94 Elements H-Pu. *J. Chem. Phys.* **2010**, *132*, 154104. DOI: 10.1063/1.3382344

15 Ortiz-Sánchez, J. M.; Gelabert, R.; Morenoa, M.; Lluch, J. M. Electronic-Structure and Quantum Dynamical Study of the Photochromism of the Aromatic Schiff Base Salicylideneaniline. *J. Chem. Phys.* **2008**, *129*, 214308. DOI: 10.1063/1.3032215

16 Jankowska, J.; Rode, M. F.; Sadlej, J.; Sobolewski, A. L. Photophysics of Schiff Bases: Theoretical Study of Salicylidene Methylamine. *ChemPhysChem* **2012**, *13*, 4287–4294. DOI: 10.1002/cphc.201200560

17 Spörkel, L.; Cui, G.; Thiel, W. Photodynamics of Schiff Base Salicylideneaniline: Trajectory Surface-Hopping Simulations. *J. Phys. Chem. A* **2013**, *117*, 4574–4583. DOI: 10.1021/jp4028035

18 Barbatti, M.; Aquino, A. J. A.; Lischka, H. The UV absorption of Nucleobases: Semi-Classical Ab Initio Spectra Simulations. *Phys. Chem. Chem. Phys.* **2010**, *12*, 4959–4967. DOI: 10.1039/B924956G

19 Crespo-Otero, R.; Barbatti, M. Spectrum Simulation and Decomposition with Nuclear Ensemble: Formal Derivation and Application to Benzene, Furan and 2-Phenylfuran. *Theor. Chem. Acc.* **2012**, *131*, 1237. DOI: 10.1007/s00214-012-1237-4

20 Neese, F. Software Update: The ORCA Program System – Version 5.0. *WIREs Comput. Mol. Sci.* **2022**, *12*, e1606. DOI: 10.1002/wcms.1606

21 Yanai, T.; Tew, D. P.; Handy, N. C. A New Hybrid Exchange–Correlation Functional Using the Coulomb-Attenuating Method (CAM-B3LYP). *Chem. Phys. Lett.* **2004**, *393*, 51–57. DOI: 10.1016/j.cplett.2004.06.011

22 Laurent, A. D.; Jacquemin, D. TD-DFT Benchmarks: A Review. *Int. J. Quantum Chem.* **2013**, *113*, 2019–2039. DOI: 10.1002/qua.24438

23 Batra, K.; Zahn, S.; Heine, T. Benchmark of Simplified Time-Dependent Density Functional Theory for UV–Vis Spectral Properties of Porphyrinoids. *Adv. Theory Simul.* **2020**, *3*, 1900192. DOI: 10.1002/adts.201900192

S8 Cartesian coordinates (in Å) of optimized geometries of compound 1

cis-enol-**I**

C	1.195681000	-0.510739000	0.693962000
C	0.950255000	-1.903480000	0.649887000
C	1.011233000	-2.706307000	-0.597601000
C	0.351554000	-2.438114000	-1.781060000
S	0.720005000	-3.652785000	-2.979060000
C	1.749560000	-4.544993000	-1.886214000
C	1.787509000	-3.918635000	-0.670030000
C	-0.577776000	-1.316134000	-2.126173000
C	2.452340000	-5.795881000	-2.318329000
C	1.558695000	0.299182000	-0.497995000
C	2.592050000	0.018571000	-1.368723000
S	2.633524000	1.178763000	-2.673484000
C	1.271937000	2.070423000	-2.032900000
C	0.830369000	1.482262000	-0.880863000
C	3.599513000	-1.088724000	-1.329260000
C	0.749461000	3.295848000	-2.719044000
C	0.651929000	-2.606243000	1.844117000
C	0.567985000	-1.947834000	3.055557000
C	0.784137000	-0.557247000	3.136444000
C	1.098038000	0.160647000	1.945043000
C	0.694403000	0.121573000	4.412787000
N	0.885951000	1.393449000	4.535901000
C	0.854472000	2.048361000	5.775321000
C	1.297894000	1.454199000	6.973771000
C	1.246125000	2.166581000	8.173264000
C	0.758466000	3.477376000	8.199547000
C	0.333144000	4.079021000	7.009391000
C	0.389951000	3.376737000	5.805900000
O	1.318286000	1.473067000	1.984809000
H	-0.059615000	-0.524302000	-2.690922000
H	-0.973201000	-0.855459000	-1.211065000
H	-1.427354000	-1.669378000	-2.732049000
H	3.144043000	-5.610457000	-3.157398000

H	1.742752000	-6.574376000	-2.645806000
H	3.038863000	-6.203562000	-1.481428000
H	3.645035000	-1.523243000	-0.321209000
H	3.335959000	-1.902637000	-2.024671000
H	4.605777000	-0.726611000	-1.594342000
H	0.410789000	3.081280000	-3.746887000
H	1.514499000	4.087958000	-2.785716000
H	-0.106641000	3.700535000	-2.158670000
H	1.209452000	1.767895000	2.938790000
H	0.435491000	-0.504536000	5.286326000
H	0.323561000	-2.500647000	3.967403000
H	0.460138000	-3.679089000	1.788655000
H	0.065268000	3.837458000	4.870282000
H	1.713818000	0.444285000	6.958180000
H	-0.040302000	5.106246000	7.018369000
H	0.723063000	4.032055000	9.140255000
H	1.601749000	1.696845000	9.094115000
H	2.383926000	-4.289054000	0.165931000
H	-0.002591000	1.875077000	-0.297479000

cis-enol-**II**

C	1.267654000	-1.014568000	1.045164000
C	0.886749000	-2.376041000	1.029728000
C	1.006806000	-3.194392000	-0.200633000
C	1.589424000	-4.447477000	-0.248156000
S	1.571727000	-5.075862000	-1.876866000
C	0.770297000	-3.651680000	-2.502666000
C	0.548080000	-2.756511000	-1.494160000
C	2.243449000	-5.237243000	0.847315000
C	0.425147000	-3.525238000	-3.954972000
C	1.855165000	-0.337928000	-0.138674000
C	2.995976000	-0.751782000	-0.795280000
S	3.298691000	0.247018000	-2.193673000
C	1.895084000	1.243699000	-1.887095000
C	1.249699000	0.809627000	-0.761397000
C	3.907802000	-1.896808000	-0.478909000
C	1.549970000	2.398627000	-2.777403000
C	0.351326000	-2.971422000	2.197393000
C	0.183781000	-2.232806000	3.354499000
C	0.536740000	-0.868780000	3.400735000
C	1.076270000	-0.257252000	2.229864000
C	0.350195000	-0.106882000	4.618418000
N	0.652635000	1.146364000	4.703403000
C	0.526356000	1.876449000	5.893808000
C	0.750217000	1.318759000	7.168512000
C	0.614799000	2.105086000	8.314080000
C	0.259956000	3.454178000	8.210738000
C	0.053581000	4.018157000	6.946325000
C	0.195667000	3.241224000	5.796984000
O	1.419139000	1.029205000	2.227795000
H	3.145614000	-5.755274000	0.485008000
H	2.539195000	-4.574759000	1.673623000
H	1.569908000	-6.006525000	1.263692000
H	-0.275118000	-4.311705000	-4.283693000
H	1.320444000	-3.593452000	-4.595581000
H	-0.047386000	-2.549251000	-4.140243000

H	3.745705000	-2.233005000	0.554956000
H	3.716408000	-2.758947000	-1.139607000
H	4.966992000	-1.613204000	-0.587248000
H	1.383359000	2.080570000	-3.820556000
H	2.348663000	3.159690000	-2.791572000
H	0.629044000	2.883427000	-2.420221000
H	1.217545000	1.394576000	3.140378000
H	-0.083392000	-0.649867000	5.478323000
H	-0.240600000	-2.699956000	4.248106000
H	0.036960000	-4.015147000	2.162847000
H	0.041840000	3.671572000	4.804917000
H	1.062562000	0.275873000	7.258038000
H	-0.214414000	5.073893000	6.855029000
H	0.158417000	4.066423000	9.110016000
H	0.800268000	1.662077000	9.296139000
H	0.071207000	-1.790072000	-1.658289000
H	0.356068000	1.293162000	-0.364801000

cis-enol-**III**

C	1.391811000	0.404478000	1.049588000
C	0.239991000	-0.308307000	0.643405000
C	0.145439000	-0.961962000	-0.685903000
C	0.298660000	-0.336211000	-1.907326000
S	0.124770000	-1.483396000	-3.209584000
C	-0.168051000	-2.813111000	-2.116232000
C	-0.135095000	-2.367596000	-0.821458000
C	0.579428000	1.103070000	-2.209717000
C	-0.409231000	-4.202840000	-2.622220000
C	2.594355000	0.497478000	0.184895000
C	3.206256000	1.678237000	-0.183895000
S	4.552126000	1.370308000	-1.251892000
C	4.294570000	-0.359622000	-1.208591000
C	3.225216000	-0.652777000	-0.408055000
C	2.836435000	3.082126000	0.182320000
C	5.175058000	-1.300373000	-1.973246000
C	-0.860263000	-0.444382000	1.521388000
C	-0.836265000	0.135295000	2.776838000
C	0.290966000	0.857950000	3.216721000
C	1.416125000	0.982957000	2.347071000
C	0.305182000	1.447069000	4.540275000
N	1.321524000	2.101509000	4.996147000
C	1.363024000	2.629987000	6.294342000
C	0.754868000	2.005675000	7.401669000
C	0.841656000	2.582958000	8.669804000
C	1.535139000	3.783260000	8.857068000
C	2.156220000	4.398823000	7.764063000
C	2.081335000	3.823555000	6.495880000
O	2.511681000	1.625945000	2.747162000
H	-0.036348000	1.467709000	-3.047477000
H	0.366048000	1.723016000	-1.328185000
H	1.639138000	1.258111000	-2.470377000
H	-0.576065000	-4.885731000	-1.775778000
H	0.448568000	-4.584670000	-3.201251000
H	-1.294686000	-4.253875000	-3.278224000

H	3.066255000	3.789941000	-0.629208000
H	1.758563000	3.144448000	0.397708000
H	3.363401000	3.408142000	1.093277000
H	5.156606000	-1.092080000	-3.056454000
H	4.831943000	-2.335060000	-1.823860000
H	6.226924000	-1.240766000	-1.645783000
H	2.341103000	1.958488000	3.679504000
H	-0.612018000	1.322462000	5.144661000
H	-1.697741000	0.040168000	3.444416000
H	-1.740404000	-0.993940000	1.182664000
H	2.565845000	4.292259000	5.636439000
H	0.241552000	1.050151000	7.272630000
H	2.708607000	5.331804000	7.902366000
H	1.603062000	4.229461000	9.852123000
H	0.373008000	2.083492000	9.521843000
H	-0.283922000	-3.026397000	0.036391000
H	2.874615000	-1.672225000	-0.242222000

cis-enol-**IV**

C	1.504596000	-0.826419000	1.618403000
C	0.880476000	-2.093743000	1.556179000
C	0.964318000	-2.924696000	0.330382000
C	1.348271000	-4.252728000	0.313646000
S	1.330837000	-4.877722000	-1.316078000
C	0.796405000	-3.352059000	-1.982490000
C	0.657067000	-2.427118000	-0.985770000
C	1.780556000	-5.134141000	1.448640000
C	0.565081000	-3.180827000	-3.452778000
C	2.329236000	-0.317090000	0.494385000
C	2.093661000	0.867901000	-0.171684000
S	3.243074000	1.084844000	-1.466215000
C	4.049581000	-0.425307000	-1.113968000
C	3.443841000	-1.042660000	-0.053806000
C	1.036446000	1.894799000	0.094735000
C	5.221097000	-0.892902000	-1.922717000
C	0.140697000	-2.573869000	2.661298000
C	0.014355000	-1.811759000	3.809594000
C	0.621866000	-0.544311000	3.907419000
C	1.376190000	-0.051753000	2.799194000
C	0.483307000	0.237575000	5.119159000
N	1.028560000	1.400439000	5.258464000
C	0.943477000	2.139978000	6.446733000
C	0.924531000	1.547146000	7.725043000
C	0.846674000	2.343889000	8.868763000
C	0.790627000	3.737362000	8.760272000
C	0.826883000	4.332657000	7.493909000
C	0.913588000	3.543704000	6.347244000
O	1.981205000	1.133532000	2.862635000
H	2.635008000	-5.768401000	1.163078000
H	2.080716000	-4.525669000	2.313743000
H	0.971002000	-5.807660000	1.780326000
H	-0.229265000	-3.848562000	-3.827136000
H	1.475604000	-3.394014000	-4.037776000
H	0.264892000	-2.143605000	-3.663244000

H	1.386945000	2.640926000	0.826330000
H	0.732419000	2.420034000	-0.823853000
H	0.145139000	1.415411000	0.528545000
H	6.068758000	-0.188839000	-1.868793000
H	4.962810000	-1.013385000	-2.988552000
H	5.566751000	-1.867972000	-1.548017000
H	1.780009000	1.524642000	3.764902000
H	-0.131515000	-0.205561000	5.924016000
H	-0.570263000	-2.184941000	4.655649000
H	-0.357976000	-3.541037000	2.584907000
H	0.947350000	3.997106000	5.354089000
H	1.003943000	0.462036000	7.822010000
H	0.793434000	5.421095000	7.399562000
H	0.733067000	4.356852000	9.658474000
H	0.842006000	1.871506000	9.854618000
H	0.350160000	-1.397954000	-1.172423000
H	3.769030000	-2.009278000	0.333413000

cis-keto-**I**

C	1.220315000	-0.558359000	0.692660000
C	1.049094000	-1.942072000	0.644480000
C	1.077796000	-2.734448000	-0.610165000
C	0.352221000	-2.477065000	-1.757628000
S	0.693770000	-3.672614000	-2.982326000
C	1.802311000	-4.542224000	-1.949863000
C	1.882404000	-3.924595000	-0.731455000
C	-0.620065000	-1.376710000	-2.048137000
C	2.518493000	-5.768663000	-2.427611000
C	1.515168000	0.287151000	-0.489004000
C	2.518463000	0.056945000	-1.409697000
S	2.483042000	1.255998000	-2.679645000
C	1.129620000	2.097389000	-1.956973000
C	0.749820000	1.467592000	-0.805367000
C	3.554604000	-1.023596000	-1.443900000
C	0.555204000	3.331838000	-2.582863000
C	0.838862000	-2.699451000	1.853747000
C	0.763736000	-2.088067000	3.071949000
C	0.891646000	-0.669817000	3.181876000
C	1.118903000	0.141677000	1.977318000
C	0.806518000	-0.048339000	4.432393000
N	0.920842000	1.269716000	4.588231000
C	0.856609000	2.025160000	5.765664000
C	0.589518000	1.464640000	7.027867000
C	0.542485000	2.283106000	8.157008000
C	0.756123000	3.661636000	8.051996000
C	1.019277000	4.219684000	6.796597000
C	1.070191000	3.412183000	5.661112000
H	-0.141722000	-0.556910000	-2.608320000
H	-0.998262000	-0.947643000	-1.110540000
H	-1.476997000	-1.741074000	-2.636714000
H	3.165196000	-5.555786000	-3.295600000
H	1.816238000	-6.563571000	-2.730644000
H	3.154272000	-6.167854000	-1.623188000
H	3.667954000	-1.470297000	-0.446409000

H	3.274345000	-1.835983000	-2.134816000
H	4.534468000	-0.631256000	-1.760139000
H	0.179849000	3.142423000	-3.603008000
H	1.300240000	4.142577000	-2.654492000
H	-0.285804000	3.700082000	-1.976282000
H	1.081238000	1.730290000	3.654296000
H	0.642530000	-0.662559000	5.323783000
H	0.590875000	-2.677434000	3.978112000
H	0.708998000	-3.779668000	1.771215000
H	1.278445000	3.847873000	4.680587000
H	0.413410000	0.393522000	7.137920000
H	1.187961000	5.294861000	6.698023000
H	0.716692000	4.294854000	8.941080000
H	0.333558000	1.835228000	9.131835000
H	2.530065000	-4.285544000	0.069843000
H	-0.061153000	1.827471000	-0.172860000
O	1.231825000	1.392922000	2.053706000

cis-keto-**II**

C	1.293186000	-1.067795000	1.049287000
C	0.982645000	-2.426758000	1.034213000
C	1.081438000	-3.233982000	-0.206178000
C	1.698835000	-4.468369000	-0.290295000
S	1.626652000	-5.086594000	-1.921646000
C	0.754572000	-3.683009000	-2.498903000
C	0.552194000	-2.801641000	-1.474449000
C	2.430647000	-5.242403000	0.766906000
C	0.337737000	-3.559078000	-3.932509000
C	1.827357000	-0.345773000	-0.128635000
C	2.949996000	-0.715342000	-0.843143000
S	3.193367000	0.347030000	-2.206393000
C	1.791081000	1.313274000	-1.805727000
C	1.192505000	0.824703000	-0.677377000
C	3.896013000	-1.851060000	-0.603870000
C	1.403228000	2.502278000	-2.631252000
C	0.505105000	-3.083919000	2.223799000
C	0.327019000	-2.393434000	3.389309000
C	0.600743000	-0.993007000	3.456454000
C	1.083389000	-0.280898000	2.263587000
C	0.406838000	-0.290850000	4.651201000
N	0.640318000	1.016027000	4.757804000
C	0.497460000	1.847137000	5.875841000
C	0.024320000	1.391969000	7.119890000
C	-0.090289000	2.281619000	8.188718000
C	0.259443000	3.628039000	8.040720000
C	0.728814000	4.081229000	6.803384000
C	0.848373000	3.201901000	5.728003000
H	3.337909000	-5.716918000	0.360303000
H	2.734557000	-4.578221000	1.588616000
H	1.808288000	-6.044936000	1.200058000
H	-0.355432000	-4.363156000	-4.232620000
H	1.203116000	-3.599344000	-4.615232000
H	-0.169778000	-2.595735000	-4.089232000
H	3.793986000	-2.214579000	0.428474000

H	3.686094000	-2.701869000	-1.273759000
H	4.942876000	-1.545088000	-0.760788000
H	1.198437000	2.228485000	-3.680310000
H	2.194763000	3.271019000	-2.643228000
H	0.493225000	2.962573000	-2.217897000
H	0.975759000	1.398299000	3.833543000
H	0.051653000	-0.828295000	5.536573000
H	-0.046752000	-2.903566000	4.282879000
H	0.250515000	-4.142674000	2.166730000
H	1.216817000	3.555499000	4.761657000
H	-0.258367000	0.347849000	7.262491000
H	1.006034000	5.130051000	6.671891000
H	0.166265000	4.317479000	8.882734000
H	-0.459518000	1.915442000	9.150028000
H	0.040114000	-1.848515000	-1.607490000
H	0.314593000	1.284174000	-0.222863000
O	1.313571000	0.956543000	2.292859000

cis-keto-**III**

C	1.364489000	0.370297000	1.042267000
C	0.269215000	-0.402216000	0.656590000
C	0.170769000	-1.034320000	-0.681939000
C	0.271360000	-0.380383000	-1.894877000
S	0.105818000	-1.508125000	-3.214972000
C	-0.112610000	-2.868279000	-2.141319000
C	-0.065806000	-2.446299000	-0.839455000
C	0.493561000	1.072819000	-2.176494000
C	-0.314292000	-4.256196000	-2.669026000
C	2.550885000	0.544588000	0.171578000
C	3.111560000	1.761435000	-0.161877000
S	4.464517000	1.545651000	-1.244202000
C	4.282494000	-0.194219000	-1.254443000
C	3.230185000	-0.557225000	-0.459998000
C	2.684853000	3.137473000	0.246049000
C	5.201918000	-1.072704000	-2.047003000
C	-0.823769000	-0.644759000	1.560430000
C	-0.833882000	-0.102099000	2.814330000
C	0.251901000	0.709526000	3.263655000
C	1.401762000	0.960609000	2.380685000
C	0.235630000	1.254014000	4.553061000
N	1.232843000	1.996736000	5.029298000
C	1.351832000	2.609811000	6.282917000
C	0.323756000	2.606709000	7.242889000
C	0.517963000	3.237173000	8.472473000
C	1.725610000	3.879905000	8.764944000
C	2.746318000	3.887459000	7.808487000
C	2.565233000	3.258794000	6.577231000
H	-0.131209000	1.422615000	-3.013798000
H	0.251319000	1.670974000	-1.287345000
H	1.548071000	1.274510000	-2.426535000
H	-0.439482000	-4.960256000	-1.832785000
H	0.544734000	-4.596088000	-3.271944000
H	-1.210403000	-4.326995000	-3.308575000
H	2.935941000	3.887567000	-0.519986000

H	1.595781000	3.162479000	0.407318000
H	3.152261000	3.427024000	1.200035000
H	5.177547000	-0.830161000	-3.123050000
H	4.901013000	-2.124965000	-1.932846000
H	6.250157000	-0.981604000	-1.714685000
H	1.994604000	2.081598000	4.304519000
H	-0.623525000	1.061319000	5.203949000
H	-1.678345000	-0.278402000	3.488295000
H	-1.661131000	-1.251073000	1.210664000
H	3.364061000	3.261115000	5.831264000
H	-0.631799000	2.122063000	7.036656000
H	3.694923000	4.386326000	8.021642000
H	1.868409000	4.372276000	9.729400000
H	-0.288858000	3.228296000	9.209680000
H	-0.171367000	-3.127147000	0.007567000
H	2.927319000	-1.596096000	-0.322478000
O	2.382873000	1.643499000	2.777476000

cis-keto-**IV**

C	1.500259000	-0.875437000	1.625457000
C	0.974115000	-2.165926000	1.585778000
C	1.050789000	-2.989960000	0.353894000
C	1.511653000	-4.292803000	0.311572000
S	1.455273000	-4.913256000	-1.318998000
C	0.798308000	-3.420617000	-1.950771000
C	0.652219000	-2.508361000	-0.943622000
C	2.052887000	-5.148738000	1.419412000
C	0.486728000	-3.260253000	-3.407395000
C	2.251725000	-0.304036000	0.482750000
C	1.942371000	0.887854000	-0.140718000
S	3.034283000	1.191898000	-1.467380000
C	3.919062000	-0.289155000	-1.187247000
C	3.378217000	-0.961530000	-0.125603000
C	0.851352000	1.859841000	0.188127000
C	5.082443000	-0.680511000	-2.046786000
C	0.305877000	-2.727150000	2.729060000
C	0.163443000	-2.008124000	3.883121000
C	0.678543000	-0.680166000	3.981865000
C	1.373875000	-0.070508000	2.837323000
C	0.530557000	0.046289000	5.169592000
N	1.003381000	1.282270000	5.313158000
C	0.929695000	2.129069000	6.426266000
C	0.236823000	1.795677000	7.604109000
C	0.210406000	2.691987000	8.673187000
C	0.864062000	3.926024000	8.589487000
C	1.550046000	4.259356000	7.416887000
C	1.585231000	3.371410000	6.342488000
O	1.849063000	1.094300000	2.913633000
H	2.933359000	-5.723526000	1.090223000
H	2.353386000	-4.526432000	2.274295000
H	1.305731000	-5.876222000	1.782346000
H	-0.283638000	-3.973319000	-3.746784000
H	1.379145000	-3.417313000	-4.036430000
H	0.115641000	-2.242286000	-3.597849000

H	1.178154000	2.559537000	0.973673000
H	0.532143000	2.434796000	-0.694904000
H	-0.024967000	1.323790000	0.585167000
H	5.484486000	-1.648253000	-1.711077000
H	5.899154000	0.059989000	-2.001185000
H	4.794519000	-0.784773000	-3.106870000
H	1.485522000	1.586415000	4.423843000
H	0.012350000	-0.413449000	6.017625000
H	-0.359179000	-2.439073000	4.743104000
H	-0.116759000	-3.729147000	2.645485000
H	2.123713000	3.630188000	5.427260000
H	-0.288004000	0.843260000	7.693209000
H	2.064807000	5.219889000	7.335990000
H	0.837212000	4.622034000	9.430825000
H	-0.331822000	2.421428000	9.582747000
H	0.277344000	-1.498163000	-1.108507000
H	3.761531000	-1.921371000	0.223628000

trans-enol-**I**

C	1.377651000	0.362115000	0.374463000
C	1.031604000	-0.990920000	0.606635000
C	0.961208000	-2.015568000	-0.465080000
C	0.249443000	-1.925668000	-1.645243000
S	0.465096000	-3.367483000	-2.604790000
C	1.498804000	-4.113753000	-1.410968000
C	1.651564000	-3.274802000	-0.340636000
C	-0.626928000	-0.822807000	-2.152511000
C	2.089904000	-5.473507000	-1.627714000
C	1.717419000	0.900168000	-0.969764000
C	2.677745000	0.382284000	-1.814584000
S	2.711356000	1.260111000	-3.323927000
C	1.446371000	2.353391000	-2.811330000
C	1.038614000	2.032785000	-1.546560000
C	3.613761000	-0.767501000	-1.603886000
C	0.955578000	3.452431000	-3.703909000
C	0.769416000	-1.424235000	1.927832000
C	0.806366000	-0.544416000	2.992183000
C	1.117175000	0.815678000	2.793120000
C	1.417612000	1.250029000	1.480613000
C	1.109724000	1.744901000	3.922930000
N	0.959460000	1.372520000	5.138041000
C	0.865157000	2.302415000	6.177764000
C	1.478623000	1.986748000	7.406243000
C	1.422120000	2.879105000	8.475757000
C	0.727268000	4.088966000	8.353037000
C	0.087520000	4.396576000	7.148267000
C	0.153679000	3.515386000	6.066694000
O	1.776680000	2.531051000	1.208637000
H	-0.098606000	-0.194562000	-2.887747000
H	-0.930706000	-0.168296000	-1.324640000
H	-1.536018000	-1.219708000	-2.631772000
H	2.737410000	-5.503352000	-2.520288000
H	1.311708000	-6.243555000	-1.762621000
H	2.700510000	-5.758803000	-0.758052000

H	3.686640000	-1.005304000	-0.533812000
H	3.260102000	-1.676656000	-2.117410000
H	4.624857000	-0.537163000	-1.976122000
H	0.540741000	3.062464000	-4.648848000
H	1.760583000	4.159168000	-3.967668000
H	0.161708000	4.019859000	-3.195463000
H	1.913913000	3.026098000	2.025486000
H	1.230702000	2.825743000	3.692910000
H	0.581481000	-0.871173000	4.009032000
H	0.502404000	-2.469582000	2.094033000
H	-0.378911000	3.745802000	5.140808000
H	2.005518000	1.034008000	7.490891000
H	-0.477966000	5.327285000	7.051232000
H	0.671907000	4.780260000	9.197452000
H	1.915767000	2.625650000	9.417637000
H	2.271760000	-3.525760000	0.521978000
H	0.262106000	2.586491000	-1.017742000

trans-enol-**II**

C	1.518076000	-0.778397000	0.805862000
C	1.069863000	-2.110346000	0.966049000
C	1.056974000	-3.061314000	-0.170683000
C	1.577527000	-4.341382000	-0.117593000
S	1.409574000	-5.140971000	-1.660238000
C	0.628625000	-3.753006000	-2.384934000
C	0.522733000	-2.743092000	-1.470122000
C	2.273158000	-5.041800000	1.012477000
C	0.181262000	-3.767192000	-3.814635000
C	2.042836000	-0.271521000	-0.488575000
C	3.116635000	-0.813547000	-1.164000000
S	3.352338000	-0.007751000	-2.693498000
C	2.011351000	1.080044000	-2.420550000
C	1.433597000	0.815331000	-1.208485000
C	4.001630000	-1.956098000	-0.771693000
C	1.642549000	2.129810000	-3.424396000
C	0.607391000	-2.538723000	2.231186000
C	0.568493000	-1.670543000	3.306832000
C	0.985758000	-0.330736000	3.173869000
C	1.467145000	0.100346000	1.913836000
C	0.899697000	0.582888000	4.312764000
N	0.570505000	0.208888000	5.491526000
C	0.414623000	1.131109000	6.530668000
C	0.846512000	0.757380000	7.818665000
C	0.721424000	1.639322000	8.890987000
C	0.135493000	2.898281000	8.708302000
C	-0.325935000	3.265631000	7.440445000
C	-0.189014000	2.394231000	6.357576000
O	1.917246000	1.362083000	1.696028000
H	3.113697000	-5.653869000	0.648823000
H	2.669620000	-4.310424000	1.731658000
H	1.593919000	-5.714280000	1.564767000
H	-0.575807000	-4.547035000	-4.003283000
H	1.021887000	-3.951314000	-4.504602000
H	-0.260475000	-2.793860000	-4.074800000

H	3.896992000	-2.162161000	0.302892000
H	3.729811000	-2.878371000	-1.311925000
H	5.061542000	-1.738914000	-0.980114000
H	1.388924000	1.692294000	-4.404794000
H	2.464333000	2.847136000	-3.589911000
H	0.767751000	2.695331000	-3.069945000
H	1.953725000	1.859104000	2.522379000
H	1.129050000	1.654101000	4.123513000
H	0.195856000	-1.990713000	4.281535000
H	0.241165000	-3.560333000	2.342095000
H	-0.584626000	2.673551000	5.378039000
H	1.289410000	-0.232346000	7.948246000
H	-0.806951000	4.236415000	7.293403000
H	0.025231000	3.582178000	9.553357000
H	1.074857000	1.339680000	9.881126000
H	0.077893000	-1.776824000	-1.708449000
H	0.584639000	1.379506000	-0.819600000

trans-enol-**III**

C	1.717428000	0.456822000	1.051839000
C	0.494063000	-0.200347000	0.778937000
C	0.218537000	-0.845220000	-0.528762000
C	0.257888000	-0.223505000	-1.761172000
S	-0.113321000	-1.358516000	-3.032304000
C	-0.336900000	-2.677076000	-1.909325000
C	-0.136341000	-2.236982000	-0.627900000
C	0.562409000	1.203749000	-2.096320000
C	-0.696183000	-4.053229000	-2.381213000
C	2.815500000	0.493899000	0.053149000
C	3.430575000	1.645705000	-0.393376000
S	4.625204000	1.277236000	-1.611224000
C	4.298014000	-0.439512000	-1.526536000
C	3.319198000	-0.684104000	-0.603291000
C	3.169085000	3.064548000	0.007492000
C	5.037410000	-1.418815000	-2.386271000
C	-0.493238000	-0.289898000	1.784017000
C	-0.300963000	0.293047000	3.023735000
C	0.895048000	0.974794000	3.322665000
C	1.910193000	1.021535000	2.336305000
C	1.065464000	1.630421000	4.619778000
N	0.236893000	1.502145000	5.586630000
C	0.386626000	2.226142000	6.773332000
C	0.063567000	1.584392000	7.985340000
C	0.199340000	2.255213000	9.199789000
C	0.629322000	3.587852000	9.228745000
C	0.921166000	4.243632000	8.028658000
C	0.803782000	3.573197000	6.809084000
O	3.123258000	1.584207000	2.579055000
H	-0.132255000	1.597721000	-2.855386000
H	0.480729000	1.828650000	-1.196382000
H	1.589213000	1.315257000	-2.481377000
H	-0.792313000	-4.731084000	-1.519908000
H	0.070148000	-4.470015000	-3.056393000
H	-1.653920000	-4.063175000	-2.928528000

H	3.311329000	3.759916000	-0.834244000
H	2.134285000	3.169988000	0.368295000
H	3.830429000	3.376367000	0.832118000
H	4.896676000	-1.210415000	-3.460357000
H	4.669848000	-2.437125000	-2.190226000
H	6.122758000	-1.405360000	-2.188942000
H	3.228212000	1.765834000	3.520984000
H	1.957716000	2.281596000	4.742979000
H	-1.070815000	0.255032000	3.796614000
H	-1.430611000	-0.801792000	1.557026000
H	0.998748000	4.102863000	5.873429000
H	-0.283032000	0.549597000	7.945588000
H	1.233756000	5.291286000	8.039716000
H	0.720811000	4.116326000	10.180767000
H	-0.041585000	1.738448000	10.132513000
H	-0.215273000	-2.890662000	0.243039000
H	2.944292000	-1.686315000	-0.392114000

trans-enol-**IV**

C	1.816689000	-0.438433000	1.536770000
C	1.068805000	-1.635738000	1.634764000
C	0.979965000	-2.576280000	0.491947000
C	1.231559000	-3.933080000	0.578639000
S	1.032568000	-4.696252000	-0.978390000
C	0.600545000	-3.189612000	-1.753214000
C	0.625232000	-2.169708000	-0.843394000
C	1.657800000	-4.749251000	1.763555000
C	0.277754000	-3.128045000	-3.214943000
C	2.602021000	-0.122840000	0.316604000
C	2.428513000	1.009620000	-0.451816000
S	3.490429000	0.982712000	-1.835768000
C	4.175659000	-0.560275000	-1.382760000
C	3.597670000	-1.007679000	-0.226158000
C	1.496138000	2.159148000	-0.222186000
C	5.230330000	-1.221733000	-2.216583000
C	0.381670000	-1.931829000	2.831708000
C	0.411806000	-1.057010000	3.903925000
C	1.132527000	0.150918000	3.832777000
C	1.851675000	0.437600000	2.645863000
C	1.110760000	1.089515000	4.954847000
N	0.584066000	0.817960000	6.089189000
C	0.499060000	1.779925000	7.100198000
C	0.694968000	1.361034000	8.431100000
C	0.627983000	2.277897000	9.479068000
C	0.333698000	3.623516000	9.225385000
C	0.105453000	4.042639000	7.910989000
C	0.187345000	3.133489000	6.853942000
O	2.621107000	1.550308000	2.516991000
H	2.415973000	-5.498453000	1.484859000
H	2.086928000	-4.101459000	2.541416000
H	0.810963000	-5.294577000	2.215580000
H	-0.602789000	-3.741566000	-3.470251000
H	1.117307000	-3.485255000	-3.834858000
H	0.063581000	-2.088615000	-3.504758000

H	1.978405000	2.947472000	0.379127000
H	1.152677000	2.605761000	-1.168051000
H	0.611717000	1.821059000	0.339762000
H	6.141284000	-0.604797000	-2.297700000
H	4.877281000	-1.421140000	-3.242596000
H	5.512443000	-2.184468000	-1.764837000
H	2.722932000	1.991722000	3.369065000
H	1.567564000	2.088783000	4.786751000
H	-0.136520000	-1.267127000	4.824071000
H	-0.210196000	-2.846737000	2.889209000
H	-0.027867000	3.460778000	5.833865000
H	0.910524000	0.306302000	8.614360000
H	-0.149219000	5.085944000	7.705893000
H	0.267160000	4.337521000	10.049861000
H	0.796837000	1.939508000	10.504759000
H	0.404843000	-1.135547000	-1.108348000
H	3.857329000	-1.961166000	0.235722000

trans-keto-**I**

C	1.300934000	-0.997432000	0.518379000
C	1.136105000	-2.369454000	0.404430000
C	1.108104000	-3.102402000	-0.885617000
C	0.331318000	-2.790124000	-1.985143000
S	0.612627000	-3.926394000	-3.279534000
C	1.764293000	-4.845437000	-2.341538000
C	1.902383000	-4.286408000	-1.100171000
C	-0.646599000	-1.673758000	-2.178441000
C	2.454325000	-6.048099000	-2.909717000
C	1.550984000	-0.088913000	-0.623451000
C	2.514663000	-0.271291000	-1.597052000
S	2.438054000	0.999630000	-2.791963000
C	1.120324000	1.804513000	-1.968370000
C	0.782817000	1.112624000	-0.839620000
C	3.542565000	-1.351297000	-1.735200000
C	0.531356000	3.076394000	-2.498336000
C	0.979621000	-3.195282000	1.588907000
C	0.951460000	-2.661947000	2.839690000
C	1.072053000	-1.245307000	3.034017000
C	1.234071000	-0.348447000	1.855348000
C	1.047990000	-0.616253000	4.263428000
N	0.903522000	-1.203913000	5.467839000
C	0.882090000	-0.589582000	6.732024000
C	0.689888000	-1.405036000	7.861899000
C	0.660412000	-0.847070000	9.139621000
C	0.821059000	0.530908000	9.313762000
C	1.012627000	1.341793000	8.190334000
C	1.045130000	0.796143000	6.906334000
H	-0.184280000	-0.823306000	-2.705528000
H	-0.990825000	-1.298524000	-1.205179000
H	-1.524064000	-2.001785000	-2.757924000
H	1.736645000	-6.826074000	-3.220392000
H	3.123417000	-6.487347000	-2.154687000
H	3.063125000	-5.793615000	-3.793764000
H	3.701228000	-1.848845000	-0.768408000

H	3.225856000	-2.126828000	-2.452317000
H	4.508038000	-0.945713000	-2.077699000
H	0.110376000	2.947701000	-3.510138000
H	1.280553000	3.884307000	-2.556436000
H	-0.278560000	3.415761000	-1.835345000
H	0.793019000	-2.213067000	5.481840000
H	1.154307000	0.471107000	4.246227000
H	0.821697000	-3.330016000	3.698146000
H	0.855853000	-4.270612000	1.449071000
H	1.199086000	1.452586000	6.049646000
H	0.561991000	-2.484323000	7.734866000
H	1.140951000	2.420405000	8.310422000
H	0.797900000	0.968651000	10.313999000
H	0.509882000	-1.497368000	10.004859000
H	2.585854000	-4.686328000	-0.348519000
H	0.002719000	1.441312000	-0.154129000
O	1.315093000	0.875384000	1.987229000

trans-keto-**II**

C	1.082924000	-0.842747000	0.337641000
C	0.770511000	-2.177027000	0.541627000
C	1.008058000	-3.209812000	-0.496531000
C	1.612532000	-4.431487000	-0.264735000
S	1.723651000	-5.357676000	-1.740837000
C	0.947700000	-4.100597000	-2.679988000
C	0.639057000	-3.037787000	-1.878447000
C	2.204971000	-4.975642000	1.002394000
C	0.704072000	-4.263361000	-4.149167000
C	1.772365000	-0.346037000	-0.872826000
C	2.967633000	-0.837239000	-1.363423000
S	3.404237000	-0.032350000	-2.848308000
C	1.984720000	0.989898000	-2.805683000
C	1.239070000	0.710685000	-1.694230000
C	3.855718000	-1.906889000	-0.807554000
C	1.731590000	2.014557000	-3.869376000
C	0.135781000	-2.605825000	1.773613000
C	-0.185547000	-1.722246000	2.757721000
C	0.081950000	-0.320318000	2.602824000
C	0.708707000	0.179156000	1.346020000
C	-0.211817000	0.644781000	3.546388000
N	-0.782590000	0.448831000	4.751463000
C	-1.089203000	1.411064000	5.729386000
C	-1.701648000	0.975508000	6.918361000
C	-2.027035000	1.888164000	7.921336000
C	-1.748643000	3.248522000	7.757455000
C	-1.139452000	3.681671000	6.575248000
C	-0.808295000	2.778867000	5.563960000
H	3.150925000	-5.505403000	0.807075000
H	2.414909000	-4.160842000	1.710085000
H	1.529073000	-5.689488000	1.505181000
H	0.039409000	-5.116795000	-4.365830000
H	1.642963000	-4.428970000	-4.703821000
H	0.232372000	-3.353980000	-4.549994000
H	3.626243000	-2.073155000	0.254490000

H	3.709229000	-2.867828000	-1.328989000
H	4.919498000	-1.632756000	-0.892724000
H	1.652115000	1.559882000	-4.871588000
H	2.536991000	2.767412000	-3.915184000
H	0.789028000	2.542685000	-3.661135000
H	-1.028819000	-0.504534000	4.999010000
H	0.047137000	1.671334000	3.275656000
H	-0.672713000	-2.095168000	3.665327000
H	-0.113578000	-3.662073000	1.882541000
H	-0.332856000	3.145447000	4.653955000
H	-1.923890000	-0.087204000	7.054795000
H	-0.915032000	4.741676000	6.432745000
H	-2.003246000	3.963889000	8.542347000
H	-2.502547000	1.530135000	8.837786000
H	0.160900000	-2.132895000	-2.253986000
H	0.324370000	1.246220000	-1.441087000
O	0.909833000	1.381557000	1.157472000

trans-keto-**III**

C	1.296283000	0.129273000	1.138555000
C	0.155805000	-0.541142000	0.725979000
C	-0.010984000	-1.081696000	-0.645090000
C	0.120307000	-0.362744000	-1.817929000
S	-0.150367000	-1.390542000	-3.200673000
C	-0.455946000	-2.794614000	-2.207795000
C	-0.357306000	-2.459324000	-0.883806000
C	0.451586000	1.083507000	-2.014033000
C	-0.770589000	-4.127879000	-2.815152000
C	2.484915000	0.274889000	0.268260000
C	3.135220000	1.466181000	0.010882000
S	4.458924000	1.221912000	-1.100582000
C	4.145107000	-0.494597000	-1.225419000
C	3.073497000	-0.829695000	-0.444995000
C	2.820809000	2.841973000	0.512367000
C	4.991433000	-1.384710000	-2.083650000
C	-0.947539000	-0.768427000	1.638049000
C	-0.920445000	-0.306794000	2.918015000
C	0.218187000	0.413320000	3.412759000
C	1.394241000	0.647427000	2.526077000
C	0.328086000	0.921306000	4.692918000
N	-0.593354000	0.846462000	5.673287000
C	-0.506512000	1.353830000	6.981537000
C	-1.610148000	1.176754000	7.835355000
C	-1.576942000	1.660354000	9.142957000
C	-0.445650000	2.328063000	9.621771000
C	0.652008000	2.503790000	8.772881000
C	0.632050000	2.024201000	7.462528000
H	-0.142959000	1.528288000	-2.827834000
H	0.254382000	1.644728000	-1.090178000
H	1.519074000	1.219378000	-2.253544000
H	-0.936430000	-4.871436000	-2.021232000
H	0.050951000	-4.492944000	-3.454460000
H	-1.678902000	-4.091163000	-3.440188000
H	3.132834000	3.618177000	-0.203675000

H	1.737411000	2.945282000	0.679868000
H	3.309040000	3.026441000	1.481567000
H	4.981465000	-1.068665000	-3.140693000
H	4.611625000	-2.416448000	-2.037679000
H	6.044840000	-1.396072000	-1.755432000
H	-1.464342000	0.371756000	5.456699000
H	1.263866000	1.436363000	4.923247000
H	-1.785013000	-0.488085000	3.565977000
H	-1.822477000	-1.301221000	1.260657000
H	1.503399000	2.173748000	6.824618000
H	-2.500019000	0.656148000	7.468687000
H	1.543814000	3.022663000	9.133066000
H	-0.418895000	2.707179000	10.645578000
H	-2.444453000	1.512897000	9.790914000
H	-0.500990000	-3.182899000	-0.078736000
H	2.694469000	-1.849960000	-0.372601000
O	2.401106000	1.230090000	2.937299000

trans-keto-**IV**

C	1.966580000	-1.218380000	1.497062000
C	1.310450000	-2.430851000	1.365163000
C	1.151962000	-3.090558000	0.044833000
C	1.459914000	-4.413616000	-0.211693000
S	1.142189000	-4.807369000	-1.882332000
C	0.581513000	-3.189868000	-2.240901000
C	0.656496000	-2.407950000	-1.122451000
C	2.034968000	-5.452478000	0.706601000
C	0.118936000	-2.811625000	-3.614757000
C	2.645995000	-0.568786000	0.352927000
C	2.416851000	0.727061000	-0.065805000
S	3.381596000	1.100241000	-1.470562000
C	4.106292000	-0.489988000	-1.499865000
C	3.609076000	-1.247004000	-0.474852000
C	1.495370000	1.754408000	0.517150000
C	5.113055000	-0.872787000	-2.541602000
C	0.713067000	-3.081556000	2.513716000
C	0.770297000	-2.526111000	3.755478000
C	1.431809000	-1.270272000	3.968015000
C	2.065247000	-0.558695000	2.820377000
C	1.536695000	-0.636906000	5.191605000
N	1.050292000	-1.071380000	6.370515000
C	1.141034000	-0.445978000	7.626466000
C	0.547138000	-1.085716000	8.729324000
C	0.607801000	-0.509277000	9.997729000
C	1.260038000	0.712575000	10.188981000
C	1.850726000	1.348695000	9.092257000
C	1.797419000	0.782660000	7.817825000
O	2.649992000	0.517270000	2.973686000
H	2.808648000	-6.052323000	0.201019000
H	2.495716000	-4.977555000	1.584456000
H	1.264881000	-6.154962000	1.071091000
H	-0.756114000	-3.402670000	-3.934139000
H	0.909888000	-2.962569000	-4.368663000
H	-0.163835000	-1.748627000	-3.631823000

H	1.989059000	2.296431000	1.338859000
H	1.156245000	2.478001000	-0.240324000
H	0.607382000	1.266005000	0.947785000
H	6.016226000	-0.240520000	-2.497055000
H	4.703108000	-0.785678000	-3.562329000
H	5.424214000	-1.917692000	-2.392406000
H	0.553650000	-1.956965000	6.369914000
H	2.072539000	0.315509000	5.190791000
H	0.290299000	-3.046876000	4.591301000
H	0.181741000	-4.020950000	2.353903000
H	2.267640000	1.301034000	6.981932000
H	0.034359000	-2.042083000	8.588692000
H	2.364420000	2.303934000	9.226065000
H	1.307997000	1.164671000	11.181923000
H	0.140244000	-1.021748000	10.842076000
H	0.377386000	-1.354195000	-1.115224000
H	3.910990000	-2.281240000	-0.303722000

cis-enol-**C**

C	1.275777000	-0.499483000	0.750562000
C	1.338755000	-1.980265000	0.739006000
C	1.311587000	-2.655140000	-0.465283000
C	1.070267000	-1.875904000	-1.760759000
S	1.614290000	-3.033159000	-3.133524000
C	1.596383000	-4.413377000	-2.022766000
C	1.437012000	-4.063673000	-0.715951000
C	-0.450891000	-1.646637000	-1.924674000
C	1.757833000	-5.790265000	-2.581060000
C	1.442828000	0.204279000	-0.437996000
C	1.885732000	-0.578433000	-1.685116000
S	1.587487000	0.573776000	-3.130312000
C	1.383702000	1.939341000	-2.042103000
C	1.324974000	1.602178000	-0.717743000
C	3.416031000	-0.801412000	-1.590547000
C	1.283904000	3.316695000	-2.613917000
C	1.398483000	-2.677204000	2.007021000
C	1.276919000	-2.011009000	3.182406000
C	1.089408000	-0.579266000	3.233696000
C	1.077932000	0.160461000	2.031657000
C	0.928058000	0.093237000	4.489228000
N	0.749874000	1.382068000	4.571044000
C	0.649988000	2.069880000	5.784255000
C	1.284260000	1.650997000	6.972808000
C	1.143475000	2.392031000	8.147164000
C	0.379811000	3.564190000	8.162305000
C	-0.237119000	3.996765000	6.982552000
C	-0.098751000	3.263505000	5.804711000
O	0.912416000	1.469301000	2.061818000
H	-0.681272000	-1.147285000	-2.875165000
H	-0.834373000	-1.028636000	-1.098806000
H	-0.964542000	-2.617565000	-1.898568000
H	2.698105000	-5.881375000	-3.152150000
H	0.936910000	-6.035554000	-3.277006000
H	1.767469000	-6.539291000	-1.775602000

H	3.658044000	-1.408102000	-0.706161000
H	3.794051000	-1.316934000	-2.483023000
H	3.918313000	0.171313000	-1.496349000
H	0.453608000	3.388000000	-3.337325000
H	2.205857000	3.584696000	-3.158153000
H	1.118675000	4.057058000	-1.818116000
H	0.800838000	1.735108000	3.054430000
H	0.942779000	-0.534563000	5.397339000
H	1.307181000	-2.557668000	4.129542000
H	1.517640000	-3.761091000	2.008352000
H	-0.578119000	3.592739000	4.879893000
H	1.914239000	0.758819000	6.968403000
H	-0.829688000	4.915439000	6.980010000
H	0.276483000	4.142659000	9.083465000
H	1.647443000	2.056705000	9.057680000
H	1.438874000	-4.815651000	0.074613000
H	1.162220000	2.342923000	0.059864000

cis-keto-**C**

C	1.286019000	-0.495062000	0.706242000
C	1.391511000	-1.971057000	0.707669000
C	1.342140000	-2.660834000	-0.484693000
C	1.043073000	-1.904303000	-1.782608000
S	1.577261000	-3.063469000	-3.157929000
C	1.639975000	-4.429255000	-2.029944000
C	1.504838000	-4.069637000	-0.724370000
C	-0.485443000	-1.712503000	-1.908551000
C	1.836526000	-5.806181000	-2.577289000
C	1.405747000	0.193380000	-0.488374000
C	1.828468000	-0.584161000	-1.744701000
S	1.455097000	0.552898000	-3.184744000
C	1.238119000	1.916445000	-2.094567000
C	1.237198000	1.588895000	-0.768581000
C	3.364647000	-0.769345000	-1.700933000
C	1.065328000	3.284739000	-2.671562000
C	1.521874000	-2.649484000	1.981364000
C	1.427919000	-1.982959000	3.158207000
C	1.187823000	-0.557593000	3.230638000
C	1.091694000	0.226913000	1.987976000
C	1.059331000	0.059032000	4.466121000
N	0.832795000	1.375195000	4.647600000
C	0.692101000	2.075806000	5.846389000
C	0.777041000	1.466691000	7.113266000
C	0.625281000	2.233293000	8.269628000
C	0.388256000	3.609538000	8.194992000
C	0.303780000	4.216967000	6.937220000
C	0.453198000	3.462926000	5.774805000
H	-0.751609000	-1.235140000	-2.861247000
H	-0.859705000	-1.088247000	-1.083168000
H	-0.977381000	-2.693414000	-1.851966000
H	2.765088000	-5.870322000	-3.170759000
H	1.007928000	-6.088756000	-3.249549000
H	1.893111000	-6.544865000	-1.764204000
H	3.648454000	-1.371495000	-0.825898000

H	3.724352000	-1.275438000	-2.606383000
H	3.848130000	0.214263000	-1.621334000
H	0.202732000	3.319704000	-3.358719000
H	1.953185000	3.578994000	-3.257042000
H	0.908222000	4.025038000	-1.874196000
H	0.765282000	1.880780000	3.750141000
H	1.145355000	-0.555965000	5.366191000
H	1.518337000	-2.533523000	4.100162000
H	1.677587000	-3.728981000	1.987891000
H	0.385865000	3.942658000	4.794659000
H	0.961550000	0.395611000	7.206094000
H	0.119077000	5.291375000	6.858055000
H	0.270916000	4.201166000	9.105529000
H	0.694276000	1.743326000	9.244412000
H	1.554083000	-4.811268000	0.074383000
H	1.072361000	2.316022000	0.022055000
O	0.882368000	1.456854000	2.027241000

trans-enol-**C**

C	1.384484000	-0.195770000	0.533376000
C	1.399308000	-1.667566000	0.711508000
C	1.318607000	-2.495154000	-0.390535000
C	1.071360000	-1.885562000	-1.771123000
S	1.539328000	-3.233118000	-2.989738000
C	1.506603000	-4.452060000	-1.705426000
C	1.392636000	-3.927673000	-0.453124000
C	-0.444891000	-1.622768000	-1.932202000
C	1.609268000	-5.895656000	-2.078207000
C	1.547661000	0.339198000	-0.742573000
C	1.933913000	-0.622407000	-1.881960000
S	1.642975000	0.329962000	-3.467291000
C	1.516929000	1.839241000	-2.575019000
C	1.475438000	1.688741000	-1.216736000
C	3.457090000	-0.888946000	-1.786021000
C	1.457960000	3.128060000	-3.329907000
C	1.475030000	-2.194654000	2.057086000
C	1.392997000	-1.383941000	3.138829000
C	1.229765000	0.048908000	3.002763000
C	1.237775000	0.613997000	1.730825000
C	1.063613000	0.858885000	4.196013000
N	1.133404000	0.383771000	5.387440000
C	0.877890000	1.180707000	6.503878000
C	1.650713000	0.966516000	7.663687000
C	1.443151000	1.735350000	8.807582000
C	0.441938000	2.714755000	8.832086000
C	-0.351832000	2.914134000	7.698201000
C	-0.140101000	2.158166000	6.543125000
O	1.139651000	1.950407000	1.537001000
H	-0.681806000	-1.242969000	-2.934861000
H	-0.785490000	-0.888390000	-1.186827000
H	-0.990900000	-2.562053000	-1.768428000
H	0.763139000	-6.201431000	-2.717649000
H	1.615444000	-6.531059000	-1.180443000
H	2.531101000	-6.094516000	-2.651864000

H	3.699785000	-1.376452000	-0.830878000
H	3.796238000	-1.535680000	-2.605791000
H	3.994714000	0.067998000	-1.836984000
H	0.614996000	3.134047000	-4.042191000
H	2.376961000	3.281060000	-3.921633000
H	1.339131000	3.977579000	-2.641981000
H	1.135109000	2.409962000	2.386897000
H	0.862642000	1.944851000	4.060172000
H	1.427495000	-1.778665000	4.155116000
H	1.572824000	-3.272065000	2.196061000
H	-0.790608000	2.294213000	5.675670000
H	2.419936000	0.191902000	7.634089000
H	-1.152721000	3.658460000	7.715500000
H	0.271815000	3.306833000	9.734612000
H	2.061590000	1.564361000	9.692812000
H	1.390840000	-4.568010000	0.430142000
H	1.359582000	2.541642000	-0.555089000

trans-keto-**C**

C	1.229217000	-0.444627000	0.211665000
C	1.320230000	-1.920472000	0.235450000
C	1.291284000	-2.628992000	-0.946594000
C	1.027520000	-1.887614000	-2.260884000
S	1.577767000	-3.071390000	-3.608796000
C	1.610198000	-4.419740000	-2.461008000
C	1.449653000	-4.041936000	-1.163210000
C	-0.496069000	-1.683435000	-2.421260000
C	1.811737000	-5.805575000	-2.983750000
C	1.384618000	0.223207000	-0.987227000
C	1.824906000	-0.573642000	-2.225349000
S	1.489574000	0.548763000	-3.685845000
C	1.264490000	1.929032000	-2.615090000
C	1.236741000	1.618572000	-1.286544000
C	3.357343000	-0.773730000	-2.149937000
C	1.113722000	3.290687000	-3.213563000
C	1.416895000	-2.583010000	1.519679000
C	1.304195000	-1.905509000	2.690326000
C	1.070775000	-0.479649000	2.745073000
C	1.007151000	0.305731000	1.481136000
C	0.902243000	0.238544000	3.905157000
N	0.919128000	-0.256827000	5.169679000
C	0.760531000	0.446541000	6.369982000
C	0.796786000	-0.276742000	7.578012000
C	0.643730000	0.375080000	8.800731000
C	0.451053000	1.759637000	8.847655000
C	0.415095000	2.479799000	7.649366000
C	0.567698000	1.840094000	6.418386000
H	-0.736075000	-1.215990000	-3.385697000
H	-0.880640000	-1.043628000	-1.612861000
H	-0.999613000	-2.658349000	-2.362191000
H	2.752567000	-5.882216000	-3.555881000
H	0.996499000	-6.093545000	-3.669801000
H	1.846952000	-6.532772000	-2.159218000
H	3.618081000	-1.370610000	-1.264004000

H	3.730307000	-1.290776000	-3.043846000
H	3.849049000	0.205647000	-2.068851000
H	0.262558000	3.323840000	-3.914930000
H	2.013614000	3.568901000	-3.788409000
H	0.950472000	4.042808000	-2.428630000
H	1.059654000	-1.255700000	5.274635000
H	0.736206000	1.311261000	3.795611000
H	1.379974000	-2.472227000	3.624533000
H	1.566405000	-3.663477000	1.541000000
H	0.536772000	2.429821000	5.502062000
H	0.946143000	-1.360576000	7.553100000
H	0.266163000	3.562523000	7.667471000
H	0.330896000	2.270701000	9.805277000
H	0.675392000	-0.207336000	9.725089000
H	1.478567000	-4.773279000	-0.354054000
H	1.063692000	2.354250000	-0.505067000
O	0.805507000	1.521290000	1.504954000
